# A Revised Time Tree of the Asterids: Establishing a Temporal Framework For Evolutionary Studies of the Coffee Family (Rubiaceae)

**DOI:** 10.1371/journal.pone.0126690

**Published:** 2015-05-21

**Authors:** Niklas Wikström, Kent Kainulainen, Sylvain G. Razafimandimbison, Jenny E. E. Smedmark, Birgitta Bremer

**Affiliations:** 1 Bergius Foundation, The Royal Swedish Academy of Sciences and Department of Ecology, Environment and Plant Sciences, Stockholm University, SE-10691, Stockholm, Sweden; 2 University of Bergen, University Museum of Bergen, The Natural History Collections, Post Box 7800, NO-5020 Bergen, Norway; Biodiversity Insitute of Ontario—University of Guelph, CANADA

## Abstract

Divergence time analyses in the coffee family (Rubiaceae) have all relied on the same Gentianales crown group age estimate, reported by an earlier analysis of the asterids, for defining the upper age bound of the root node in their analyses. However, not only did the asterid analysis suffer from several analytical shortcomings, but the estimate itself has been used in highly inconsistent ways in these Rubiaceae analyses. Based on the original data, we here reanalyze the divergence times of the asterids using relaxed-clock models and 14 fossil-based minimum age constraints. We also expand the data set to include an additional 67 taxa from Rubiaceae sampled across all three subfamilies recognized in the family. Three analyses are conducted: a separate analysis of the asterids, which completely mirrors the original asterid analysis in terms of taxon sample and data; a separate analysis of the Gentianales, where the result from the first analysis is used for defining a secondary root calibration point; and a combined analysis where all taxa are analyzed simultaneously. Results are presented in the form of a time-calibrated phylogeny, and age estimates for asterid groups, Gentianales, and major groups of Rubiaceae are compared and discussed in relation to previously published estimates. Our updated age estimates for major groups of Rubiaceae provide a significant step forward towards the long term goal of establishing a robust temporal framework for the divergence of this biologically diverse and fascinating group of plants.

## Introduction

The Rubiaceae (coffee family) are flowering plants and include more than 13.000 species [1]. They are highly diverse, not only in terms of their large number of species but also with respect to life forms, morphologies and their geographical distributions. Life forms span from annual and perennial herbs to large trees but we also find epiphytes, lianas, geofrutices, succulents, rheophytes and aquatic life forms [2]. Flowers in the family are generally pentamerous but tetramerous, trimerous, and pleiomerous flowers are spread across different tribes of the family [2], and they display a wide range of forms, sizes, and colors. Pollination in the family is mainly biotic, predominantly by insects, but bird-and bat pollination have been documented [2–3]. Fruit types include animal dispersed fleshy fruits and dry fruits such as nuts and capsules. The latter often have small and wind dispersed seeds [2, 4], and considerable diversity in form, size, texture, and color is displayed by the fleshy fruits indicating that other animals than birds are involved in their dispersal. Geographically, the family occurs on all continents, even on Antactica with a few species of *Coprosma*, *Galium*, *Nertera*, and *Sherardi*a [5], but a large proportion of extant species diversity is confined to tropical and subtropical regions [1–2]. They occupy a wide range of different habitats, from dry and desert like conditions to wet tropical rain forests, and their altitudinal ranges span from low altitude in tropical rainforests and mangrove coastline vegetation to alpine areas, above 4000m.

A growing understanding for the origins and evolution of this exceptional diversity has developed over the last 20 years. An accumulation of molecular data, coupled with methodological advances, have led to a large number of publications focusing on phylogenetic relationships in the family at different taxonomic levels (see [6], for a review). This development of a rigorous phylogenetic framework of the family has, in turn, led to a growing number of studies looking at the evolution of particular features in Rubiaceae in a phylogenetic context. This include characteristics such as lifeforms [7], ecological traits [4, 8–10], morphological traits [11–14], and broad-scale biogeographic patterns [15–20]. To further develop our understanding for the processes that have formed and shaped the biodiversity in Rubiaceae, we should correlate this biological evolution with other historical events, and develop a comprehensive temporal framework for the divergence of the group. Species divergences in Rubiaceae have, for example, been correlated with tectonic events [16], with the opening and closure of continental dispersal routes [17, 19], and the colonization of oceanic islands [21], but correlations such as these critically depend on the ages inferred for the taxa involved.

Divergence times have been inferred for the Rubiaceae but, with one exception, all such analyses have focused on smaller more restricted groups inside the family, and have not provided comprehensive estimates for Rubiaceae divergence time as a whole [16–17, 19, 22–24]. Antonelli et al. [16], for example, investigated the influence of the Andean uplift on the diversification of Rubiaceae in the neotropics. Although representatives of the subfamilies Ixoroideae and Rubioideae were included in their taxon sample, it was strongly biased towards neotropical taxa of the Cinchonoideae. Primary focus was in fact only on the two tribes Cinchoneae and Isertieae, and age estimates were not reported for individual tribes outside of the subfamily Cinchonoideae [16]. Also Manns et al. [17] had a taxon sample biased towards neotropical taxa while investigating the historical biogeography of the subfamily Cinchonoideae. Compared to the analyses by Antonelli et al. [16] they included a number of additional taxa from the subfamilies Rubioideae and Ixoroideae, but age estimates for individual tribes in these two subfamilies were not reported in their analyses either [17]. A similar situation is seen in Nie et al. [23] and their analyses of pantropical disjunctions in *Paederia*, but their focus on the genus *Paederi*a resulted in subfamily Rubioideae being much better sampled than subfamilies Ixoroideae and Cinchonoideae [23]. The most ambitious and comprehensive attempt at estimating divergence times in Rubiaceae is without doubt the analyses by Bremer & Eriksson [25], and contrary to all other analyses they reported age estimates across different subgroups of the family. They conducted two separate analyses. The first included 534 Rubiaceae taxa from 329 genera and used up to five different chloroplast markers. This analysis was done using MrBayes and only focused on reconstructing the phylogenetic relationships in the family. Their second analyses, where divergence times also were inferred, used a “scaled-down” dataset of 173 Rubiaceae taxa representing 150 genera and a large proportion of recognized subgroups of the family. They reported crown-and stem group age estimates not only for the three subfamilies Rubioideae, Ixoroideae, and Cinchonoideae, but also for most of the tribes recognized in each of the three subfamilies at the time of their analyses [25].

A problem shared by previous divergence-time analyses of Rubiaceae is that they have all relied on the same source of information for defining the upper age bound of the root node in their analyses. Focused on the Rubiaceae, or subgroups within the family, they were all rooted on outgroup taxa from remaining families of the Gentianales. Bremer et al. [26] reported an age estimate for the Gentianales crown group of 78 Myr, and their Gentianales crown group age estimate has been used directly, or indirectly, as the basis for a secondary calibration of the root node in all divergence-time analyses of the Rubiaceae [16–17, 19, 22–25]. Having used the same root calibration information, one would expect the different analyses to have resolved early divergence events in the Rubiaceae, events close to the root, at similar ages, but this is not the case. This is a result of the different ways in which the 78 Myr calibration point was applied in their different analyses. Antonelli et al. [16], for example, used it to enforce a maximum age constraint of 78 Myr for the root node, effectively prohibiting the analyses to resolve the Gentianales as being any older. This example was followed by Huang et al. [22] who applied a fixed calibration point to their root node at 78 Myr, and by Nie et al. [23] who used a normally distributed prior for their root age at 78 ± 1 Myr. Also Manns et al. [17] enforced a maximum age constraint for their root node in much the same way, but tried to account for uncertainties in the estimate reported by Bremer et al. [26] by applying a maximum age constraint of 88 Myr for their root node. Bremer & Eriksson [25] took also other age estimates of the Gentianales crown group into account, such as those reported by Davies et al. [27] and by Wikström et al. [28–29], and they applied a normally distributed prior with a mean of 78 Myr and a standard deviation of 10 Myr on their root node. This conservative approach deviates significantly from that used in all the other analyses and result in a 95% confidence interval for their Gentianales root node age between 58–98 Myr [25]. Smedmark et al. [19, 24] used age distributions reported by Bremer & Eriksson [25] for the Rubiaceaae [19], and the Rubioideae [24] crown groups for constraining their root nodes and only indirectly relied on the age estimate from Bremer et al. [26].

Bremer et al. [26] inferred ages for a much broader group of plants than Rubiaceae and they used a 111-taxon data set to infer divergence times among all major groups and orders of asterid flowering plants. Their analyses included six plastid markers and were conducted with semiparametric rate smoothing by penelized likelihood [30]. Although they explored the effects that various sources of error had on their age estimates, proper error bounds were never reported, and this could perhaps explain why the divergence-time analyses of the Rubiaceae have used the Gentianales crown group age estimate in so many different ways [16–17, 19, 22–25]. A second problem with the analyses by Bremer et al. [26] is their dependence on a “relaxed-clock” model that makes an a priori assumption of autocorrelation of rates among branches [30]. An influence of life history characteristics, such as generation time and metabolic rate, on mutation rates have been used to biologically motivate this type of assumption, but significant jumps or shifts in rates over the tree will likely not be handled well using smoothing methods such as NPRS or penelized likelihood [31].

A complication of any molecular dating analysis is that a set of minimum age constraints is not enough in order for the analysis to converge on a unique solution [32]. Somehow we need to restrict the range of solutions of the entire tree by also enforcing some kind of maximum age constraint for at least one of the nodes in our tree. Commonly a maximum age constraint is applied to the root node of the analyses. However, the fossil record is generally of little use for defining maximum age constraints, and we are left with the option of using a maximum age constraint for our root node based on the results from some previous analysis. This has, with no exception, been the case for previous divergence-time analyses in the Rubiaceae and they have all used the 78 Myr Gentianales crown group age estimate reported by Bremer et al. [26] for this purpose. As pointed out, the analyses behind this estimate suffered from several analytical shortcomings. Equally problematic is that the estimate itself has been used in highly inconsistent ways by previous divergence-time analyses in the Rubiaceae [16–17, 22–23, 25]. To overcome these problems we here reanalyze the divergence times of asterids using the original data from Bremer et al. [26]. We also include an additional 67 taxa representing all three subfamilies and most tribes recognized in the Rubiaceae [25]. By bridging the analysis of the Rubiaceae with the asterid analysis, we can estimate divergence times for Gentianales and major groups of Rubiaceae whithout being dependant on any secondary calibration point. Our primary aims with the analyses are to establish an updated and improved temporal framework for the divergences of Gentianales and major groups of Rubiaceae, and to obtain secondary calibration points within the Rubiaceae, calibration points that will be important for future analyses focusing on smaller groups within the family.

## Material and Methods

### Taxon sample

We used the complete data set from Bremer et al. [26], including 111 taxa representing all major groups and orders, and most recognized families of the asterids. Three outgroup taxa were inluded, two from the rosids (*Viti*s and *Dipentodon*) and one from the Saxifragales (*Paeonia*). In addition, 67 taxa from the Rubiaceae were selected representing all tribes currently recognized in the family [25, 33–36]. The Seychellois genus *Glionnetia*, currently unclassified at tribal level, was also included in the study, as it has either unsupported or unresolved position in the Vanguerieae alliance [33, 37].

### DNA Extraction, amplification, sequencing and sequence assembly

Total DNA was extracted from herbarium specimens and/or silica-dried material using a standard cetyltrimethyl-amonium bromide CTAB protocol [38]. Extracted DNA was cleaned using QIAquick PCR cleaning kit (Qiagen, Hilden, Germany) following the protocol specified by the manufacturer. Plastid regions *rbcL*, *ndhF*, *matK*, *trnV*, *rps16*, and *trnL-F* were amplified and sequenced using primers listed in Table 1. Reactions were carried out in 50-μL aliquots including: 5 μL 10x Paq5000 reaction buffer, 4 μL 10 mM dNTP mix, 0,5 μL Paq5000 DNA polymerase (5 U μL^-1^), 0,5 μL of each primer (20 μM), 0,5 μL bovine serum albumin (BSA; 1%), 1 μL DNA template, and sterilized water. Following amplification the PCR products were cleaned using the MultiScreen Separation System (Millipore, Billerica, Massachusetts, U.S.A.), sequenced with the BigDye terminator cycle sequencing kit, and analyzed on an ABI PRISM 3100 Genetic Analyzer (Applied Biosystems, Foster City, California, U.S.A.).

**Table 1:** Primers used for amplification and sequencing of new sequences in this study.

Region	Primer	Primer sequence from the 5' end	Reference
*rps16*	F	GTG GTA GAA AGC AAC GTG CGA CTT	Oxelman et al. [39]
	2R	TCG GGA TCG AAC ATC AAT TGC AAC	Oxelman et al. [39]
*rbcL*	5'F	ATG TCA CCA CAA ACA GAA ACT AAA GC	Bremer et al. [40]
	bs427F	GCT TAT ATT AAA ACC TTC CAA GGC CCG CC	Bremer et al. [40]
	Z1020R	ATC ATC GCG CAA TAA ATC AAC AAA ACC TAA AGT	Zurawski, DNAX Research Institute
	3'R	CTT TTA GTA AAA GAT TGG GCC GAG	Bremer et al. [40]
*ndhF*	2F	ATG GAA CAG ACA TAT CAA TAC GG	Rydin et al. [41]
	720F	GCA CAA TTT CCC CTT CAT GTA TGG	Rydin et al. [41]
	1320F	GGG ATT AAC YGC ATT TTA TAT GTT TCG	Rydin et al. [41]
	1000R	CCT AGA GCT AGC ATC ATA TAA CCC	Rydin et al. [41]
	1700R	AGT ATT ATC CGA TTC ATA AGG AT	Rydin et al. [41]
	2280R	AAG AAA AGA TAA GAA GAG ATG CG	Rydin et al. [41]
*trnV*	1bF	GAA CCG TAG ACC TTC TCG GTA AAA CAG ATC	Bremer et al. [40]
	3F	GTG TAA ACG AGT TGC TCT ACC	Bremer et al. [40]
	4R	GAA CCA ATG ACT CCC GCC GTA TG	Bremer et al. [40]
	6bR	GAA GAA ATG ACC TTA AAT CTT TGT G	Bremer et al. [40]
trnTF	a1F	ACA AAT GCG ATG CTC TAA CC	Bremer et al. [40]
	cF	CGA AAT CGG TAG ACG CTA CG	Taberlet et al. [42]
	iR	CCA ACT CCA TTT GTT AGA AC	Bremer et al. [40]
	fR	ATT TGA ACT GGT GAG ACG AG	Taberlet et al. [42]
*matK*	*trnK*3914F	TGG GTT GCT AAC TCA ATG G	Johnson and Soltis [43]
	1F	ACT GTA TCG CAC TAT GTA TCA	Sang et al. [44]
	1198F	CTG TGT TAG ATA TAC NAA TAC CCC	Andersson and Antonelli [45]
	1760F	TRG GCT ATC TTT CAA GYG TGC G	Kainulainen et al. [46]
	807R	ACT CCT GAA AGA TAA GTG GA	Kainulainen et al. [46]
	4bR	GCR TCT TTT ACC CAA TAG CGA AG	Kainulainen et al. [46]
	6R	TTC TAG MAT TTG ACT CCG TAC C	Bremer et al. [40]
	*trnK*2R	AAC TAG TCG GAT GGA GTA G	Johnson and Soltis [43]

Sequences were edited and assembled using the Staden Package [47, 48] and Seaview version 4.3.1 [49]. All regions were aligned using the program MUSCLE version 3.8.31 [50]. Before submitting sequences to MUSCLE for sequence alignments they were sorted by sequence length using the program USEARCH version 5.2.32 [51].

### Phylogenetic and divergence time analyses

Analyses were conducted using Markov Chain Monte Carlo methods [52] in BEAST v.1.7.5 [53]. In the MCMC:s the data were partitioned into coding (*rbcL*, *ndhF*, *matK*) and non-coding (*rps16*, *trnL-F*, *trnV*) regions and each region was allowed partition-specific parameters [54, 55]. The substitution model for each partition was chosen based on the corrected Akaike information criterion (AICc) as calculated using MrAIC v.1.4.4 [56] and PHYML v.3.0 [57]. The GTR+I+Γ was selected for the coding and GTR+Γ for the non-coding partition. A specific model for the distribution of substitution rates across branches was not selected in the BEAST analyses. Instead a model averaging approach was adopted, as outlined by Li & Drummond [58]. Averaging was done across two different distributions that are implemented in BEAST v.1.7.5 to model the variation in rate of substitution across branches: the lognormal and the exponential distributions [53]. This model averaging approach is an alternative approach for dealing with model selection uncertainty. Each model is sampled in proportionn to its posterior probability, and the resulting estimates are weighted according to the probabilities of the models [58]. A starting tree compatible with the specified age constraints (see below) was generated using r8s version 1.8 [59]. Three chains, each including 100,000,000 generations were run and stationarity of each chain was assessed using Tracer v.1.5 [60]. Following stationarity, trees and parameters were sampled every 1,000 generations in each chain. Convergence of the chains was assessed by comparing their posterior distributions, and trees and parameter samples from each chain were subsequently pooled in order to obtain an effective sample size (ESS) of more than 200 samples for all parameters.

To better understand how specific differences in the analytical protocols of Bremer et al. [26] and the ones used here affect our age estimates we conducted two different analyses. In the first, referred to as the separate analysis, the asterids and the Gentianales were each analyzed separately. The asterids were analyzed first, using a taxon sample identical to that used by Bremer et al. [26], and the resulting Gentianales crown group age estimate was subsequently used as a secondary calibration point for the root node in the separate analysis of the Gentianales. In the second analysis, referred to as the combined analysis, all taxa were analyzed simultaneously and without the inclusion of any secondary calibration points.

### Fossil data

The early fossil record of the asterids was recently surveyed and evaluated by Martínez-Millan [61], with the primary focus of finding the oldest fossils ever reported for each of the 100–104 families recognized in the group. In her survey, she evaluated every fossil with respect to the reliability of its identification and phylogenetic placement following 8 criteria. Each one of the criteria was evaluated as provided or not provided by the authors, and they were ranked in order of decreasing reliability. Fossils that fulfilled the first three criteria she accepted as representing reliable records. These were: (1) inclusion of the fossil in a phylogenetic analysis, (2) discussion of key characters that place the fossil in a group, (3) list of key characters that place the fossil in the group [61]. Records that passed this filtering, and that she thereby considered reliable, were subsequently incorporated as minimum age indicators in a phylogeny of the asterids following the approach developed by Crepet et al. [62]. She assigned ages of the fossils to the most recent accepted age of the sediments in which the fossils were found, and for the purpose of assigning numerical dates to time periods she used the upper bound (end) of that period as indicated by Gradstein et al. [63]. Altogether, the approach adopted by Martínez-Millan [61] is in line with the best practices for justifying fossil calibrations [64].

We have followed the approach adopted by Martínez-Millan [61], and in total we defined 17 minimum age constraints in our analyses distributed across 9 different orders of the asterids. All of these were defined as uniform priors with their minimum ages based on the ages of the fossils and their maximum ages set to the same maximum age as our root node (see below). Twelve constraints were based on the minimum age estimates reported by Martínez-Millan [61]; two were based on more recently reported Asteraceae and Apocynaceae fossils, both from the Middle Eocene [65, 66] and that were never considered by Martínez-Millan [61]; and three were based on Rubiaceae fossils, each representing one of the three subfamilies recognized in the family [67–70]. Compared to Martínez-Millan [61] we used a more up to date geologic time scale [71]. Graham [72] reviewed the fossil record of the Rubiaceae in 2009, and compiled a long list of genera reported from the fossil record and that he classified as either accepted, pending, or not accepted. However, he never provided any arguments for why he accepted the phylogenetic placement of some fossils while rejecting others. Given the approach adopted here for justifying fossil-based calibrations, we have chosen not to consider the assignments indicated by Graham as sufficient evidence for using the fossils as minimum-age constraints in our analyses. Details of all fossil taxa used, their ages, from where and by whom they were documented, and their phylogenetic placements in our analyses are given in Table 2 and discussed at more length in the (S1 Text). In addition, a uniform prior age distribution with a maximum age of 128 Myr was applied to our root node. This upper limit is based on the appearance of eudicots in the fossil record, and their unique tricolpate pollen. Strictly speaking, fossils can not provide maximum age constraints in divergence-time analyses. However, tricolpate pollen grains have up until now never been recovered from sediments older than the Late Barremian, yet they are produced in abundance, they are easily fossilized and identified, and following their first appearance they quickly became abundant in the fossil record [88]. These observations have been used to justify their use as a maximum age indicator, and their Late Barremian appearance have commonly been used to constrain the maximum age of the eudicots [89–93]. We have accepted this argument and applied their first occurrence as a maximum age constraint for the asterid stem lineage, a group smaller than, and nested well inside the eudicots [94]. The best-dated early record of tricolpate pollen is from the Cowleaze Chine Member of the Vectis Formation of the Isle of Wight [95], and following Clarke et al. [96] this is from the M1n polarity chron at the top of the Barremian. Recent updates place this chron at 126,3–128,3 Mya [97], and our maximum age prior correspond to the maximum age of this chron.

**Table 2:** Fossil taxa used for specifying prior age distributions in the analyses.

Order/Taxon	Age (Myr)	Fossil strata/locality	Reference	Phylogenetic placement
Cornales				
*Tylerianthus crossmanensis*	Late Cretaceous, Turonian (90 Myr)	South Amboy Fire Clay, Raritan Formation, New Jersey, USA	Gandolfo *et al*. [73]	Cornales (crown group; node 177)
Ericales				
*Pentapetalum trifasciculandricus*	Late Cretaceous, Turonian (90 Myr)	South Amboy Fire Clay, Raritan Formation, New Jersey, USA	Martínez-Millan *et al*. [74]	Pentaphyllaceae (crown group; node 164)
*Parasaurauia allonensis*	Late Cretaceous, Campanian (72 Myr)	Buffalo Creek Member, Gaillard Formation, Georgia, USA	Keller *et al*. [75]	Actinidiaceae (stem lineage; node 169, Fig 1; node 168, Fig 3)
Aquifoliales				
*Ilex hercynica*	Early Paleocene (62 Myr)	Gonna-Walkmühle (Abschnitt II), Sangerhausen, Sachsen-Anhalt, Germany	Mai [76]	*Ilex* (stem lineage; node 152)
Apiales				
*Dendropanax eocenensis*	Middle Eocene (38 Myr)	Clairborne Formation, Tennessee, USA	Dilcher & Dolph [77]	Araliaceae/Apiaceae (stem lineage; node 139)
*Torricellia bonesii*	Late Paleocene (56 Myr)	Sentinel Butte Formation, Almont North Dakota, USA	Manchester *et al*. [78]	*Toricellia* group (stem lineage; node 137)
Dipsacales				
*Diplodipelta reniptera*	Late Eocene (34 Myr)	Florissant Formation, Colorado, USA	Manchester & Donoghue [79]	Linnaeaceae (stem lineage; node 147)
Asterales				
*Raiguenrayun cura*	Middle Eocene; 47 Myr^1^	Río Pichileufú, Huitrera Formation, Río Negro Province, Argentina	Barreda *et al*. [65]	Asteraceae (stem lineage; node 132)
Gentianales				
*Cypselites sp*.	Middle Eocene (47 Myr)	Messel Formation, Darmstadt, Germany	Collinson *et al*. [66]	Apocynaceae (stem lineage; node 111)
*Scyphiphora sp*. (pollen)	Early Miocene (16 Myr)	Drill holes on Eniwetok Atoll, Northern Marshall Islands at depth 2,440–2,470 feet	Leopold [66]	*Scyphiphora* (stem lineage; node 62)
*Cephalanthus pusillus*	Late Eocene (34 Myr)	Sandgrube Nobitz and Tagebau Perez, Weißelster Basin Flora, Germany	Mai & Walther [68, 69]	*Cephalanthus* (stem lineage; node 79)
*Morinda chinensis*	early Late Eocene (38 Myr)	Coal-bearing series, Changchang Formation, Hainan Island, China	Shi *et al*. [70]	Morindeae (stem lineage; node 106)
Solanales				
*Solanispermum reniforme*	Middle Eocene (46 Myr)^2^	Poole Formation, Bracklesham Group, Dorset, UK	Chandler [80]	Solanaceae (stem lineage; node 14)
Lamiales				
*Melissa parva*	Late Eocene (34 Myr)^3^	Bembridge Marls Member, Bouldnor Formation, Isle of White, UK	Reid & Chandler [81]	Lamiaceae (stem lineage; node 29, Fig 1; node 31, Fig 3)
*Radermachera pulchra*	Late Eocene (34 Myr)^3^	Bembridge Marls Member, Bouldnor Formation, Isle of White, UK	Reid & Chandler [81]	Bignoniaceae (stem lineage; node 33)
*Acanthus rugatus*	Late Eocene (34 Myr)^3^	Bembridge Marls Member, Bouldnor Formation, Isle of White, UK	Reid & Chandler [81]	Acanthaceae (stem lineage; node 29)
Vahliaceae				
*Scandianthus major*	Late Santonian (84 Myr)	Åsen, Scania, S. Sweden	Friis & Skarby [82]	Vahliaceae (stem lineage, node 9)

**Fig 1:**
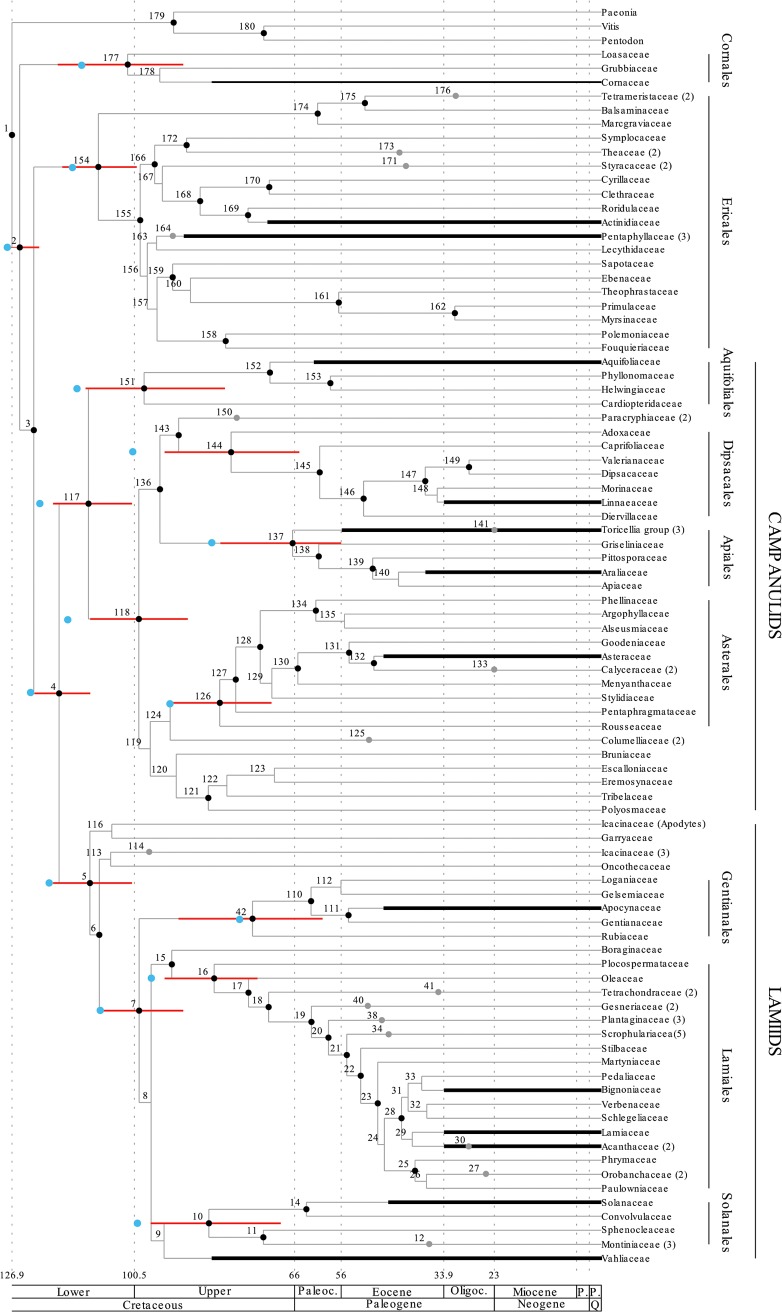
Chronogram of the asterids resulting from the separate analysis and calibrated against The Geologic Time Scale [71]. Nodes are numbered from 1 to 180 (42 to 112; Fig 2) and detailed results (mean divergence time and credibility intervals estimated by the 95% HPD) for each node is reported in the (S2 Table). Nodes with small black bullets are well supported in the phylogenetic analyses (BPP equal to or greater than 0.95). Ages for major groups and orders of the asterids are compared to those reported by Bremer et al. [26] in Table 3. Credibility intervals for these nodes are indicated by red bars, and the point estimate reported by Bremer et al. [26] is indicated by a light blue dot. Fossil based age estimates for selected groups are indicated by thick black bars, and these estimates were included in the analyses as uniform priors with minimum ages set to the age of the fossil (see Table 2). Gray dots indicate the crown node position for groups that have been collapsed in the figure. The number in parenthesis next to the taxon name indicate how many taxa each of these collapsed groups included in the analysis. See (S1 Table) for details of all the taxa that were included in the analysis.

**Fig 2:**
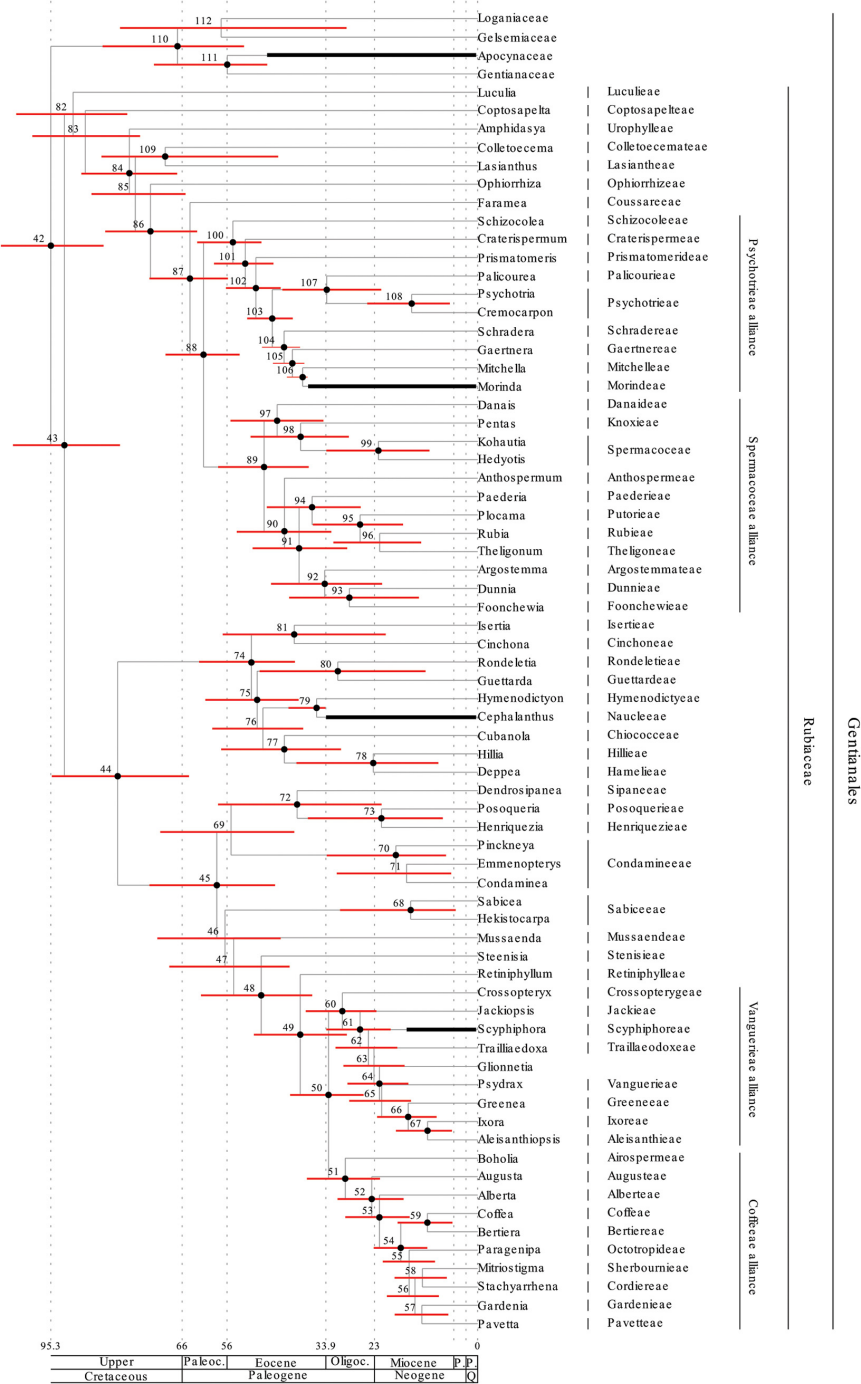
Chronogram of the Gentianales resulting from the separate analysis and calibrated against The Geologic Time Scale [71]. Nodes are numbered from 42 to 112 and detailed results (mean divergence time and credibility intervals estimated by the 95% HPD) for each node is reported in the (S2 Table). Credibility intervals are also indicated in the figure by red bars. Nodes with small black bullets are well supported in the phylogenetic analyses (BPP equal to or greater than 0.95). Fossil based age estimates that were used in the analyses as minimum age constraints are indicated by thick black bars (see Table 2 for details). In addition a normally distributed secondary root calibration point with mean 75 Myr and standard deviation 7,7 Myr was enforced in the analysis. This secondary calibration point was based on the results from the first asterid analysis and correspond to a 95% credibility interval between 60 and 90 Myr. The three subfamilies Rubioideae, Cinchonoideae and Ixoroideae, and the Psychotrieae, Spermacoceae, Vanguerieae, and Coffeeae alliances are indicated in the tree. Current tribal assignment of each included taxa is indicated to the right of the taxon names.

**Fig 3:**
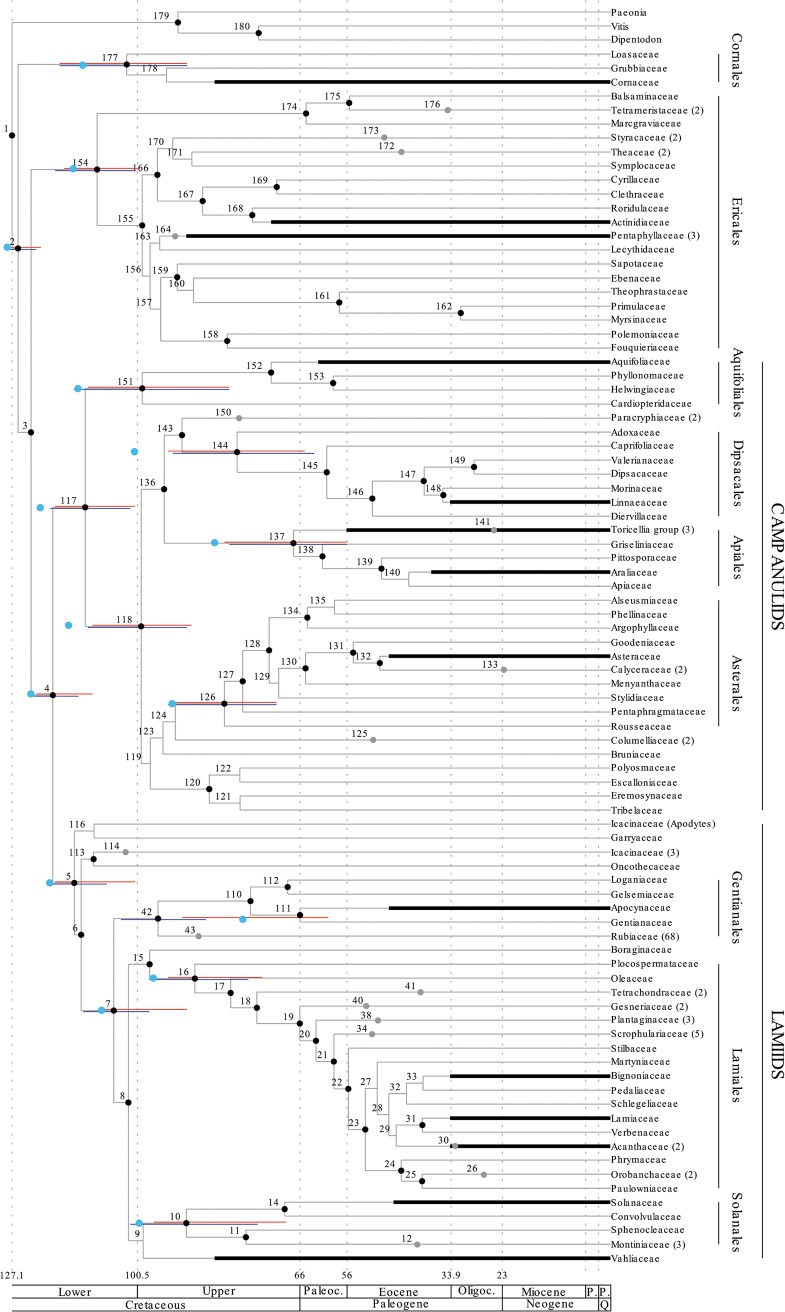
Chronogram of the asterids resulting from the combined analysis and calibrated against The Geologic Time Scale [71]. Nodes are numbered from 1 to 180 (42 to 112; Fig 4) and detailed results (mean divergence time and credibility intervals estimated by the 95% HPD) for each node is reported in the (S2 Table). Nodes with small black bullets are well supported in the phylogenetic analyses (BPP equal to or greater than 0.95). Ages for major groups and orders of the asterids are compared to those reported by Bremer et al. [26] in Table 3. Credibility intervals for these nodes resulting from the combined analysis are indicated by blue bars, and the point estimate reported by Bremer et al. [26] is indicated by a light blue dot. Credibility intervals resulting from the separate analysis of the asterids (Fig 1) are included also in this figure and indicated by red bars. This provide a visualization of the differences in age estimates resulting from the two analyses. Fossil based age estimates for selected groups are indicated by thick black bars, and these estimates were included in the analyses as uniform priors with minimum ages set to the age of the fossil (see Table 2). Gray dots indicate the crown node position for groups that have been collapsed in the figure. The number in parenthesis next to the taxon name indicate how many taxa each of these collapsed groups included in the analysis. See (S1 Table) for details of all the taxa that were included in the analysis.

**Fig 4:**
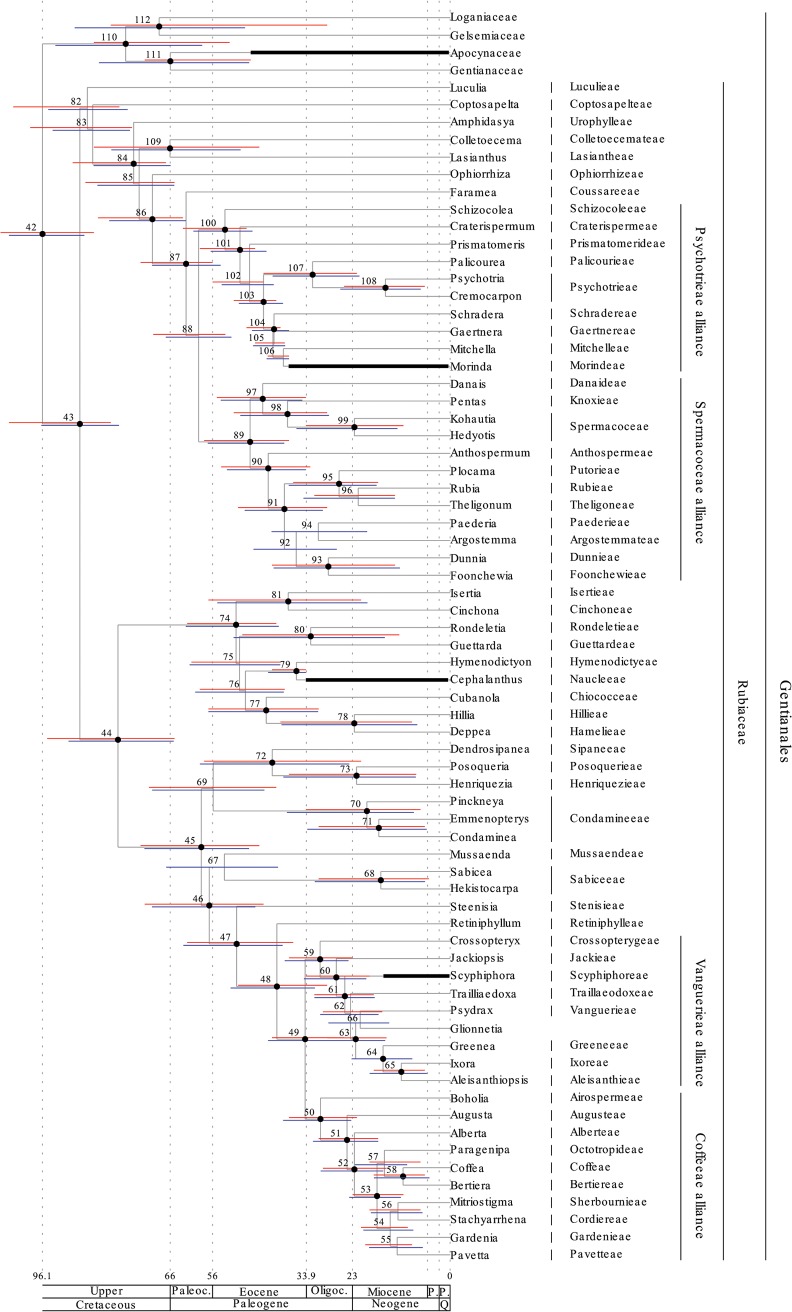
Chronogram of the Gentianales resulting from the combined analysis and calibrated against the geologic time scale [71]. Nodes are numbered from 42 to 112 and detailed results (mean divergence time and credibility intervals estimated by the 95% HPD) for each node is reported in the (S2 Table). Credibility intervals resulting from the combined analysis are indicated in the figure by blue bars. Credibility intervals resulting from the separate analysis of the Gentianales (Fig 2) are included also in this figure and indicated by red bars. This provide a visualization of the differences in age estimates resulting from the two analyses. Nodes with small black bullets are well supported in the phylogenetic analyses (BPP equal to or greater than 0.95). Fossil based age estimates that were used in the analyses as minimum age constraints are indicated by thick black bars (see Table 2 for details). The three subfamilies Rubioideae, Cinchonoideae and Ixoroideae, and the Psychotrieae, Spermacoceae, Vanguerieae, and Coffeeae alliances are indicated in the tree. Current tribal assignment of each included taxa is indicated to the right of the taxon names.

Note that age assignments of the fossils follow the most recent accepted ages for the sediments in which the fossils have been found, not the ages given in the original reports. Following best practice guidelines [64], numerical ages assigned to different time periods correspond to their upper bounds as defined in The Geologic Time Scale 2012 [71]. Node numbers refer to those indicated in (Figs 1–4).

^1^ Minimum age is constrained by radiometric dating [83].

^2^ Upper bound of the Poole Formation at the locality is 46 Myr [84].

^3^ Bembridge Flora recently placed in the Late Eocene [85–87].

## Results

### Sequence data

Sequence data from the plastid regions *rbcL*, *ndhF*, *matK*, *trnV*, *rps16*, and *trnL-F* intron were successfully generated. In total 4 sequences of *rbcL*, 8 sequences of *ndhF*, 45 sequences of *matK*, 58 sequences of *trnV*, 4 sequences of *rps16*, and 3 sequences of *trnL-F* intron were newly generated. Sequences are deposited at the EMBL Nucleotide Sequence Database and their EMBL accession numbers are reported in the (S1 Table). The compiled separate datasets comprised 114 taxa and 11,407 characters (asterids) and 72 taxa and 11,470 characters (Gentianales). The combined dataset comprised 181 taxa and 13,332 characters. All three data sets are available in the (S1, S2, S3 Datasets).

### Relationships

Results are shown in (Figs 1–4) and both the separate (Figs 1 and 2) and the combined analysis (Figs 3 and 4) resulted in highly resolved and mostly well supported consensus trees. Nodes with a bayesian posterior probability (BPP) equal to or greater than 0.95 are indicated with black dots in (Figs 1–4). Only minor incongruencies are seen in the results from the separate and the combined analyses. Differences in non-Gentianales taxa include the position of Martyniaceae (Lamiales); the position of Bruniaceae and the position of Argophyllaceae (Asterales); and the position of Styracaceae (Ericales). Differences in the Gentianales include the position of *Mussaend*a (Mussaendeae; Ixoroideae); the position of *Glionneti*a (Vanguerieae alliance); and the position of *Paederi*a (Paederieae; Spermacoceae alliance). As a rule, at least one of the two alternative positions for the taxon involved is poorly supported in each of these incongruencies.

### Age estimation

Results from the divergence-time analyses are presented in (Figs 1–4) in the form of chronograms calibrated against the geological time scale [71]. Age estimates for major groups and orders of the asterids are compared to those reported by Bremer et al. [26] in Table 3, and estimates for major groups of Rubiaceae are compared to those reported by Bremer & Eriksson [25], Antonelli et al. [16], and Manns et al. [17] in Table 4. Detailed age estimate for all nodes are also given in the (S2 Table). Chronograms for the asterids are shown in (Fig 1; separate analysis) and (Fig 3; combined analysis). Credibility intervals, as estimated by the 95% highest posterior density (95% HPD) from the separate analysis (red bars in Figs 1 and 3) and from the combined analysis (blue bars in Fig 3), are indicated for major groups and orders of the asterids. These groups correspond to those for which age estimates were reported by Bremer et al. [26], and the point estimates reported in that study are also indicated in (Figs 1 and 3) with light blue dots. Chronograms for the Gentianales are shown in (Fig 2; separate analysis) and (Fig 4; combined analysis). Credibility intervals from the separate (red bars in Figs 2 and 4) and the combined (blue bars in Fig 4) are indicated for nodes that were resolved in both analyses.

**Table 3:** Estimated crown and stem group ages for major groups and order of the asterids.

		Present analysis (separate)		Present analysis (combined)		Bremer et al. [26]	
Node nr.		Mean age	Mean age	Mean age	Mean age		
		(95% HPD interval)	(95% HPD interval)	(95% HPD interval)	(95% HPD interval)	Lineage age	Lineage age
	Clade/Taxon	stem	crown	stem	crown	stem	crown
2	**Asterids**	126 (123–128)	125 (121–128)	127 (124–128)	125 (122–128)	–	128
4	**Core Asterids**	122 (117–126)	116 (110–122)	123 (119–126)	118 (113–123)	127	123
5	**Lamiids**	116 (110–122)	110 (101–118)	118 (113–123)	114 (107–119)	123	119
7	**Core Lamiids**	107 (97–115)	98 (87–107)	112 (106–118)	105 (98–112)	119	108
10	**Solanales**	94 (85–103)	84 (69–97)	99 (89–107)	89 (75–102)	106	100
16	**Lamiales**	92 (83–101)	83 (74–94)	98 (89–106)	88 (77–98)	106	97
42	**Gentianales** ^**1**^	99 (90–108)	75 (60–91)	105 (98–112)	96 (86–104)	108	78
42	**Gentianales** ^**2**^	–	95 (84–106)	105 (98–112)	96 (86–104)	108	78
117	**Campanulids**	116 (110–122)	110 (101–118)	118 (113–123)	111 (102–119)	123	121
118	**Core campanulids**	110 (101–118)	100 (89–110)	111 (102–119)	100 (90–111)	121	114
126	**Asterales**	93 (81–104)	82 (71–93)	93 (82–103)	83 (71–94)	112	93
137	**Apiales**	95 (83–106)	67 (56–82)	95 (83–107)	68 (56–81)	113	84
144	**Dipsacales**	91 (79–103)	80 (65–94)	91 (77–104)	79 (63–93)	111	101
151	**Aquifoliales**	110 (101–118)	97 (81–111)	111 (102–119)	98 (81–114)	121	113
154	**Ericales**	122 (117–126)	108 (100–116)	123 (119–126)	109 (101–118)	127	114
177	**Cornales**	125 (121–128)	103 (90–117)	125 (122–128)	103 (90–117)	128	112

Age estimates from each of the two analyses (separate and combined) are compared to those reported by Bremer et al. [26]. Node numbers refer to numbered nodes in (Figs 1 and 3 and S2 Table).

^1^ Estimates in the separate analysis are from the asterid analysis.

^2^ Estimates in the separate analysis are from the Gentianales analysis.

**Table 4:** Estimated ages (Myr) for the Gentianales and major groups of the Rubiaceae.

Node nr.		Mean age	Mean age	Mean age	Mean age	Mean age
		(95% HPD interval)	(95% HPD interval)	(95% HPD interval)	(95% HPD interval)	(95% HPD interval)
	Clade/Taxon	separate analysis	combined analysis	Bremer & Eriksson [25]	Antonelli et al. [16]	Manns et al. [17]
42	Gentianales	95 (84–106)	96 (86–104)	90 (77–105)	78	…
43	Rubiaceae	92 (80–104)	87 (78–96)	87 (73–101)	66 (63–69)	85 (81–88)
44	Ixoroideae + Cinchonoideae	80 (65–95)	78 (65–90)	73 (58–89)	63 (60–66)	78 (72–84)
45	Ixoroideae	59 (45–73)	59 (47–72)	60 (46–74)	48 (44–52)	65 (57–74)
49	Coffeeae + Vanguerieae alliances	40 (29–50)	35 (27–43)	37 (28–47)	…	…
50	Coffeeae alliance	33 (25–42)	31 (23–39)	22 (13–33)	…	…
59	Vanguerieae alliance	30 (23–38)	31 (24–39)	29 (20–39)	…	…
74	Cinchonoideae	51 (41–62)	51 (40–62)	39 (28–52)	51 (48–55)	57 (50–66)
84	Rubioideae	78 (67–89)	75 (66–84)	78 (65–91)	48 (43–52)	70 (64–76)
88	Psychotrieae + Spermacoceae alliances	61 (53–70)	59 (52–67)	63 (52–75)	…	…
89	Spermacoceae alliance	48 (38–58)	47 (39–57)	55 (45–65)	…	…
100	Psychotrieae alliance	55 (48–63)	53 (47–61)	49 (35–61)	…	…

Estimates from both the separate and the combined analysis are reported and compared to those from Bremer & Eriksson [25], Antonelli et al. [16], and Manns et al. [17]. Credibility intervals are indicated by the 95% highest posterior densities (HPD). Clade/Taxon names follow those given in (Figs 2 and 4), and node numbers refer to numbered nodes in (Fig 4 and S2 Table; combined analysis).

## Discussion

### Relationships

Overall relationships of the asterids in our analyses are highly congruent with the relationships obtained and used in the dating analysis by Bremer et al. [26]. Cornales and Ericales are resolved as successive sister-groups to the remaining asterids, which in turn are resolved into two large sister groups, the campanulids and the lamiids. These four groups were recognized already by early molecular analyses [40, 98, 99], and have remained unchallenged in more recent large-scale analyses of angiosperms [100–103]. Within these groups we see some minor differences in the relationships obtained in the present analyses compared to those reported by Bremer et al. [26], and to the ones obtained in more recent analyses of these groups [104–107], but most of these differences should have marginal effects on the age estimates for larger groups and orders of the asterids. One possible exception is the Ericales, where relationships among recognized families have been difficult to resolve [104]. Some of the oldest asterid fossils are from the Ericales, specifically from the Actinidiacae and the Pentaphyllaceae [61], and their specific relationships within the Ericales may affect how ages are resolved, not only within the Ericales, but to some extent also in other early diverging asterid groups. Schönenberger et al. [104], for example, resolved the Pentaphyllaceae in a slightly more derived position within the Ericales than obtained in our analyses, and if correct would imply that Ericales and early asterid divergences could be older than indicated by our results.

In Rubiaceae, the three subfamilies Rubioideae, Cinchonoideae, and Ixoroideae are recovered and well supported, and Cinchonoideae are resolved sister to the Ixoroideae (Figs 2 and 4). These relationships agree well with previous analyses looking at deep divergences in the family [25, 34, 108]. The two tribes Luculieae (*Luculia*) and Coptosapelteae (*Coptosapelta*) are resolved as successive sistergroups to the subfamily Rubioideae. These relationships agrees with those obtained by Manns et al. [17], but they are not well supported. Rydin et al. [34] resolved the two tribes together as sisters and in an unresolved trichotomy together with Rubioideae and a clade including Cinchonoideae and Ixoroideae, but also they failed to obtain support for the relationships of these tribes.

Also relationships within two of the three subfamilies are highly congruent with recent analyses focusing on these groups. Manns et al. [17] and Kainulainen et al. [33] are the most comprehensive and recent analyses of the Cinchonoideae and the Ixoroideae, and relationships obtained here are entirely consistent with those indicated in their analyses. Manns et al. [17] failed to find support for early divergences in the Cinchonoideae and corresponding problems are seen in our results (Figs 2 and 4). In Ixoroideae, the separate analysis of the Gentianales (Fig 2) and the combined analysis (Fig 4) resolve the relationships of Sabiceeae (*Sabice*a and *Hekistocarpa*) and Mussaendeae (*Mussaenda*) in slightly different positions, but none of the alternative positions are well supported. The separate analysis place the two tribes as successive sister groups to a large clade including the Vanguerieae alliance (node 60; Fig 2), the Coffeeae alliance (node 51; Fig 2), and the two tribes Steenisieae and Retiniphylleae. The combined analysis place Sabiceeae and Mussaendeae as sister groups and together they are placed sister to the corresponding large clade with the Vanguerieae alliance (node 59; Fig 4), Coffeeae alliance (node 50; Fig 4), and the two tribes Steenisieae and Retiniphylleae. The latter topology corresponds to that obtained by Kainulainen et al. [33] who also found good support for this relationship.

The endemic Seychellois genus *Glionneti*a was shown by Razafimandimbison et al. [37] to be a member of the Vanguerieae alliance. However, its position within the alliance was unclear, and the genus was resolved as sister to a large clade formed by the tribes Vanguerieae, Greeneeae, Aleisanthieae, and Ixoreae. In Kainilainen et al. [33], *Glionneti*a was left unresolved in the Vanguerieae-Greeneeae-Aleisanthieae-Ixoreae clade. In our present analyses *Glionneti*a is resolved sister to the the Vanguerieae-Greeneeae-Aleisanthieae-Ixoreae clade in the separate analysis (node 65; Fig 2), and sister to Vanguerieae in the combined analysis (node 66; Fig 4). However, neither placement is well supported and the position of *Glionneti*a in the Vanguerieae alliance remain unclear.

In the Rubioideae, the situation is slightly more complex and the relationships among early diverging lineages, as indicated here, differ compared to analyses recently published. Rydin et al. [34], for example, included a broad sample of Rubioideae taxa in their analyses using six different loci, and using bayesian inference they resolved and supported the tribe Colletoecemateae (*Colletoecema*) as sister to all remaining Rubioideae. A corresponding placement was also indicated by Robbrecht & Manen [109], and by Rydin et al. [41]. Our analysis resolve Urophylleae (*Amphidasya*) and a group including Colletoecemateae (*Colletoecema*) and Lasiantheae (*Lasianthus*) as successive sister groups to remaining Rubioideae, and the sister group relationship between Colletoecemateae and Lasiantheae is well supported. This same result is seen both in the separate (Fig 2) and in the combined analysis (Fig 4). These differences will of course affect the specific age estimates for the Rubioideae taxa involved, but will most likely have limited effects on the age estimates for more derived groups, e.g., in the Spermacoceae and Psychotrieae alliances. It is notable that a similar placement for Colletoecemateae as obtained here was also reported by Manns et al. [17]. In their analyses, as in the analyses presented here, relationships and divergence times were coestimated using relaxed-clock models implemented in BEAST, wheras Rydin et al. [41, 110] only estimated relationships using nonclock models implemented in MrBayes 3.1. This indicates that the alternative placements may result from different assumptions included in these different models. Reanalysis of the data presented here using nonclock models under MrBayes 3.2.1 (results not included) support this idea and this analysis resolved Colletoecemateae as sister to remaining Rubioideae, and not as sister to Lasiantheae. Relaxed-clock models have been suggested to be more accurate and more precise at estimating phylogenetic relationships than current nonclock methods [111], but this idea was not supported by the simulations conducted by Wertheim et al. [112], and nor by real data analyzed by Ronquist et al. [113].

### Age estimates

#### Asterids

A clear and general pattern in our results is that we resolve major groups and orders of the asterids as being of younger age than indicated by Bremer et al. [26]. Their point estimates for these groups (Table 3) are indicated by light blue dots in (Figs 1 and 3), and they either indicate ages in the lower (older) part of the error bounds obtained in the present analyses, or in some cases ages older than that. This general pattern of younger ages is seen both in our separate analysis of the asterids (Fig 1), which in terms of data and taxon sample completely mirrors that of Bremer et al. [26], and in our combined analysis (Fig 3) where the taxon sample of the Rubiaceae was extended by an additional 67 taxa. The fossil-based age constraints enforced in the present analyses differ to some extent compared to the analysis by Bremer et al. [26], but this does not explain our different results. Instead, the differences we see are primarily the result of two differences in our analytical protocols. The first is that Bremer et al. [26] employed semiparametric rate smoothing using penalized likelihood [30]. This type of analysis assumes that rates are autocorrelated, and rate changes are smoothed such that they occur more slowly and over a larger part of the tree. This assumption, together with the six minimum age constraints that they enforced in their analysis, led to a result where the entire backbone of the asterids was resolved already in the early Cretaceous [26]. In contrast, we adopted a model averaging approach, as outlined by Li & Drummond [58], and our rates were averaged across the lognormal and the exponential rate models implemented in BEAST v.1.7.5 [53]. Both are relaxed-clock models and neither makes an a priori assumption of rate autocorrelation. Instead, rates on each branch of the tree are drawn independently from the underlying rate distribution of the model [111], and modeling rates in this way allow for more drastic rate variation among adjacent branches. One effect of this that we see in our results is that old age constraints in one part of the tree, such as the minimum age constraints in the Ericales (Pentaphyllaceae; 90 Myr) and in the Cornales (90 Myr), have a much less global effect on the estimated ages, and the entire backbone of the asterids is not pushed into the early Cretaceous as in the analysis by Bremer et al. [26].

A number of methods have been proposed to relax the molecular clock. This includes local clock methods [114], smoothing methods [30, 115], and bayesian methods [111, 116–120], and each of these usually include a different set of assumptions concerning the way in which evolutionary rates may change over time. Given that evolutionary rates are largely determined by the biological systems that they affect, and that the biological systems themselves diverge during the course of evolution, one could argue that closely related lineages should have more similar rates than more distantly related lineages [31]. The evolutionary rates are said to be autocorrelated [121–122], and an assumption of autocorrelated rates is included in the smoothing methods introduced by Sanderson [30, 115], as well as in many of the bayesian relaxed-clock models that have been proposed [116–120]. However, available evidence do not support that evolutionary rates are autocorrelated. Although LePage et al. [123] found that autocorrelated models outperformed uncorrelated models on all three datasets they investigated, Drummond et al. [111] found no significant rate autocorrelation in the three large datasets that they investigated, and nor did Linder et al. [124] in their investigations of three empirical datasets. Ronquist et al. [113] reported a somewhat mixed pattern. A few major rate changes close to the root node of their hymenopteran tree caused major differences in the performance of different models, and they reported that the root part of their tree was best modeled by an uncorrelated rate model whereas other parts of their tree was better modeled by autocorrelated models [113]. Given the biological arguments behind the presence of autocorrelated rates, one would expect the degree of autocorrelation to decrease as the taxon sample becomes more sparse [31]. If correct, there would be little reason to expect any appreciable autocorrelation of rates in data sets of distantly related taxa, a pattern consistent with the results reported by Ronquist et al. [113]. The analyses presented here, that all use uncorrelated rate models (Figs 1 and 3), as well as the analyses by Bremer et al. [26], where autocorrelation was assumed, resolve most major groups and orders of the asterids with comparatively long stem lineages. This indicates that they have persisted for a considerable time, without leaving a representation in our sample of taxa, and if anything, this pattern would seem to support the use of uncorrelated models, and not models with an a priori assumption of rate autocorrelation.

A second major difference in the analytical protocols used here and by Bremer et al. [26], and that can help explain the differences in our results, concern the root node calibration. Bremer et al. [26] conducted six consecutive analyses. Each used different fossil-based information for calibrating the tree, and they thereby obtained six different crown node age estimates for the asterids. In their final analysis they used the mean value of these estimates (128 Myr) as a fixed age for the asterid crown node while including their six fossils as minimum age constraints [26]. In our present analysis, a uniform age prior was defined specifying a minimum age of 90 Myr and a maximum age of 128 Myr for the root node of our tree. The maximum age here is based on the so far complete absence of tricolpate pollen from sediment older than the Late Barremian (see fossil data under material and methods). An important difference compared to Bremer et al. [26] is that our root node concerns a larger group than the asterids, also including representatives from the rosids (*Viti*s *an*d *Dipentodon*) and the Saxifragales (*Paeonia*). A consequence of this is that the asterids must become younger, and the asterid crown group was also resolved as 121–128 Myr (separate) and 122–128 Myr (combined) in our analyses. This root node difference affects the entire tree and contributes to some of the differences we see in the age estimates of larger groups and orders of the asterids (Table 3). One could probably argue that 128 Myr is an unreasonably old age for the asterid crown group. However, specifying upper age limits in analyses such as these will always be complicated, and no matter how good the fossils record is, it can only provide soft bounds on the maximum ages in our trees [64, 125, 126]. What we can say though, is that a 128 Myr age estimate for the crown group of the asterids is older than reported by other analyses. Wikström et al. [28, 29], for example, reported their age as 107–117 Myr, Soltis et al. [127] indicated 96–108 Myr and Bell et al. [103] resolved the group as 98–111 Myr or 101–119 Myr, depending on how rate changes were modeled. Moore et al. [128] reported 125 ± 6 Myr for the eudicot crown group, and although no specific age for the asterids was given, this eudicot crown group age leaves very little room for an asterid age of 128 Myr.

#### Gentianales

The analyses yield confiicting results with respect to the age of the Gentianales crown group. In the separate analysis of the asterids, the Gentianales crown group was resolved as 75 Myr and with a 95% HPD interval of 60–91 Myr (Fig 1; Table 3). Like the other estimates of major groups and orders of the asterids, this is younger than the age reported by Bremer et al. [26] and as discussed above, there are two main reasons behind these younger estimates. Two additional analyses provide estimates for the Gentianales crown group. Results from these two analysis are highly congruent, and both indicate that the Gentianales crown group is older. The first is the separate analysis of the Gentianales (Fig 2; Table 2). This analysis is in many ways similar to a previous analyses by Bremer & Eriksson [25], and as in their analysis, a secondary root calibration point was applied to the Gentianales crown group. They based their root calibration point on the Gentianales crown group age estimate from Bremer et al. [26], but they also took other age estimates into account [27–29], and applied a normally distributed prior with a mean of 78 Myr and a standard deviation of 10 Myr on their root node. This corresponds to a 95% confidence interval between 58–98 Myr. In the analyses presented here, the secondary root calibration point was based on the Gentianales crown group age estimate from our own analysis of the asterids, which was 60–91 Myr. The analysis included 72 Gentianales taxa, 68 of which where from the Rubiaceae, and the secondary calibration point was included in the form of a normaly distributed age prior on the root node with parameters set to reflect the 95% HPD interval from the asterid analysis. Three additional fossil-based age priors, each from one of the three different subfamilies of the Rubiaceae, were also included in the analysis (see fossil data under material and methods). The analysis resolved the Gentianales crown group as 95 Myr with a 95% HPD interval of 84–106 Myr. Resolving the Gentianales as old as this may seem strange considering the age constraint applied to the root node prior to the analysis, but corresponding behavior in the posterior was seen also in the analysis of Bremer & Eriksson [25]. Our interpretation is that the fossil-based age priors in each of the three subfamilies of the Rubiaceae, together with the inferred changes in rates across the tree, force the Gentianales to become older than considered prior to the analysis.

The second analysis indicating an older age for the Gentianales crown group is the combined analysis where all the taxa were analyzed simultaneously. Results from this analysis (Fig 4, blue error bars; Table 3) are highly congruent with those obtained in the separate analyses of the Gentianales (Fig 4, red error bars; Table 3). The principal difference is that the combined analysis yields smaller error bounds. The Gentianales crown group is in the combined analysis resolved as 96 Myr but now with a 95 HPD interval of 86–104 Myr.

#### Rubiaceae

Age estimates for major groups of the Rubiaceae are given in Table 4, and they are also compared to estimates reported previously by Bremer & Eriksson [25], Antonelli et al. [16], and Manns et al. [17]. The 78 Myr Gentianales crown group age estimate reported by Bremer et al. [26] has been treated in highly inconsistent ways by previous divergence time analyses in the Rubiaceae. These different treatments can actually explain much of the differences we see in the age estimates reported by these previous analyses, and that we also see when comparing the age estimates presented here with the ones from these analyses (Table 4). Antonelli et al. [16], for example, used the estimate from Bremer et al. [26] to justify a hard upper age bound of 78 Myr for their Gentianales root node. With the exception of Cinchonoideae, their age estimates for major groups of Rubiaceae are considerably younger than indicated by all the other analyses, and this is a direct consequence of treating the Gentianales crown group estimate in this way. Using the Bremer et al. [26] estimate as a hard upper age limit also results in comparatively narrow error bounds, and this too is seen in the ages reported by Antonelli et al. [16]. The calibration scheme chosen by Antonelli et al. [16] clearly have a drastic influence on the outcome of their molecular dating analysis, but treating the age estimates reported by Bremer et al. [26] as errorless numbers in this way can not be justified. Huang et al. [22] and Nie et al. [23] also treated the Gentianales age estimate in much the same way, and their age estimates, as well as those reported by Antonelli et al. [16] should be viewed bearing this in mind. Manns et al. [17] tried to account for uncertainties in the ages reported by Bremer et al. [26], but they too enforced a hard upper age limit for the Gentianales crown group. This was applied as a uniform age prior on the root node in their analyses with a maximum age of 88 Myr. By accepting older ages for the Gentianales crown group they generally resolved major groups of the Rubiaceae as older compared to Antonelli et al. [16], but the narrow error bounds present in the results of Antonelli et al. [16] are to some extent also seen in their results [17].

The only previous analysis with a broad sampling of taxa across all three subfamilies is that of Bremer & Eriksson [25], and this is also the only one where the Gentianales age estimate from Bremer et al. [26] was treated in a justifiable way. Although there is considerable congruence between the age estimates reported by Bremer & Eriksson [25], and those obtained in our present analyses (Table 4), there are several indications of improvement. Error bounds, for example, are very broad in their analyses and largely correspond to those seen in our separate analysis of Gentianales. Our combined analysis show highly congruent age estimates to our separate analysis of the Gentianales, but with more narrow error bounds, and we interpret the broader error bounds as a direct result of the dependance on a secondary root calibration point. A second point of improvement concerns the tribal relationships. These were sometimes poorly supported, or even unresolved, in the analysis by Bremer & Eriksson [25], and some of their age estimates are likely incorrect or misleading for this reason. Their coffeeae alliance estimate, for example, indicates incongruence to our estimate (Table 4), but this is a direct consequence of poor support in this part of the tree in their analysis. They even reported an alternative lineage age of 31 Myr, which is more in line with the 31 Myr (23–39 Myr) obtained in our combined analysis.

Bremer & Eriksson [25] reported a comparatively young age estimate for the crown group of subfamily Cinchonoideae (Table 4), and this is primarily caused by differences in their treatment of the *Cephalanthu*s minimum age constraint. They never accepted reports of *Cephalanthu*s from the Late Eocene [68, 69] and the Oligocene [129–131], and based on the occurrence of *Cephalanthu*s *pusillu*s in the middle Miocene [132], they applied a minimum age for the *Cephalanthu*s stem lineage at 15 Myr. This affects the entire subfamily Cinchonoideae and explains the younger ages indicated for this group by them [25]. Prior to the present analyses, Else Marie Friis (pers. comm.), from The Natural History Museum in Stockholm, re-investigated one of the Late Eocene specimens from Germany [68, 69], and confirmed the determination of this specimen as *Cephalanthus*. We therefore applied a minimum age constraint of 34 Myr on the *Cephalanthu*s stem lineage, and this explains our older age estimate for the subfamily Cinchonoideae.

If we compare stem lineage ages for individual tribes obtained in the present analysis (S2 Table), with those reported by Bremer & Eriksson [25] there are more incongruences, but many of these arise for understandable reasons. Sometimes tribal delimitations have changed, and although we use the same name for the group, we actually refer to different groups. Psychotrieae, for example, also included genera such as *Geophila*, *Palicourea*, *Chassali*a in the analyses by Bremer & Eriksson [25], genera that are here recognized under the tribal name Palicoureeae [133]. Our stem lineage age for Psychotrieae and Palicoureeae together is 46 Myr (41–51 Myr) in the separate analyses, and 44 Myr (39–50 Myr) in the combined analysis, and these estimates show considerably more congruence to the Psychotrieae age reported by them [25]. A second source of incongruence relate to differences in the tribal relationships indicated by the two analysis. The tribe Coffeeae, for example, was resolved in the dating analysis by Bremer & Eriksson [25] as sister to a large group including the tribes Bertiereae, Cremasporeae, Octotropideae, Pavetteae, and Gardenieae, and their Coffeeae stem lineage age refers to the split between these groups. Here, the tribe Coffeeae is resolved sister to the tribe Bertiereae (Figs 2 and 4) and our Coffeeae stem lineage age refere to the split between these two tribes. Other incongruences can be explained by differences in the sampling density of the two analyses. The present analysis in general only included a single representative for each tribe, and this leads to a situation where we likely underestimate the stem lineage age for some of the tribes. Tribes like Steenisieae and Retiniphylleae, who has sister groups comprising larger groups of several other tribes, will likely be less affected by this problem than tribes that have sister groups compring fewer or single tribes. Examples of the latter are Pavetteae/Gardenieae, Sherbournieae/Cordiereae, Coffeeae/Bertiereae in the Coffeeae alliance, and Ixoreae/Aleisantheae in the Vanguerieae alliance, and their stem lineage ages are likely underestimated in the present analysis.

## Conclusion

Divergence times of the Rubiaceae (coffee family) are estimated, and we do this by expanding a previously published 6-gene asterid data set with a sample of 67 additional Rubiaceae taxa representing all three subfamilies recognized in the family. We present updated age estimates for major groups and tribes of the Rubiaceae, and taken together these new estimates provide a significant step forward towards the long-term goal of establishing a robust temporal framework for the divergence of the family. Previously published estimates either directly, or indirectly, relied on an earlier 78 Myr Gentianales crown group age estimate reported by Bremer et al. [26] to provide the upper age bound for the root node in their analyses [16–17, 22, 23, 25], and this reliance imposes weaknesses. They also defined the upper age bound in their analyses in highly inconsistent ways. Antonelli et al. [16], for example, used it to justify a fixation of the Gentianales crown group at 78 ± 0 Myr in their analysis. This has been followed by other workers [22–23], but treating the age estimate from Bremer et al. [26] in this way simply cannot be justified. Our analyses result in a general pattern where major groups and orders of the asterids are resolved as somewhat younger than indicated in the analysis by Bremer et al. [26]. We interpret this general pattern as primarily caused by our usage of non-autocorrelated relaxed clock models, as opposed to the smoothing methods used by Bremer et al. [26], as well as by differences in the root node calibration between our two analyses. In the Rubiaceae, our age estimates show much congruence to those previously reported by Bremer & Eriksson [25], but with more narrow error bounds. By bridging the analysis of Rubiaceae with the asterid analysis, we remove our analysis from the dependance of a secondary calibration point, and we interpret the more narrow error bounds as a result of this removal. In addition, our better supported relationships reduce some of the uncertainties displayed in their results.

## Supporting Information

S1 Text Fossil based minimum age constraints. (PDF)

S1 Table List of investigated taxa with accession numbers. (PDF)

S2 Table Estimated ages for all nodes obtained in the separate and in the combined analyses. (PDF)

S1 Dataset Data xml-file used for the seperate analysis of the asterids. (XML)

S2 Dataset Data xml-file used for the separate analysis of the Gentianales. (XML)

S3 Dataset Data xml-file used for the combined analysis. (XML)
