# Correction: A Revised Time Tree of the Asterids: Establishing a Temporal Framework For Evolutionary Studies of the Coffee Family (Rubiaceae)

**DOI:** 10.1371/journal.pone.0157206

**Published:** 2016-06-03

**Authors:** Niklas Wikström, Kent Kainulainen, Sylvain G. Razafimandimbison, Jenny E. E. Smedmark, Birgitta Bremer

S2 Table incorrectly omits a fossil time constraint. The corrected table can be viewed below.

## Supporting Information

S2 Table Estimated ages for all nodes obtained in the separate and in the combined analyses. This file includes the correct data.
(PDF)
