# Supplementary material for: A Revised Time Tree of the Asterids: Establishing a Temporal Framework For Evolutionary Studies of the Coffee Family (Rubiaceae)
Source: PLoS One. 2015 May 21;10(5):e0126690. doi: 10.1371/journal.pone.0126690 (PMC4462594; doi:10.1371/journal.pone.0126690)
Supplement: S1 Text — (PDF) [file pone.0126690.s001.pdf]

S1 Text – Fossil based minimum age constraints

Based on fossil evidence, 17 minimum age constraints were defined and enforced in our analyses, and they were all defined as uniform priors with their minimum ages based on the ages of the fossils and their maximum ages set to 128 Myr which correspond to the maximum age of our root node. Twelve of the constraints follow the minimum age estimates accepted and reported by Martínez-Millan (2010); two were based on more recently reported Asteraceae and Apocynaceae fossils, both from the Middle Eocene and that were never considered by Martínez-Millan; and three were based on Rubiaceae fossils, each representing one of the three subfamilies recognized in the family. Compared to Martínez-Millan (2010) we used a more up to date geologic time scale Gradstein et al. (2012). Details of the fossils on which they were based, their ages, from where and by whom they were documented, and their phylogenetic placements in our analyses are discussed at length below.

Cornales

The oldest cornalean fossils are *Hironoia fusiformis* from the Coniacian-Santonian (Late Cretaceous) of Kamikitaba, Japan (Takahashi et al., 2002) and *Tylerianthus crossmanensis* from the Turonian (Late Cretaceous) of New Jersey, USA (Gandolfo et al., 1998), and both taxa place the crown group of extant Cornales in the Late Cretaceous. *Hironoia fusiformis* share synapomorphies with extant Cornaceae (Bremer et al., 2004; Takahashi et al., 2002) and *Tylerianthus crossmanensis* was resolved with Hydrangeaceae by Crepet et al. (2004), indicating placements for both fossils well inside extant Cornales. Martínez-Millan (2010) accepted this and considered their first occurrences as best fossil-based minimum age estimates for the stem lineage Cornaceae (*Hironoia fusiformis*) and the stem lineage Hydrangeaceae (*Tylerianthus crossmanensis*). The same placement of *Hironoia fusiformis* was also accepted by Bremer et al. (2004) who applied a minimum age constraint for the split between Cornaceae and Grubbiaceae in their analyses at 88 Myr based on this fossil taxon. Relationships among families in extant Cornales is, however, partially unsupported (Xiang et al., 2011), and our analyses include representatives from no more than three out of 7 families commonly recognized in the group. We adopted a slightly more conservative placement of these two fossil taxa and specified a uniform prior age distribution for the crown group of extant Cornales in our analyses (node 177, Figs. 1 and 3) with a minimum age of 90 Myr (Late Cretaceous, Turonian; Gandolfo et al., 1998).

Ericales

Ericales have an extensive fossil record dating back to the Cretaceous, and fusainized flowers from Late Cretaceous deposits have been associated to various modern families of Ericales including Actinidiaceae, Diapensiaceae, Ericaceae, and Pentaphyllaceae (Crepet, 1996; Crepet et al., 2013; Friis, 1985b; Keller et al., 1996; Nixon & Crepet, 1993; Schönenberger & Friis, 2001; Schönenberger et al., 2012). The oldest of these flowers are from the Turonian (Late Cretaceous) of New Jersey, USA and include *Paleoenkianthus sayrevillensis* (Nixon & Crepet, 1993), *Pentapetalum trifasciculandricus* (Martínez-Millan et al., 2009), and three species of the genus *Raritaniflora* (Crepet et al., 2013). Either one of these fossils provide a good fossil-based minimum age estimate for the crown group of extant Ericales. *Paleoenkianthus sayrevillensis* was also used for this purpose by Crepet et al. (2004), but *Pentapetalum trifasciculandricus* was associated to a more narrow group of Ericales, the family Pentaphylaceae (*Pentaphylax*), in the original description by Martínez-Millan et al. (2009). Martínez-Millan (2010) accepted this placement and reported the Turonian as the best fossil-based minimum age estimate for the split between Pentaphylaceae *(Pentaphylax)* and Ternstroemiaceae. Ternstroemiaceae included a total of 3 taxa in her phylogeny including *Ternstroemia*, *Clayera*, and *Eurya* (Martínez-Millan, 2010). Fossil seeds, described from the Santonian (Late Cretaceous) of Europe (Knobloch & Mai, 1986), and that share synapomorphies with the extant genus *Eurya* (Pentaphylaceae), lend additional support for such an early origin of extant Pentaphylaceae. These seeds were also used by Bremer et al. (2004) to specify 86 Myr as a minimum age constraint for Pentaphylaceae in their analyses. We specified a prior age distribution for the crown group of extant Pentaphylaceae (node 164, Figs. 1 and 3) in our analyses with a minimum age of 90 Myr (Late Cretaceous, Turonian; Martínez-Millan et al., 2009).

A second fossil that have been placed in a more narrow group of Ericales is *Parasaurauia allonensis*. This fossil taxon was described by Keller et al. (1996) from the Campanian (Late Cretacous) of Georgia, USA. Their phylogenetic analyses placed it in Actinidiaceae, sister to extant genera of *Saurauia* and

Actinidia (Keller et al., 1996), and Martínez-Millan (2010) accepted the Campanian as the best fossil-based minimum age estimate for stem lineage Actinidiaceae. A Late Cretacous origin for Actinidiaceae is also indicated by seeds described from the Turonian of Central Europe (Knobloch & Mai, 1986). These seeds have been included in the extant genus *Saurauia*, and their first occurrence was used by Bremer et al. (2004) as a minimum age constraint for stem lineage Actinidiaceae. If correctly placed, they indicate an older origin for Actinidiaceae than *Parasaurauia allonensis.* However, they failed to pass the filtering criteria used by Martínez-Millan (2010). We followed her and specified a uniform prior age distribution for the Actinidiaceae stem lineage (node 169, Fig. 1; node 168, Fig. 3) in our analyses with a minimum age of 72 Myr (Late Cretaceous, Campanian; Keller et al., 1996).

Aquifoliales

Knobloch & Mai (1986) described fruits of Aquifoliaceae from the Maastrichtian (Late Cretaceous) of Germany. These fruit fossils were accepted by Bremer et al. (2004) who enforced a minimum age constraint for the crown group of extant Aquifoliales at 70 Myr. They did, however, not pass the filtering criteria used by Martínez-Millan (2010). She accepted the occurrence of the seed fossil *Ilex hercynica* from Germany as the currently best fossil-based minimum age estimate of Aquifoliales. This seed fossil was reported from Gonna (Abschnitt II), Sangerhausen, Sachsen-Anhalt, Germany by Mai (1987) and was dated as Early Paleocene. Martínez-Millan (2010) grouped this fossil with *Ilex,* Aquifoliaceae, and we specified a uniform prior age distribution for the *Ilex* stem lineage in our analyses (node 152, Figs. 1 and 3) with a minimum age of 62 Myr (Early Paleocene; Mai, 1987).

Apiales

Apiales are represented in the fossil record by a large number of fossil fruits and endocarps. Some of the earliest records are those of Knobloch & Mai (1986) who reported endocarps of modern genera *Aralia*, *Acanthopanax*, and *Schefflera* from the Maastrichtian (Late Cretaceous) of Germany. These were accepted and used by Bremer et al. (2004) who constrained a group including Araliaceae, Apiaceae, and Pittosporaceae at 70 Myr. Also these fruit fossils failed to pass the filtering criteria used by Martínez-Millan (2010). Instead, the occurrence of leaf fossils of *Dendropanax* from the Middle Eocene of Tennessee, USA (Dilcher & Dolph, 1970) was accepted by her as one of the best fossil-based minimum age estimate of Apiales. She grouped *Dendropanax* with Araliaceae/Apiaceae, sister to Pittosporaceae. Following this placement we specified a uniform prior age distribution for the Apiaceae/Araliaceae stem lineage (node 139, Figs. 1 and 3) in our analyses with a minimum age of 38 Myr (Middle Eocene; Dilcher & Dolph, 1970).

The fruit fossil *Toricellia bonesii,* reported from the Middle Eocene of USA and Germany (Manchester, 1994, 1999), were also used by Martínez-Millan (2010) to support a minimum age of Middle Eocene for Apiales, and she grouped this fruit fossil with the *Toricellia* group (sensu Chandler & Plunkett, 2004). More recently Manchester et al. (2009) documented fruits of *Toricellia bonensii* from the Late Paleocene of North Dakota, USA, pushing this age estimate further back in time. Based on this more recent report, we specified a uniform prior age distribution for the *Toricellia* group stem lineage (node 137, Figs. 1 and 3) in our anaylses with a minimum age of 56 Myr (Late Paleocene; Manchester et al., 2009).

Dipsacales

The oldest currently accepted occurrence of the Dipsacales in the fossil record is *Diplodipelta reniptera* from the Late Eocene of eastern North America (Manchester & Donoghue, 1995), and this fossil was also used as a calibration point by Bell & Donoghue (2005) in their attempt at calibrating the Dipsacales. Several synapomorphies place *Diplodipelta* well inside Dipsacales, in the family Linneaceae (Manchester & Donoghue, 1995), and this placement was followed by Martínez-Millan (2010) who accepted it as a minimum age estimate for the split between Linneaceae and a group of three other families: Morinaceae, Dipsaceae, and Valerianaceae. We have followed her and specified a uniform prior age distribution for the crown group of all four families (node 147, Figs. 1 and 3) in our analyses with a minimum age of 34 Myr (Late Eocene; Manchester & Donoghue, 1995).

Asterales

The early fossil record of Asterales primarily comprises pollen records of the three families Goodeniaceae, Menyanthaceae, and Asteraceae (Barreda et al., 2010a; Martínez-Millan, 2010). Reviewing the occurrences and geographical distributions of these fossil pollen Barreda et al. (2010a) showed that all three families were well differentiated by the Oligocene and that they also had established a broad southern hemisphere distribution by this time. Taken together this was seen to support an Eocene, or earlier origin of the group (Barreda et al., 2010a). A corresponding origin of Asterales was also indicated by Martínez-Millan (2010). She considered *Tubulifloridites viteauensis,* a fossil pollen taxon reported from the Bartonian (Middle Eocene) of Namibia, Africa by Zavada & de Villiers (2000) as the best fossil-based minumum age estimate for the group, and she placed the Asteraceae stem lineage in the Bartonian based on this taxon. More recently a capitulescence of Asteraceae was recovered from Middle Eocene deposits of northwestern Patagonia (Barreda et al., 2010b, 2012). This fossil was described as *Raiguenrayun cura* and based on characters seen in the associated pollen grains the authors placed the taxon in the crown group of extant Asteraceae (Barreda et al., 2012). The age of the deposits were constrained by radiometric dating at 47.5 ± 0.05 Myr (Wilf et al., 2005), and based on this fossil taxon we specified a uniform prior age distribution for the Asteraceae stem lineage (node 132, Figs. 1 and 3) in our analyses with a minimum age of 47 Myr (Barreda et al., 2012).

Gentianales

First occurrences of *Emmenopterys dilcherii* (Rubiaceae), a fruit fossil from the Lutetian (Middle Eocene) of Oregon, USA (Manchester, 1994), and *Apocynospermum rostratum* (Apocynaceae), a seed fossil described from the Early–Middle Oligocene of Bembridge, England (Reid & Chandler, 1926) were indicated by Martínez-Millan (2010) as the currently best minimum age estimates for stem lineages Rubiaceae and Apocynaceae. In addition to *Apocynospermum rostratum* Reid & Chandler (1926) also described *A. striatum* from the same locality, and this seed fossil was also accepted by Martínez-Millan (2010) as Apocynaceae. More recently, Collinson et al. (2012) reported corresponding seed fossil from the Middle Eocene Messel Formation near Darmstadt, Germany. The upper parts of this Formation was recently dated to at least 47 Myr (Lenz et al., 2014). Following Collinson et al. (2012) and Lenz et al. (2014), we specified a uniform prior age distribution for stem lineage Apocynaceae (node 111, Figs. 1 and 3) in our analyses with a minimum age of 47 Myr. For Rubiaceae, no stem lineage prior was specified. Here, information from younger fossils nested well inside each of the three subfamilies were used instead. Fossils used were pollen of *Scyphiphora* (Ixoroideae) from the Miocene of the Eniwetok Atoll, Marchall Islands (Leopold, 1969), *Cephalanthus pusillus* (Cinchonoideae), a fruit fossil from the Late Eocene of the Weißelster Basin, Sachsen, Germany (Mai & Walther, 2000), and *Morinda chinensis* (Rubioideae), a fruit fossil from the Middle Eocene, Changchang Basin of Hainan Island, south China (Shi et al., 2012).

The *Scyphiphora* pollen was originally described by Leopold (1969) who reported it from different layers of Miocene age. Later Saenger (1998) discussed the origins of modern mangrove vegetation from an evolutionary perspective and gave two age estimates for the *Scyphiphora* pollen: ~16 Myr for Japanese specimens and ~23 Myr for the Marshall Islands specimens originally reported by Leopold (1969). Following Saenger (1998), several divergence time analyses in Rubiaceae have accepted the older estimate and applied a 23 Myr fossil-based constraint for the *Scyphiphora* stem lineage (Bremer & Eriksson, 2009; Huang et al., 2013; Manns et al., 2012; Nie et al., 2013). However, the original report by Leopold (1969) lend no support for this and only indicate an Early Miocene (16–23 Myr) age for the *Scyphiphora* pollen. We specified a uniform prior age distribution for the *Scyphiphora* stem lineage (node 62, Figs. 2 and 4) in our analyses with a minimum age of 16 Myr (Early Miocene; Leopold, 1969).

Fossil fruits of *Cephalanthus* have been described from the Eocene (Mai & Walther, 1985, 2000), from the Oligocene (Mai & Walther, 1978; Mai, 1997; Dorofeev, 1963), and from the Miocene (Dorofeev, 1960, 1963; Friis, 1985a; Mai, 2001; Raniecka-Bobowska, 1959; Szafer, 1961) at a large number of localities across central Europe and Russia. The oldest localities are from The Weißelster Basin floras of Germany and are of Late Eocene age (Mai & Walther, 2000; Collinson et al., 2009; Kvaček, 2010). The fruits are characterized as schizocarps with two, one-seeded, obovoid and slightly dorsiventrally flattened mericarps, they have seeds that are strophiolate, and they are taxonomically very distinct. One of the Late Eocene specimens from Germany (Mai & Walther, 1985, 2000) was recently reinvestigated by Else Marie Friis (The Natural History Museum, Stockholm) and she confirmed the determination of

this specimen as *Cephalanthus pusillus* (E. M. Friis, personal communication). Based on this specimen we specified a uniform prior age distribution for the *Cephalanthus* stem lineage (node 79, Figs. 2 and 4) in our analyses with a minimum age of 34 Myr (Late Eocene; Mai & Walther, 2000).

Morinda chinensis, originally described by Shi et al. (2012), is a well preserved mulberry-shaped infructescence of syncarpous fruits, a type of syncarp seen among extant members of the genus *Morinda*. Razafimandimbison et al. (2009) investigated the occurrences and evolution of such syncarps in the tribe Morindeae and showed that they are evolutionary labile in the group and not diagnostic for the genus *Morinda* (Razafimandimbison et al., 2009). They are, however, characteristic for members of the Morindeae tribe and their occurrence in the Middle Eocene of south China provides a good fossil-based minimum age estimate for the stem lineage of extant Morindeae. We specified a uniform prior age distribution for the Morindeae stem lineage (node 106, Figs. 2 and 4) in our analyses with a minimum age of 38 Myr (early Late Eocene; Shi et al., 2012).

Solanales

Cantisolanum daturoides, a seed fossil described from the Early Eocene (Ypresian) of England, has been considered the first evidence of Solanaceae in the fossil record (Collinson et al., 1993). However, it was rejected as a Solanaceae fossil by Martínez-Millan (2010) who reported the first occurrence of *Solanispermum reniforme* as the best fossil-based minimum age estimate for stem lineage Solanaceae. This seed fossil is also from the Eocene of Southern England (Chandler, 1962, 1963), but reported from deposits of somewhat younger age then the London Clay Formation from where *Cantisolanum daturoides* was originally described (Reid & Chandler, 1926). Recently, Särkinen et al. (2013) conducted a review of the Solanaceae fossil record. They also reinvestigated the specimens of *Cantisolanum daturoides* and *Solanispermum reniforme* using X-ray computed tomography and based on these investigations, they too rejected *Cantiosolanum daturoides* as a Solanaceae fossil. They agreed with Martínez-Millan (2010) and considered the first occurrence of *Solanispermum reniforme* as the best fossil-based minimum age estimate of Solanaceae (Särkinen et al., 2013). The first occurrence of *Solanispermum reniforme* is from the Poole Formation, Bracklesham Group in Dorset, UK (Chandler, 1962), and following King (2006) this Formation spans from late Ypressian to early Lutetian in age. We specified a uniform prior age distribution for the Solanaceae stem lineage (node 14, Figs. 1 and 3) in our analyses with a minimum age of 46 Myr (Poole Formation, early Lutetian; Chandler, 1962; King, 2006).

Lamiales

The fossil record of Lamiales is meager but three fossil taxa from the Bembridge Flora, UK were used by Martínez-Millan (2010) for obtaining minimum age estimates in the group. Fossil taxa that she used were *Acanthus rugatus* (Acanthaceae), *Radermachera pulchra* (Bignoniaceae), and *Melissa parva* (Lamiaceae). The first two are seed fossils and *Melissa parva* is a fruit fossil, and all three were described from the Early–Middle Oligocene of Bembridge, England by Reid & Chandler (1926). Recent updates place the Bembridge Flora in the Late Eocene (Collinson et al., 2009; Hooker et al., 2007, 2009). Based on the occurrence of these fossils we specified uniform prior age distributions for the Acanthaceae (node 29, Figs. 1 and 3), the Bignoniaceae (node 33, Figs. 1 and 3), and the Lamiaceae (node 29, Fig. 1; node 31, Fig. 3) stem lineages in our analyses with minimum ages of 34 Myr (Late Eocene; Collinson et al., 2009; Hooker et al., 2007, 2009; Reid & Chandler, 1926).

Vahliaceae

Friis & Skarby (1982) described *Scandianthus costatus* and *S. major* from the Late Santonian–Early Campanian flora of Åsen, southern Sweden. They were originally associated with members of the families Saxifragaceae, Hydrangeaceae, Vahliaceae, and Escalloniaceae (Friis & Skarby, 1982; Friis, 1984). At the time, these families were considered closely related and part of the Saxifragales complex. Subsequent analyses have, however, placed these familes in widely seperated groups with Saxifragaceae in the Saxifragales (rosids), Hydrangeaceae in the Cornales (asterids), Vahliaceae in the lamiid group of the asterids, and Escalloniaceae in the campanuliid group of the asterids (see Soltis et al., 2011). To clarify their relationships Martínez-Millan (2010) compiled the original data from Friis & Skarby (1982) and conducted a phylogenetic analyses of these fossils. Their analysis resolved *Scandianthus* as sister to Vahliaceae, and following this result they accepted Late Santonian as the best fossil-based minimum age estimate for the stem lineage Vahliaceae (Martínez-Millan, 2010). We specified a uniform prior age

distribution for the Vahliaceae stem lineage (node 9, Figs. 1 and 3) in our analyses with a minimum age of 84 Myr (Late Santonian; Friis & Skarby, 1982).

References

Barreda V, Palazzesi L, Katinas L, Crisci JV, Tellería MC, Bremer K, G PM, Bechis F, Corsolini R (2012) An extinct Eocene taxon of the daisy family (Asteraceae): evolutionary, ecological and biogeographical implications. Ann Bot 109: 127–134.

Barreda V, Palazzesi L, Tellería MC, Katinas L, Crisci JV (2010a) Fossil pollen indicates an explosive radiation of basal Asteraceae lineages and allied families during Oligocene and Miocene times in the Southern Hemisphere. Rev Palaeobot Palynol 160: 102–110.

Barreda V, Palazzesi L, Tellería MC, Katinas L, Crisci JV, Bremer K, G PM, Corsolini R, Rodríguez Brizuela R, Bechis F (2010b) Eocene Patagonia fossils of the daisy family. Science 329: 1621–1621.

Bell CD, Donoghue MJ (2005) Dating the Dipsacales: comparing models, genes, and evolutionary implications. Am J Bot 92: 284–296.

Bremer B, Eriksson T (2009) Timetree of Rubiaceae – phylogeny and dating the family, subfamilies and tribes. Int J Plant Sci 170: 766–793.

Bremer K, Friis EM, Bremer B (2004) Molecular phylogenetic dating of asterid flowering plants shows Early Cretaceous diversification. Syst Biol 53: 496–505.

Chandler GT, Plunkett GM (2004) Evolution in Apiales: nuclear and chloroplast markers together in (almost) perfect harmony. Bot J Linn Soc 144: 123–147.

Chandler MEJ (1962) The Lower Tertiary Floras of Southern England II. Flora of the Pipe-clay Series of Dorset (Lower Bagshot). London: Brittish Museum (Natural History).

Chandler MEJ (1963) The Lower Tertiary Floras of Southern England III. Flora of the Bournemouth Beds; The Boscombe, and the Highcliff Sands. London: Brittish Museum (Natural History).

Collinson ME, Boulter MC, Holmes PL (1993) Magnoliophyta ('Angiospermae'). In: Benton MJ, editor, The fossil record 2, London: Chapman & Hall, chapter 45. pp. 809–841.

Collinson ME, Manchester SR, Wilde V (2012) Fossil fruits and seeds of the Middle Eocene Messel biota, Germany. Abh Senckenberg Ges Naturforsch 570: 1–251.

Collinson ME, Manchester SR, Wilde V, Hayes P (2009) Fruit and seed floras from exceptionally preserved biotas in the European Paleogene. Bull Geosci 85: 155–162.

Crepet WL (1996) Timing in the evolution of derived floral characters: Upper Cretaceous (Turonian) taxa with tricolpate and tricolpate-derived pollen. Rev Palaeobot Palynol 90: 339–359. In angio palaeo box.

Crepet WL, Nixon KC, Daghlian CP (2013) Fossil Ericales from the Upper Cretaceous of New Jersey. Int J Plant Sci 174: 572–584.

Crepet WL, Nixon KC, Gandolfo MA (2004) Fossil evidence and phylogeny: The age of major angiosperm clades based on mesofossil and macrofossil evidence from Cretaceous deposits. Am J Bot 91: 1666–1682.

Dilcher DL, Dolph GE (1970) Fossil leaves of *Dendropanax* from Eocene sediments of southeastern North America. Am J Bot 57: 153–160.

Dorofeev PI (1960) Novye dannye o tretcnych florach kireevskogo jara nar obi. Dokl Akad Nauk SSR 133: 211–213.

Dorofeev PI (1963) Tretichnye Flory Zapadnoi Sibiri. Moscow: Izvestiya Akademii Nauk SSR.

Friis EM (1984) Preliminary report of Upper Cretaceous angiosperm reproductive organs from Sweden and their level of organization. Ann Mo Bot Gard 71: 403–418.

Friis EM (1985a) Angiosperm fruits and seed from the middle Miocene of Jutland Denmark. K Dan Vidensk Selsk Skr 24: 1–165.

Friis EM (1985b) *Actinocalyx* gen. nov. sympetalous angiosperm flowers from the Upper Cretaceous of southern sweden. Rev Palaeobot Palynol 45: 171–183.

Friis EM, Skarby A (1982) *Scandianthus* gen. nov., angiosperm flowers of saxifragalean affinity from the upper cretaceous of southern sweden. Ann Bot 50: 569–583.

Gandolfo MA, Nixon KC, Crepet WL (1998) *Tylerianthus crossmanensis* gen. et sp. nov. (aff. Hydrangeaceae) from the Upper Cretaceous of New Jersey. Am J Bot 85: 376–386.

Gradstein FM, Ogg JG, Schmitz MD, Ogg GM, editors (2012) The geologic time scale 2012. Oxford, UK: Elsevier.

Hooker JJ, Collinson ME, Grimes ST, Sille NP, Mattey DP (2007) Discussion on the Eocene-Oligocene boundary in the UK. J Geol Soc London 164: 685–688.

Hooker JJ, Grimes ST, Mattey DP, Collinson ME, Sheldon ND (2009) Refined correlation of the UK late Eocene – Early Oligocene Solent Group and timing of its climate history. Geol Soc Spec Pap 452: 179–195.

Huang WP, Sun H, Deng SG T Razafimandimbison, Nie ZL, Wen J (2013) Molecular phylogenetics and biogeography of the eastern Asian–eastern North American disjunct *Mitchella* and its close relative *Damnacanthus* (Rubiaceae, Mitchelleae). Bot J Linn Soc 171: 395–412.

Keller JA, Herendeen PS, Crane PR (1996) Fossil flowers of the Actinidiaceae from the Campanian (Late Cretaceous) of Georgia. Am J Bot 83: 528–541.

King C (2006) Paleogene and Neogene: uplift and a cooling climate. In: Brenchley PJ, Rawson PF, editors, The Geology of England and Wales, London: The Geological Society, chapter 16. 2nd edition, pp. 395–428.

Knobloch E, Mai DH (1986) Monographie der früchte und samen in der kreide von mitteleuropa. Rozpr Ustred Ustavu Geol 47: 1–219.

Kvaček Z (2010) Forest flora and vegetation of the European Early Palaeogene – a review. Bull Geosci 85: 63–76.

Lenz OK, Wilde V, Mertz DF, Riegel W (2014) New palynology-based astronomical and revised ^{40}Ar/^{39}Ar ages for the Eocene maar lake of Messel (Germany). Int J Earth Sci (Geol Rundsch) DOI 10.1007/s00531-014-1126-2: 1–17.

Leopold EB (1969) Miocene pollen and spore flora of Eniwetok Atoll, Marchall Islands. US Geol Surv Prof Paper 260-II: 1133–1185.

Mai DH (1987) Neue früchte und samen aus Paläozänen ablagerungen mitteleuropas. Feddes Repert 98: 105–126.

Mai DH (1997) Die oberoligozänen floren am Nordrand der Sachsischen Lausitz. Palaeontographica Abt B 244: 1–124.

Mai DH (2001) Die mittelmiozänen und obermiozänen floren aus der Meuroer und Raunoer Folge in der Lausitz. iii. Fündstellen und palaeobiologie. Palaeontographica Abt B 258: 1–85.

Mai DH, Walther H (1978) Die floren der Haselbacher Serie im Weißelster-Becken. Abh Stat Mus Miner Geol Dresden 28: 1–200.

Mai DH, Walther H (1985) Die obereozänen floren des Weißelster-Beckens und seiner Randgebeite. Abh Stat Mus Miner Geol Dresden 33: 1–260.

Mai DH, Walther H (2000) Die fundstellen eozäner floren des Weißelster-Beckens und seiner Randgebiete. Altenb Naturwiss Forsch 13: 1–59.

Manchester SR (1994) Fruits and seeds of the Middle Eocene Nut Beds flora, Clarno formation, north central Oregon. Palaeontographica Americana 58: 1–205.

Manchester SR (1999) Biogeographical relationships of North American Tertiary floras. Ann Mo Bot Gard 86: 472–522.

Manchester SR, Chen ZD, Lu AM, Uemura K (2009) Eastern Asian endemic seed plant genera and their paleogeographic history throughout the northern hemisphere. J Syst Evol 47: 1–42.

Manchester SR, Donoghue MJ (1995) Winged fruits of Linnaeeae (Caprifoliaceae) in the tertiary of western north america: *Diplodipelta* gen. nov. Int J Plant Sci 156: 709–722. In angio palaeo box.

Manns U, Wikström N, Taylor CM, Bremer B (2012) Historical biogeography of the predominantly neotropical subfamily Cinchonoideae (Rubiaceae): Into or out of America? Int J Plant Sci 173: 261–289.

Martínez-Millan M (2010) Fossil record and age of the Asteridae. Bot Rev 76: 83–135.

Martínez-Millan M, Crepet WL, Nixon KC (2009) *Pentapetalum trifasciculandricus* gen. et sp. nov., a thealean fossil flower from the Raritan Formation, New Jersey, USA (Turonian, Late Cretaceous). Am J Bot 96: 933–949.

Nie ZL, Deng T, Meng Y, Sun H, Wen J (2013) Post-Boreotropical dispersals explain the pantropical disjunction in *Paederia* (Rubiaceae). Ann Bot 111: 873–886.

Nixon KC, Crepet WL (1993) Late cretaceous fossil flowers of ericalean affinity. Am J Bot 80: 616–623.

Raniecka-Bobowska J (1959) Trzeciorzedowa flora nasienna z Konina. Biul Inst Geol 130: 159–252.

Razafimandimbison SG, McDowell TD, Halford DA, Bremer B (2009) Molecular phylogenetics and generic assessment in the tribe Morindeae (Rubiaceae-Rubioideae): how to circumscribe *Morinda* L. to be monophyletic? Mol Phylogenet Evol 52: 879–886.

Reid EM, Chandler MEJ (1926) Catalogue of Cainozoic plants in the Department of Geology, vol. 1. The Bembridge Flora. London, UK: British Museum (Natural History).

Saenger P (1998) Mangrove vegetation: an evolutionary perspective. Mar Freshwater Res 49: 277–286.

Schönenberger J, Friis EM (2001) Fossil flowers of ericalean affinity from the Late Cretaceous of Southern Sweden. Am J Bot 88: 467–480.

Schönenberger J, von Balthazar M, Takahashi M, Xiao X, Crane PR, Herendeen PS (2012) *Glandulocalyx upatoiensis*, a fossil flower of Ericales (Actinidiaceae/Clethraceae) from the Late Cretaceous (Santonian) of Georgia, USA. Ann Bot 109: 921–936.

Shi XG, Jin JH, Ye CX, Liu WQ (2012) First fruit fossil record of *Morinda* (Rubiaceae) from China. Rev Palaeobot Palynol 179: 13–16.

Soltis DE, Smith SA, Cellinese N, Wurdack KJ, Tank DC, Brockington SF, F RRN, Walker JB, Moore MJ, Carlsward BS, Bell CD, Latvis M, Crawley S, Black C, Diouf D, Xi Z, Rushworth CA, Gitzendanner MA, Sytsma KJ, Qiu YL, Hilu KW, Davis CC, Sanderson MJ, Beaman RS, Olmstead RG, Judd WS, Donoghue MJ, Soltis PS (2011) Angiosperm phylogeny: 17 genes, 640 taxa. Am J Bot 98: 704–730.

Szafer W (1961) Miocenska flora ze starych gliwic na slasku. Prace Instytut Geologiczny 33: 1–205.

Särkinen T, Bohs L, G OR, Knapp S (2013) A phylogenetic framework for evolutionary study of the nightshades (Solanaceae): a dated 1000-tip tree. BMC Evol Biol 13: 214.

Takahashi M, Crane PR, Manchester SR (2002) *Hironoia fusiformis* gen. et sp. nov.: A cornalean fruit from the Kamikitaba locality (Upper Cretaceous, Lower Coniaceae) in northeastern Japan. J Plant Res 115: 463–473.

Wilf P, Johnson KR, Cúneo NR, Smith ME, Singer BS, Gandolfo MA (2005) Eocene plant diversity at Laguna del Hunco and Río Pichileufú, Patagonia, Argentina. Am Nat 165: 634–650.

Xiang QY, Thomas DT, Xiang QP (2011) Resolving and dating the phylogeny of Cornales – Effects of taxon sampling, data partitions, and fossil calibrations. Mol Phylogenet Evol 59: 123–138.

Zavada MS, de Villiers SE (2000) Pollen of the Asteraceae from the Paleocene–Eocene of South Africa. Grana 39: 39–45.
