# Supplementary material for: A Revised Time Tree of the Asterids: Establishing a Temporal Framework For Evolutionary Studies of the Coffee Family (Rubiaceae)
Source: PLoS One. 2015 May 21;10(5):e0126690. doi: 10.1371/journal.pone.0126690 (PMC4462594; doi:10.1371/journal.pone.0126690)
Supplement: S1 Table — (PDF) [file pone.0126690.s002.pdf]

S1 Table – List of investigated taxa with accession numbers

S1 Table. List of investigated taxa with accession numbers. Voucher information is given for new sequences not previously reported and their accession numbers are indicated in bold. Standard herbarium codes follow Thiers (2008).

**Family/Group**	**Tribe**	**Genus**	**Species**	**Citation/Voucher**	***rbc*L**	***ndh*F**	***mat*K**	***trn*V**	***rps*16**	***trn*L**
Acanthaceae		*Acanthus*	*Acanthus longifolius* Host	Bremer et al. (2002)			AJ429326	AJ429679	AH431037	AJ430912
			Acanthus montanus T.Anderson	Hedrén et al. (1995)	L12592					
				Bremer et al. (2002)		AJ429115				
		Avicennia	*Avicennia marina* Vierh.	Bremer et al. (2002)		AJ429116	AJ429327	AJ429680	AJ431038	AJ430913
			Avicennia nitida Jacq.	Wagstaff & Olmstead (1997)	U28868					
Actinidiaceae		*Actinidia*	*Actinidia arguta* Miq.	Albach et al. (2001a,b)		AJ236238				
			Actinidia chinensis Planch.	Albert et al. (1992)	L01882					
			Actinidia kolomikta Maxim.	Bremer et al. (2002)			AJ429279	AJ429640	AJ430992	AJ430869
Adoxaceae		*Viburnun*	*Viburnum rhytidophyllum* Graebn.	Bremer et al. (2002)			AJ429391	AJ429736	AJ431103	AJ430979
				Gustafsson et al. (1996)	X87398					
				Oxelman et al. (1999)		AF027273				
Alseuosmiaceae		*Alseuosmia*	*Alseuosmia macrophylla* A.Cunn.	Bremer et al. (2002)			AJ429378	AJ429725	AJ431091	AJ430965
				Gustafsson et al. (1996)	X87377					
				Roels (unpublished)		AF060157				
Apiaceae		*Apium*	*Apium graveolens* L.	Bremer et al. (2002)		AJ429124	AJ429370	AJ429716	AJ431081	AJ430956
				Albert et al. (1992)	L01885					
Apocynaceae		*Alstonia*	*Alstonia scholaris* (L.) R.Br.	Bremer et al. (2002)		AJ011982	AJ429321	AJ429674	AJ431032	AJ430907
				Sennblad & Bremer (1996)	X91760					
Aquifoliaceae		*Ilex*	*Ilex crenata* Thunb.	Olmstead et al. (2000)		AF130206				
			Ilex mitis (L.) Radlk.	Manen et al. (1998)	X98730					
			Ilex sp.	Bremer et al. (2002)			AJ429376	AJ429722	AJ431088	AJ430962
Araliaceae		*Aralia*	*Aralia spinosa* L.	Bremer et al. (2002)		AJ429125	AJ429371	AJ429717	AJ431082	AJ430957
				Chase et al. (1993)	L11166					
Argophyllaceae		*Argophyllum*	*Argophyllum* sp.	Bremer et al. (2002)			AJ429379	AJ429726	AJ431092	AJ430966
				Gustafsson et al. (1996)	X87379					
				Kårehed et al. (1999)		AJ238335				
Asteraceae		*Helianthus*	*Helianthus annuus* L.	Bremer et al. (2002)			AJ429380	AJ429727	AJ431093	AJ430967
				Kanevski et al. (1999)	AF097517					

S1 Table – List of investigated taxa with accession numbers

Family/Group	Tribe	Genus	Species	Citation/Voucher	*rbc*L	*ndh*F	*mat*K	*trn*V	*rps*16	*trn*L
				Kim & Jansen (1995)		L39383				
Balsaminaceae		*Impatiens*	*Impatiens biflora* Walt.	Olmstead et al. (2000)		AF130210				
			Impatiens capensis Meerb.	Bremer et al. (2002)			AJ429280	AJ429641	AJ430993	AJ430870
			Impatiens repens Moon	Morton et al. (1997)	Z80197					
Bignoniaceae		*Jacaranda*	*Jacaranda mimosaefolia* D.Don	Bremer et al. (2002)			AJ429328	AJ429681	AJ431039	AJ430914
			Jacaranda sparrei A.H.Gentry	Spangler & Olmstead (1999)	AF102647	AF102631				
Boraginaceae		*Borago*	*Borago officinalis* L.	Bremer et al. (2002)			AJ429308	AJ429664	AJ431019	AJ430896
				Olmstead et al. (1992)	L11680					
				Olmstead & Reeves (1995)		L36393				
Bruniaceae		*Brunia*	*Brunia albiflora* Phillips	Bremer et al. (2002)			AJ429361	AJ429707	AJ431072	AJ430948
				Backlund & Bremer (1997)	Y10674					
				Roels (unpublished)		AF060159				
Calyceraceae		*Acicarpha*	*Acicarpha tribuloides* Juss.	Bremer et al. (2002)		AJ429129	AJ429381	AJ429728	AJ431094	AJ430968
				Gustafsson et al. (1996)	X87376					
		Boopis	*Boopis anthemoides* Juss.	Michaels et al. (1993)	L13860					
				Kim & Jansen (1995)		L39384				
				Bremer et al. (2002)						AJ430969
			Boopis graminea Phil.	Bremer et al. (2002)			AJ429382	AJ429729	AJ431095	
Caprifoliaceae		*Lonicera*	*Lonicera orientalis* Lam.	Bremer et al. (2002)			AJ430196	AJ429737	AJ431104	AJ430980
				Gustafsson et al. (1996)	X87389					
				Oxelman et al. (1999)		AF027274				
Cardiopteridaceae		*Cardiopteris*	*Cardiopteris quinqueloba* Hassk.	Bremer et al. (2002)		AJ312963	AJ429310	AJ429665	AJ431021	AJ430898
				Savolainen et al. (2000a)	AJ402936					
Clethraceae		*Clethra*	*Clethra alnifolia* L	Bremer et al. (2002)			AJ429281	AJ429526	AJ430994	AJ430871
				Kron & Chase (1983)	L12609					
				Albach et al. (2001a,b)		AJ236242				
Columelliaceae		*Columellia*	*Columellia oblonga* Ruiz and Pav.	Bremer et al. (2002)			AJ429362	AJ429708	AJ431073	AJ430949
				Backlund & Bremer (1997)	Y10675					
				Roels (unpublished)		AF060160				
		Desfontainia	*Desfontainia spinosa* Ruiz and Pav.	Bremer et al. (2002)			AJ429363	AJ429709	AJ431074	AJ430950

S1 Table – List of investigated taxa with accession numbers

Family/Group	Tribe	Genus	Species	Citation/Voucher	*rbc*L	*ndh*F	*mat*K	*trn*V	*rps*16	*trn*L
				Bremer et al. (1994)	Z29670					
				Oxelman et al. (1999)		AJ011988				
Convolvulaceae		Ipomoea	*Ipomoea batatas* (L.) Lam.	Bremer et al. (2002)			AJ429355	AJ429702	AJ431071	AJ430942
			Ipomoea coccinea L.	Olmstead et al. (1993)	L14400	U08918				
Cornaceae		*Cornus*	*Cornus mas* L.	Bremer et al. (2002)			AJ429275	AJ429636	AJ430988	AJ430866
				Xiang et al. (1993)	L11216					
				Roels (unpublished)		AF060161				
Cyrillaceae		Cyrilla	*Cyrilla racemiflora* L.	Bremer et al. (2002)			AJ429282	AJ429527	AJ430995	AJ430872
				Albert et al. (1992)	L01900					
				Anderberg et al. (2002)		AF421051				
Diervillaceae		*Diervilla*	*Diervilla sessilifolia* Buckl.	Bremer et al. (1994)	Z29672					
				Roels (unpublished)		AF060164				
			Diervilla rivularis Gatt.	Bremer et al. (2002)			AJ429392	AJ429738	AJ431105	AJ430981
Dipentodontaceae		*Dipentodon*	*Dipentodon sinicus* Dunn	Bremer et al. (2002)	AJ428890	AJ429102	AJ429273	AJ429634	AJ430986	AJ430865
Dipsacaceae		*Dipsacus*	*Dipsacus sativus* Garsault	Bremer et al. (2002)			AJ429393	AJ429739	AJ431106	AJ430982
				Michaels et al. (1993)	L13864					
				Olmstead et al. (2000)		AF130190				
Ebenaceae		*Diospyros*	*Diospyros kaki* Thunb.	Bremer et al. (2002)			AJ430197	AJ429642	AJ430996	AJ430874
				Morton et al. (1997)	Z80185					
			Diospyros texana Scheele	Olmstead et al. (2000)		AF130213				
Eremosynaceae		*Eremosyne*	*Eremosyne pectinata* Endl.	Bremer et al. (2002)		AJ429120	AJ429364	AJ429710	AJ431075	AJ430951
				Hibach-Jetter et al. (unpubl.)	L47969					
Escalloniaceae		*Escallonia*	*Escallonia coquimbensis* Remy in Gay	Morgan & Soltis (1993)	L11183					
			Escallonia x langleyensis Vilm. and Bois	Roels (unpublished)		AF060165				
			Escallonia rubra Pers.	Bremer et al. (2002)			AJ429365	AJ429711	AJ431076	AJ430952
Fouquieriaceae		*Fouquieria*	*Fouquieria digueti* I.M.Johnst.	Bremer et al. (2002)			AJ429285	AJ429643	AJ430998	AJ430876
			Fouquieria splendens Engelm.	Olmstead et al. (1992)	L11675					
				Albach et al. (2001a,b)		AJ236249				
Garryaceae		*Aucuba*	*Aucuba japonica* Thunb.	Bremer et al. (2002)			AJ429318	AJ429672	AJ431029	AJ430906

S1 Table – List of investigated taxa with accession numbers

Family/Group	Tribe	Genus	Species	Citation/Voucher	*rbc*L	*ndh*F	*mat*K	*trn*V	*rps*16	*trn*L
				Xiang et al. (1993)	L11210					
				Roels (unpublished)		AF060158				
Gelsemiaceae		*Gelsemium*	*Gelsemium sempervirens* Ait.	Bremer et al. (2002)			AJ429322	AJ429675	AJ431033	AJ430908
				Olmstead et al. (1993)	L14397					
				Oxelman et al. (1999)		AJ011984				
Gentianaceae		*Gentiana*	*Gentiana procera* Holm	Olmstead et al. (1993)	L14398					
				Olmstead & Reeves (1995)		L36400				
			Gentiana purpurea L.	Bremer et al. (2002)			AJ429323	AJ429676	AJ431034	AJ430909
Gesneriaceae		*Peltanthera*	*Peltanthera floribunda* Benth.	Bremer et al. (2002)			AJ429330	AJ429682	AJ431041	AJ430916
				Oxelman et al. (1999)	AJ001762	AF027281				
		Streptocarpus	*Streptocarpus caulescens* Vatke	Bremer et al. (2002)			AJ429331	AJ429684	AJ431043	AJ430918
			Streptocarpus holstii Engl.	Olmstead et al. (1993)	L14409					
				Olmstead & Reeves (1995)		L36415				
Goodeniaceae		*Scaevola*	*Scaevola frutescens* K.Krause	Michaels et al. (1993)	L13932					
				Kim & Jansen (1995)		L39385				
			Scaevola sp.	Bremer et al. (2002)			AJ429385	AJ429730	AJ431097	AJ430973
Griseliniaceae		*Griselinia*	*Griselinia littoralis* (Raoul) Raoul	Bremer et al. (2002)			AJ429372	AJ429719	AJ431084	AJ430958
			Griselinia lucida (J.R.Forst. and G.Forst) G.Forst	Xiang et al. (1993)	L11225					
				Olmstead et al. (2000)		AF130205				
Grubbiaceae		*Grubbia*	*Grubbia rosmarinifolia* Berg.	Bremer et al. (2002)		AJ429104	AJ429276	AJ429637	AJ430989	AJ430867
			Grubbia tomentosa (Thunb.) Harms	Morton et al. (1996)	Z83141					
Helwingiaceae		*Helwingia*	*Helwingia japonica* (Thunb. ex Murray) F.Dietrich	Bremer et al. (2002)			AJ430195	AJ429723	AJ431089	AJ430963
				Xiang et al. (1993)	L11226					
				Olmstead et al. (2000)		AF130207				
Icacinaceae		*Apodytes*	*Apodytes dimidiata* E.Mey. ex Bernh.	Bremer et al. (2002)	AJ428895	AJ429109	AJ429311	AJ429666	AJ431022	AJ430899
		Cassinopsis	*Cassinopsis ilicifolia* (Hochst.) Sleumer	Bremer et al. (2002)	AJ428896	AJ429110	AJ429312	AJ429667	AJ431023	AJ430900
		Icacina	*Icacina senegalensis* Juss.	Bremer et al. (2002)	AJ428897	AJ429111	AJ429313	AJ429668	AJ431024	AJ430901
		Pyrenacantha	*Pyrenacantha grandifolia* Engl.	Bremer et al. (2002)			AJ429314	AJ429669	AJ431025	AJ430902

S1 Table – List of investigated taxa with accession numbers

Family/Group	Tribe	Genus	Species	Citation/Voucher	*rbc*L	*ndh*F	*mat*K	*trn*V	*rps*16	*trn*L
			Pyrenacantha malvaefolia Engl.	Savolainen et al. (2000a,b)	AJ402995					
				Kårehed (2001)		AJ312952				
Lamiaceae		*Lamium*	*Lamium album* L.	Bremer et al. (2002)			AJ429332	AJ429685	AJ431044	AJ430919
			Lamium purpureum L.	Kaufmann & Wink (1996)	Z37403					
				Wagstaff & Olmstead (1997)		U78694				
Lecythidaceae		*Barringtionia*	*Barringtionia asiatica* (L.) Kurz	Bremer et al. (2002)			AJ429286	AJ429644	AJ430999	AJ430877
				Morton et al. (1997)	Z80174					
				Anderberg et al. (2002)		AF421044				
Linnaeaceae		*Linnaea*	*Linnaea borealis* L.	Bremer et al. (2002)	AJ428899		AJ429394	AJ429740	AJ431107	AJ430983
				Roels (unpublished)		AF060166				
Loasaceae		*Loasa*	*Loasa loxensis* Humb. Bonpl. and Kunth	Hempel et al. (1995)	U17876					
			Loasa triphylla Juss.	Bremer et al. (2002)			AJ429278	AJ429639	AJ430991	AJ430868
			Loasa vulcanica André	Roels (unpublished)		AF060167				
Loganiaceae		*Logania*	*Logania* sp.	Backlund et al. (2000)		AJ235837				
			Logania vaginalis F.Muell.	Bremer et al. (2002)			AJ429324	AJ429677	AJ431035	AJ430910
				Bremer (1996)	Z68826					
Marcgraviaceae		*Marcgravia*	*Marcgravia rectiflora* Triana and Planch.	Morton et al. (1996)	Z83148					
				Albach et al. (2001a,b)		AJ236263				
			Marcgravia sp.	Bremer et al. (2002)			AJ429289	AJ429646	AJ431001	AJ430879
Martyniaceae		*Proboscidea*	*Proboscidea fragrans* (Lindl.) Decne.	Bremer et al. (2002)			AJ429334	AJ430388	AJ431046	AJ430921
			Proboscidea louisianica (Mill.) Thell.	Albert et al. (1992)	L01946					
				Albach et al. (2001a,b)		AJ236267				
Menyanthaceae		*Menyanthes*	*Menyanthes trifoliata* L.	Bremer et al. (2002)			AJ429386	AJ429731	AJ431098	AJ430974
				Olmstead et al. (1993)	L14006					
				Kim & Jansen (1995)		L39388				
Montiniaceae		*Grevea*	*Grevea* sp.	Bremer et al. (2002)	AJ428898	AJ430426	AJ429357	AJ429704	AJ431066	AJ430944
		Kaliphora	*Kaliphora madagascariensis* Hook.f .	Bremer et al. (2002)		AJ431206	AJ429358	AJ429705	AJ431067	AJ430945
				Savolainen et al. (2000a)	AJ402963					
		Montinia	*Montinia caryophyllacea* Thunb.	Bremer et al. (2002)			AJ429359	AJ429706	AJ431068	AJ430946

S1 Table – List of investigated taxa with accession numbers

Family/Group	Tribe	Genus	Species	Citation/Voucher	*rbc*L	*ndh*F	*mat*K	*trn*V	*rps*16	*trn*L
				Morgan & Soltis (1993)	L11194					
				Olmstead et al. (2000)		AF130178				
Morinaceae		*Morina*	*Morina coulteriana* Royle			AJ429130	AJ429395	AJ429741	AJ431108	AJ430984
				Backlund & Bremer (1997)	Y10706					
Myrsinaceae		*Myrsine*	*Myrsine africana* L.	Bremer et al. (2002)			AJ429290	AJ429647	AJ431002	AJ430880
				Anderberg et al. (1998)	U96652					
				Källersjö et al. (2000)		AF213751				
Oleaceae		*Olea*	*Olea europaea* L.	Bremer et al. (2002)			AJ429335	AJ429687	AJ431047	AJ430922
				Oxelman et al. (1999)	AJ001766	AF027288				
Oncothecaceae		*Oncotheca*	*Oncotheca balansae* Baill.	Bremer et al. (2002)		AJ429114	AJ429320	AJ429673	AJ431031	AJ430529<br>AJ430530
				Savolainen et al. (2000a,b)	AJ131950					
Orobanchaceae		*Cyclocheilon*	*Cyclocheilon somaliense* Oliver	Bremer et al. (2002)		AJ429117	AJ429336	AJ429688	AJ431048	AJ430923
				Wagstaff & Olmstead (1997)	U28871					
		Lindenbergia	*Lindenbergia* sp.	Bremer et al. (2002)			AJ429337	AJ429689	AJ431049	AJ430924
				Oxelman et al. (1999)	AJ001768	AF027286				
Paeoniaceae		*Paeonia*	*Paeonia suffruticosa* Andr.	Savolainen et al. (2000a,b)	AJ402982					
			Paeonia anomala L.	Olmstead et al. (2000)		AF130223				
			Paeonia veitchii Lynch	Bremer et al. (2002)			AJ430198	AJ430199	AJ430201	AJ430200
Paracryphiaceae		*Paracryphia*	*Paracryphia alticola* (Schltr.) v.Steenis	Bremer et al. (2002)		AJ429121	AJ429367	AJ429713	AJ431078	AJ430392
				Savolainen et al. (2000a,b)	AJ402983					
		Quintinia	*Quintinia verdonii* F.Muell	Bremer et al. (2002)			AJ429366	AJ429712	AJ431077	AJ430953
				Gustafsson et al. (1996)	X87394					
				Kårehed et al. (1999)		AJ238344				
Paulowniaceae		*Paulownia*	*Paulownia tomentosa* (Thunb.) Steud.	Bremer et al. (2002)			AJ429339	AJ429690	AJ431051	AJ430926
				Olmstead & Reeves (1995)	L36447	L36406				
Pedaliaceae		*Sesamum*	*Sesamum indicum* L.	Olmstead et al. (1993)	L14408					
				Olmstead & Reeves (1995)		L36413				
			Sesamum orientale L.	Bremer et al. (2002)			AJ429340	AJ429691	AJ431052	AJ430927
Pentaphragmataceae		*Pentaphragma*	*Pentaphragma ellipticum* Poulsen	Bremer et al. (2002)			AJ429387	AJ429732	AJ431099	AJ430975

S1 Table – List of investigated taxa with accession numbers

Family/Group	Tribe	Genus	Species	Citation/Voucher	*rbc*L	*ndh*F	*mat*K	*trn*V	*rps*16	*trn*L
				Cosner et al. (1994)	L18794					
				Olmstead et al. (2000)		AF130183				
Phellinaceae		*Phelline*	*Phelline comosa* Labill.	Kårehed et al. (1999)		AJ238342				
			Phelline lucida Vieill. ex Baill.	Bremer et al. (2002)			AJ429388	AJ429733	AJ431100	AJ430976
				Kårehed et al. (1999)	AJ238347					
Phrymaceae		*Phryma*	*Phryma leptostachya* L.	Bremer et al. (2002)		AJ429118	AJ429341	AJ429692	AJ431053	AJ430928
				Wagstaff & Olmstead (1997)	U28881					
Phyllonomaceae		*Phyllonoma*	*Phyllonoma laticuspis* Engl.	Morgan & Soltis (1993)	L11201					
				Olmstead et al. (2000)		AF130208				
			Phyllonoma ruscifolia Willd. ex Schult.	Bremer et al. (2002)			AJ429377	AJ429724	AJ431090	AJ430964
Pittosporaceae		*Pittosporum*	*Pittosporum tobira* Dryand. ex Ait.	Plunkett et al. (1996)	U50261					
				Olmstead et al. (2000)		AF130201				
			Pittosporum undulatum Vent.	Bremer et al. (2002)			AJ429374	AJ429720	AJ431086	AJ430960
Plantaginaceae		*Antirrhinum*	*Antirrhinum majus* L.	Bremer et al. (2002)			AJ429342	AJ429693	AJ431054	AJ430929
				Olmstead et al. (1992)	L11688					
				Olmstead & Reeves (1995)		L36392				
		Globularia	*Globularia cordifolia* L.	Bremer et al. (2002)			AJ429343	AJ429694	AJ431055	AJ430930
				Oxelman et al. (1999)	AJ001764	AF027282				
		Plantago	*Plantago lanceolata* L.	Olmstead & Reeves (1995)	L36454	L36408				
			Plantago argentea Chaix	Bremer et al. (2002)			AJ429344	AJ429695	AJ431056	AJ430931
Plocospermataceae		*Plocosperma*	*Plocosperma buxifolium* Benth.	Bremer et al. (2002)			AJ429315	AJ429670	AJ431026	AJ430903
				Endress et al. (1996)	Z68829					
				Oxelman et al. (1999)		AJ011985				
Polemoniaceae		*Polemonium*	*Polemonium reptans* L.	Olmstead et al. (1992)	L11687					
			Polemonium caeruleum L.	Anderberg et al. (2002)		AF421070				
			Polemonium pulcherrimum Hook.	Bremer et al. (2002)			AJ429292	AJ429649	AJ431004	AJ430882
Polyosmaceae		*Polyosma*	*Polyosma cunninghamii* Benn.	Bremer et al. (2002)		AJ429122	AJ429368	AJ429714	AJ431079	AJ430954
				Savolainen et al. (2000a,b)	AJ402992					
Primulaceae		*Primula*	*Primula sieboldi* E.Morr	Anderberg et al. (1998)	U96657					
				Källersjö et al. (2000)		AF213757				

S1 Table – List of investigated taxa with accession numbers

Family/Group	Tribe	Genus	Species	Citation/Voucher	*rbc*L	*ndh*F	*mat*K	*trn*V	*rps*16	*trn*L
			Primula veris L.	Bremer et al. (2002)			AJ429293	AJ429650	AJ431005	AJ430883
Roridulaceae		*Roridula*	*Roridula gorgonias* Planch.	Bremer et al. (2002)			AJ429294	AJ429651	AJ431006	AJ430884
				Albert et al. (1992)	L01950					
				Albach et al. (2001a,b)		AJ236270				
Rousseaceae		*Roussea*	*Roussea simplex* Sm.	Bremer et al. (2002)			AJ429389	AJ429734	AJ431101	AJ430977
				Koontz & Soltis (1999)	AF084477					
				Lundberg (2001)		AJ277384				
Rubiacea	Airospermeae	*Boholia*	*Boholia nematostylis* Merr.	Bicknell 1561A (S)			LN680343	LN680278		
				Bremer & Eriksson (2009)	AM117210				AM117286	
				Kainulainen et al. (2009)		AM949848				
				Kainulainen et al. (2013)						HM164312
	Alberteae	*Alberta*	*Alberta magna* E.Mey.	Middleton s.n. 941013/Tonkin 200 (UPS)			LN680336	LN680272		
				Andreasen et al. (1999)	Y18708					
				Bremer et al. (1999)		AJ236282				
				Kainulainen et al. (2009)					FM204701	
				Lantz & Bremer (2004)						AJ620118
	Aleisanthieae	*Aleisanthiopsis*	*Aleisanthiopsis distantiflora* (Merr.) Tange	Tange 46977 (AAU)			LN680337	LN680273		
				Kainulainen et al. (2013)	HM164154	HM164350				HM164306
				Rova (unpublished)					AF242903	
	Anthospermeae	*Anthospermum*	*Anthospermum herbaceum* L.f.	Bremer 3093 (UPS)			LN680339	LN680318<br>LN680330		
				Bremer et al. (1995)	X83623					
				Bremer et al. (1999)		AJ236284				
				Rydin et al. (2008)					EU145496	EU145544
	Argostemmateae	*Argostemma*	*Argostemma hookeri* King	Wanntorp BB 88-27			LN680340	LN680275		
				Bremer (1996)	Z68788					
				Rydin et al. (2009)		FJ695287			FJ695255	
				Rydin et al. (2008)						EU145545

S1 Table – List of investigated taxa with accession numbers

Family/Group	Tribe	Genus	Species	Citation/Voucher	*rbc*L	*ndh*F	*mat*K	*trn*V	*rps*16	*trn*L
	Augusteae	*Augusta*	*Augusta austrocaledonica* (Brongn.) J.H. Kirkbr.	Mouly & Innocente 237 (P)			LN680341	LN680276		
				Mouly et al. (2009)	EU817412					EU817454
				Kainulainen et al. (2013)		HM164352			HM164193	
	Bertiereae	*Bertiera*	*Bertiera longithyrsa* Baker	Kårehed et al. 256 (UPS)			LN680342	LN680277		
				Kainulainen et al. (2013)	HM164158	HM164356			HM164196	HM164311
	Chioccoceae	*Cubanola*	*Cubanola domingensis* (Britton) Aiello	McDowell 001 (??)			LN680349	LN680311		
				Bremer et al. (1995)	X83632					
				Bremer & Eriksson (2009)		AM117345				
				Kainulainen et al. (2009)					FM204718	
				Manns & Bremer (2010)						GQ852490
	Cinchoneae	*Cinchona*	*Cinchona pubescens* Vahl	Bremer 2733 (S)			LN680344	LN680280		
				Bremer et al. (1995)	X83630					
				Backlund et al. (2000)		AJ235843				
				Kainulainen et al. (2009)					FM204714	
				Andersson & Antonelli (2005)						AY538451
	Coffeeae	*Coffea*	*Coffea arabica* L.	Nalapalli et al. (unpublished)	EF044213	EF044213	EF044213	EF044213	EF044213	EF044213
	Colletoecemateae	*Colletoecema*	*Colletoecema dewevrei* (De Wild.) E.M.A. Petit	Lisowski 47195 (K)			LN680345	LN680319 LN680331		
				Rydin et al. (2008)	EU145457	EU145409				EU145532
				Piesschaert et al. (2000)					AF129272	
	Condamineeae	*Condaminea*	*Condaminea corymbosa* (Ruiz & Pav.) DC.	T.D. Pennington & Daza 17436 (MOL)				LN680281		
				Kainulainen et al. (2013)	HM164161					
				Kainulainen et al. (2010)		FJ871950	FJ905347		FJ884645	FJ948381
		Emmenopterys	*Emmenopterys henryi* Oliv.	Robbrecht s.n. (UPS)			LN680353	LN680312		
				Bremer et al. (1999)	Y18715	AJ236294				
				Kainulainen et al. (2009)					FM204719	FM207125
		Pinckneya	*Pinckneya bracteata* (Bartram) Raf.	Massey s.n. 15F			LN680372	LN680304		
				Bremer et al. (1995)	X83661					
				Bremer et al. (1999)		AJ130839				

S1 Table – List of investigated taxa with accession numbers

Family/Group	Tribe	Genus	Species	Citation/Voucher	*rbc*L	*ndh*F	*mat*K	*trn*V	*rps*16	*trn*L
				Kainulainen et al. (2010)					FJ884668	FJ948408
	Coptosapelteae	*Coptosapelta*	*Coptosapelta diffusa* (Champ. ex Benth.) Steenis	Bartholomew et al. 847 (AAU)			LN680346	LN680282		
				Rydin et al. (2008)	EU145453	EU145403			EU145482	EU145527
	Cordiereae	*Stachyarrhena*	*Stachyarrhena heterochroa* Standl.	Persson et al. 821 (GB)	LN680384	LN680392	LN680378	LN680309	LN680874	LN680877
	Coussareeae	*Faramea*	*Faramea multiflora* A.Rich.	Bremer 3331 (UPS)			LN680354	LN680313		
				Bremer (1996)	Z68796					
				Rydin et al. (2008)		EU145424				
				Andersson & Rova (1999)					AF004048	
				Struwe et al. (1998)						AF102422
	Craterispermeae	*Craterispermum*	*Craterispermum longipedunculatum* Verdc.	Luke 9196 (UPS)	LN680381	LN680386	–	–	LN680871	LN680875
	Crossopterygeae	*Crossopteryx*	*Crossopteryx febrifuga* (Afzel. ex G.Don) Benth.	Bremer 3097 (UPS)			LN680348	LN680284		
				Bremer & Eriksson (2009)	AM117223					
				Kainulainen et al. (2009)		AM949851			FM204717	FM207123
	Danaideae	*Danais*	*Danais xanthorrhea* (K. Schum.) Bremek.	Bremer 3079 (UPS)			LN680350	LN680285		
				Bremer (1996)	Z68794					
				Bremer et al. (1999)		AJ236293				
				Bremer & Eriksson (2009)					AM117297	
				Backlund et al. (2007)						DQ662138
	Dunnieae	*Dunnia*	*Dunnia sinensis* Tutch.	Zhuhai 12, Ge et al. 2002			LN680352	LN680287		
				Rydin et al. (2008)	EU145467	EU145444			EU145517	EU145587
	Foonchewieae	*Foonchewia*	*Foonchewia guangdongensis* R.J.Wang & H.Z.Wen	Wen & Wang (2012)	JQ002642	JQ002646	–	–	JQ002638	–
	Gaertnereae	*Gaertnera*	*Gaertnera phyllosepala* Baker	Kårehed et al. 274 (UPS)		LN680388	LN680355	LN680288		
				Razafimandimbison et al. (2008)	AM945288				AM945311	
			Gaertnera sp.	Backlund et al. (2007)						DQ662139
	Gardenieae	*Gardenia*	*Gardenia hansemannii* K.Schum.	Drozd & Molem 1998-11-13			LN680356	LN680289		
				Novotny et al. (2002)	AJ318446				AJ320077	

S1 Table – List of investigated taxa with accession numbers

**Family/Group**	**Tribe**	**Genus**	**Species**	**Citation/Voucher**	***rbc*L**	***ndh*F**	***mat*K**	***trn*V**	***rps*16**	***trn*L**
				Kainulainen et al. (2009)		AM949852				FM207126
	Greeneae	*Greenea*	*Greenea oblonga* Craib	Larsen K. & Larsen S.S. 33451 (P)	–			LN680320		
				Kainulainen et al. (2013)		HM164371	HM119539			
				Mouly et al. (2009)					EU817439	EU817459
	Guettardeae	*Guettarda*	*Guettarda speciosa* L.	Rova 2492 (GB)			LN680358	LN680291		
				Andersson & Antonelli (2005)	AY538485					
				Manns & Bremer (2010)		GQ852213				GQ852509
				Rova (unpublished)					AF242964	
	Hamelieae	*Deppea*	*Deppea grandiflora* Schltdl.	Bremer 2724 (UPS)			LN680351	LN680286		
				Manns & Bremer (2010)		GQ852198				GQ852493
				Bremer et al. (1995)	X83633					
				Bremer & Eriksson (2009)					AM117299	
	Henriquezieae	*Henriquezia*	*Henriquezia nitida* Spruce ex Benth.	Liesner 8531 (MO)	–		LN680360	LN680322		
				Kainulainen et al. (2013)		HM164374			HM164211	HM164325
	Hillieae	*Hillia*	*Hillia triflora* (Oerst.) C.M.Taylor	Bremer 3101 (UPS)			LN680361	LN680293		
				Bremer et al. (1995)	X83642					
				Bremer et al. (1999)		AJ236298				
				Bremer & Eriksson (2009)					AM117315	
				Manns & Bremer (2010)						GQ852513
	Hymenodictyeae	*Hymenodictyon*	*Hymenodictyon floribundum* B.L.Rob	Puff 861109-3/1 (WU)				LN680294		
				Razafimandimbison & Bremer (2002)	AJ347015					
				Rydin et al. (2008)		EU145411				
				Andersson & Antonelli (2005)			AY538392			
				Andersson & Rova (1999)					AF004058	
				Manns & Bremer (2010)						GQ852517
	Insertae sedis	*Glionnetia*	*Glionnetia sericea* (Baker) Tirveng.	Beaver 17 (S)			LN680357	LN680290		
				Razafimandimbison et al. (2011)	HM536223	HM536217			HM536229	HM536235
	Isertieae	*Isertia*	*Isertia laevis* (Triana) B.M.Boom	Bremer 3364 (UPS)			LN680362	LN680295		

S1 Table – List of investigated taxa with accession numbers

Family/Group	Tribe	Genus	Species	Citation/Voucher	*rbc*L	*ndh*F	*mat*K	*trn*V	*rps*16	*trn*L
				Bremer & Thulin (1998)	Y11852					
				Manns & Bremer (2010)		GQ852226				GQ852519
				Bremer & Eriksson (2009)					AM117319	
	Ixoreae	*Ixora*	*Ixora coccinea* L.	Bremer 2719 (UPS)			LN680363	LN680296		
				Kainulainen et al. (2013)	HM164167	HM164376				
				Mouly et al. (2007)					EF205641	
				Mouly et al. (2009)						EU817464
	Jackieae	*Jackiopsis*	*Jackiopsis ornata* (Wall.) Ridsdale	K.S. Tan s.n.			LN680364	LN680297		
				Razafimandimbison et al. (2011)	HM536219	HM536213			HM536225	HM536231
	Knoxieae	*Pentas*	*Pentas lanceolata* (Forssk.) Deflers	Bremer, 2702 (S)				LN680303		
				Bremer et al. (1995)	X83659					
				Bremer et al. (1999)		AJ236304				
				Nakamura et al. (2006)			AB247151			
				Kårehed & Bremer (2007)					AM266875	AM266963
	Lasiantheae	*Lasianthus*	*Lasianthus kilimandscharicus* K.Schum.	Lantz 119 (UPS)			LN680365	LN680314		
				Bremer & Eriksson (2009)	AM117237				AM117327	
				Rydin et al. (2008)		EU145426				
				Backlund et al. (2007)						DQ662147
	Luculieae	*Luculia*	*Luculia grandifolia* Ghose	Bremer et al. (1995)	X83648		–			
			Luculia gratissima Sweet	Bremer et al. (2002)			–	AJ429678	AJ431036	AJ430911
				Oxelman et al. (1999)		AJ011987				
	Mitchelleae	*Mitchella*	*Mitchella repens* L.	Bremer 2714 (S)			LN680366	LN680298		
				Bremer (1996)	Z68805					
				Razafimandimbison et al. (2008)		AM945258				
				Andersson & Rova (1999)					AF001441	
				Razafimandimbison et al. (2009)						FJ906973
		Mitriostigma	*Mitriostigma axillare* Hochst.	Bremer 2705 (UPS)				LN680299		
				Bremer et al. (1995)	X83650					
				Kainulainen et al. (2013)		HM164381	HM119550		HM164216	HM164329

S1 Table – List of investigated taxa with accession numbers

Family/Group	Tribe	Genus	Species	Citation/Voucher	*rbc*L	*ndh*F	*mat*K	*trn*V	*rps*16	*trn*L
	Morindeae	*Morinda*	*Morinda citrifolia* L.	Bremer 3302 (UPS)			LN680367	LN680300		AF152616
				Novotny et al. (2002)	AJ318448					
				Bremer et al. (1999)		AJ236300				
				Vicentini & Stevens (unpubl.)					EU872844	
	Mussaendeae	*Mussaenda*	*Mussaenda arcuata* Poir.	McPehrson 16213 (MO)				LN680301		
				Bremer & Thulin (1998)	Y11854					
				Bremer et al. (1999)		AJ236301				
				Kainulainen et al. (2013)			HM119551			
				Kainulainen et al. (2009)					FM204721	FM207128
	Naucleeae	*Cephalanthus*	*Cephalanthus occidentalis* L.	Forbes s.n. (S)				LN680279		
				Bremer et al. (1995)	X83629					
				Bremer et al. (1999)		AJ236288				
				Andersson & Antonelli (2005)			AY538377			
				Andersson & Rova (1999)					AF004033	
				Rova et al. (2002)						AF152692
	Octotropideae	*Paragenipa*	*Paragenipa lancifolia* (Bojer ex Baker) Tirveng. & Robbr.	Persson 156 (GB)			LN680370	LN680302		
				Andreasen & Bremer (2000)	AJ286707					
				Kainulainen et al. (2013)		HM164385				
				Andersson & Rova (1999)					AF004066	
				Kainulainen et al. (2013)<br>Rova et al. (2002)						HM164330<br>AF152672
	Ophiorrhizeae	*Ophiorrhiza*	*Ophiorrhiza mungos* L.	Bremer 3301 (UPS)			LN680368	LN680315		
				Bremer et al. (1995)	X83656					
				Bremer et al. (1999)		AJ130838				
				Andersson & Rova (1999)					AF004064	
				Backlund et al. (2007)						DQ662151
	Paederieae	*Paederia*	*Paederia foetida* L.	Wong & Keong s.n. (KLU)			LN680369	LN680323		
				Andersson (unpublished)	AF332373					
				Andersson & Rova (1999)					AF004065	

S1 Table – List of investigated taxa with accession numbers

Family/Group	Tribe	Genus	Species	Citation/Voucher	*rbc*L	*ndh*F	*mat*K	*trn*V	*rps*16	*trn*L
				Rova et al. (2002)						AF152619
			Paederia bojeriana (A.Rich. ex DC.)	Rydin et al. (2009)		FJ695318				
	Palicoureae	*Palicourea*	*Palicourea crocea* (Sw.) Schult.	Bremer & Eriksson (2009)	AM117253		–	–		
				Razafimandimbison et al. (2008)		AM945280				
				Andersson & Taylor (unpubl.)					AF147510	
			Palicourea guianensis Aubl.	Rova et al. (2002)			–	–		AF152615
	Pavetteae	*Pavetta*	*Pavetta abyssinica* Fresen.	De Block 6 (BR)			LN680371	LN680324<br>LN680333		
				Andreasen & Bremer (1996)	Z68863					
				Kainulainen et al. (2009)		AM949854			FM204726	FM207133
	Posoquerieae	*Posoqueria*	*Posoqueria latifolia* (Rudge) Roem. & Schult.	SU-C-88.10 Trädg.herb. KA				LN680325		
				Andreasen & Bremer (1996)	Z68850					
				Kainulainen et al. (2009)		AM949855			FM204728	FM207135
				Kainulainen et al. (2010)			FJ905325			
	Prismatomerideae	*Prismatomeris*	*Prismatomeris albidiflora* Thwaites	Martin Marie 89 (P)	LN680382	LN680390	–	–	LN680872	–
	Psychotrieae	*Cremocarpon*	*Cremocarpon lantzii* Bremek.	Razafimandimbison 517 (UPS)		LN680387	LN680347	LN680283		
				Bremer & Eriksson (2009)	AM117222				AM117296	AM117356
		Psychotria	*Psychotria holtzii* (K.Schum.) E.M.A.Petit	Q. Luke 8342 (UPS)	LN680383	LN680391	–	–	LN680873	LN680876
	Putorieae	*Plocama*	*Plocama pendula* Aiton	Bremer (1996)	Z68816		–	–		
				Rydin et al. (2009)		FJ695329			FJ695276	
				Backlund et al. (2007)						DQ662162
	Retiniphylleae	*Retiniphyllum*	*Retiniphyllum pilosum* (Spruce ex Benth.) Müll. Arg.	Andersson (unpublished)	AF331654		–	–		
				Kainulainen et al. (2013)		HM164392				
				Andersson & Rova (1999)					AF004076	
				Kainulainen et al. (2009)						FM207137
	Rondeletieae	*Rondeletia*	*Rondeletia odorata* Jacq.	Bremer & Andreasen 3504 (UPS)			LN680374	LN680305		
				Bremer & Thulin (1998)	Y11857					

S1 Table – List of investigated taxa with accession numbers

Family/Group	Tribe	Genus	Species	Citation/Voucher	*rbc*L	*ndh*F	*mat*K	*trn*V	*rps*16	*trn*L
				Backlund et al. (2000)		AJ235845				
				Rydin et al. (2008)					EU145490	
				Rova et al. (2002)						AF152741
	Rubieae	*Rubia*	*Rubia tinctorum* L.	Bremer 3300 (UPS)			LN680375	LN680326		
				Bremer et al. (1995)	X83666					
				Manen (unpublished)		DQ359167				
				Rydin et al. (2009)						FJ695421
			Rubia fruticosa Aiton	Andersson & Rova (1999)					AF004078	
	Sabiceeae	*Hekistocarpa*	*Hekistocarpa minutiflora* Hook.f.	Dessein s.n. (P)				LN680292		
				Dessein et al. (2001)	AF332366				AF332367	
				Kainulainen et al. (2013)		HM164373	HM119541			HM164324
		Sabicea	*Sabicea diversifolia* Pers.	Bremer et al. 4018-B18 (UPS)				LN680306		
				Rydin et al. (2008)	EU145459	EU145415			EU145494	
				Kainulainen et al. (2013)			HM119568			
				Alejandro et al. (2005)						AJ847396
	Schizocoleeae	*Schizocolea*	*Schizocolea linderi* (Hutch. & Dalziel) Bremek.	Adam 789 (P)			LN680376			
				Adam 20116 (UPS)				LN680307		
				Bremer & Eriksson (2009)	AM117272					
				Rydin et al. (2009)		FJ695334				
				Rydin et al. (2008)					EU145498	EU145546
	Schradereae	*Schradera*	*Schradera subandina* K.Krause	Clark & Watt 783 (QCNE)			LN680377	LN680308		
				Bremer & Thulin (1998)	Y11859					
				Razafimandimbison et al. (2008)		AM945264			AM945313	
			Schradera sp.	Rova et al. (2002)						AF152613
	Scyphiphoreae	*Scyphiphora*	*Scyphiphora hydrophyllacea* C.F.Gaertn.	K Bremer *et al*. 99 (S)				LN680317		
				Bremer et al. (1999)	Y18717	AJ236311				
				Kainulainen et al. (2010)			FJ905327			

S1 Table – List of investigated taxa with accession numbers

**Family/Group**	**Tribe**	**Genus**	**Species**	**Citation/Voucher**	***rbc*L**	***ndh*F**	***mat*K**	***trn*V**	***rps*16**	***trn*L**
				Mouly et al. (2009)					EU817450	EU817475
	Sipaneeae	*Dendrosipanea*	*Dendrosipanea spigelioides* Ducke	Prance et al. 16199 (S)				LN680310		
				Kainulainen et al. (2013)	HM164162					
				Kainulainen et al. (2010)		FJ871943	FJ905324		FJ884627	FJ948360
	Spermacoceae	*Hedyotis*	*Hedyotis fruticosa* L.	Larsson & Pyddoke 22 (S)		LN680389	LN680359	LN680321<br>LN680332		
				Bremer (1996)	Z68799					
				Groeninckx et al. (2009)						EU543098
			Hedyotis macrostegia Stapf	Andersson & Rova (1999)					AF002767	
		Kohautia	*Kohautia caespitosa* Schnizl.	Bremer (1996)	Z68800		–	–		
				Rydin et al. (2009)		FJ695303				
				Bremer & Eriksson (2009)					AM117324	
				Rydin et al. (2008)						EU145573
	Steenisieae	*Steenisia*	*Steenisia pleurocarpa* (Airy Shaw) Bakh.f.	Puff BF 990619-1/4 (WU)				LN680327		
				Bremer & Eriksson (2009)	AM117279					
				Kainulainen et al. (2013)		HM164396				
				Kainulainen et al. (2010)			FJ905328			
				Kainulainen et al. (2009)					FM204735	FM207142
	Theligoneae	*Theligonum*	*Theligonum cynocrambe* L.	Thor 654 (S)			LN680379	LN680328<br>LN680334		
				Bremer et al. (1995)	X83668					
				Rydin et al. (2009)		FJ695339				FJ695427
				Andersson & Rova (1999)					AF004087	
	Trailliaedoxeae	*Trailliaedoxa*	*Trailliaedoxa gracilis* W.W.Sm. & Forrest	Boufford & al 35041 (MO)			LN680380	LN680329<br>LN680335		
				Kainulainen et al. (2013)	HM164183	HM164400			HM164227	HM164339
	Urophylleae	*Amphidasya*	*Amphidasya ambigua* (Standl.) Standl.	Clark & Watt 736 (UPS)		LN680385	LN680338	LN680274		
				Bremer & Thulin (1998)	Y11844					
				Piesschaert et al. (2000)					AF129271	
				Rydin et al. (2008)						EU145576

S1 Table – List of investigated taxa with accession numbers

Family/Group	Tribe	Genus	Species	Citation/Voucher	*rbc*L	*ndh*F	*mat*K	*trn*V	*rps*16	*trn*L
	Vanguerieae	*Psydrax*	*Psydrax obovata* (Klotzsch ex Eckl. & Zeyh.) Bridson	Bremer & Bremer 3762 (UPS)			LN680373	LN680316		
				Kainulainen et al. (2013)	HM164176	HM164388			HM164220	
				Lantz & Bremer (2004)						AJ620161
Sapotaceae		*Manilkara*	*Manilkara zapota* (L.) van Royen	Bremer et al. (2002)			AJ429295	AJ429652	AJ431007	AJ430885
				Albert et al. (1992)	L01932					
				Källersjö et al. (2000)		AF213732				
Schlegeliaceae		*Schlegelia*	*Schlegelia parviflora* (Oerst.) Monach.	Bremer et al. (2002)			AJ429345	AJ429696	AJ431057	AJ430932
				Olmstead & Reeves (1995)	L36448	L36410				
Scrophulariaceae		*Androya*	*Androya decaryi* H. Perrier	Oxelman et al. (unpublished)	AJ001756	AF027276				
				Bremer et al. (2002)			AJ429329	AJ429530	AJ431040	AJ430915
		Buddleja	*Buddleja asiatica* Lour.	Bremer et al. (2002)			AJ429346	AJ429697	AJ431058	AJ430933
			Buddleja davidii Franch.	Oxelman et al. (1999)	AJ001757					
				Olmstead & Reeves (1995)		L36394				
		Myoporum	*Myoporum mauritianum* A.DC.	Olmstead & Reeves (1995)	L36445	L36403				
			Myoporum montanum R.Br.	Bremer et al. (2002)			AJ429347	AJ429698	AJ431059	AJ430934
		Selago	*Selago thomsoni* Rolfe ex Oliver	Bremer et al. (2002)			AJ429348	AJ429699	AJ431060	AJ430935
			Selago thunbergii Choisy	Olmstead & Reeves (1995)	L36450	L36412				
		Scrophularia	*Scrophularia arguta* Sol. ex Ait.	Bremer et al. (2002)			AJ429349	AJ429531	AJ431061	AJ430936
			Scrophularia sp.	Olmstead & Reeves (1995)	L36449	L36411				
Solanaceae		*Nicotiana*	*Nicotiana tabacum* L.	Shinozaki et al. (1986)	Z00044		Z00044	Z00044	Z00044	Z00044
				Olmstead et al. (1993)		L14953				
Sphenocleaceae		*Sphenoclea*	*Sphenoclea zeylanica* Gaertn.	Bremer et al. (2002)		AJ429119	AJ429360	AJ429534	AJ431069	AJ430947
				Cosner et al. (1994)	L18798					
Stilbaceae		Stilbe	*Stilbe albiflora* E. Mey.	Oxelman et al. (1999)		AF027287				
			Stilbe ericoides L.	Bremer et al. (2002)			AJ429350	AJ429532	AJ431062	AJ430937
			Stilbe vestita Berg.	Bremer (1996)	Z68827					
Stylidiaceae		*Stylidium*	*Stylidium bulbiferum* Benth.	Bremer et al. (2002)			AJ429390	AJ429735	AJ431102	AJ430978
			Stylidium calcaratum R. Br.	Laurent et al. (1998)	AJ225053	AJ225079				
Styracaceae		*Halesia*	*Halesia carolina* L.	Bremer et al. (2002)			AJ429298	AJ429655	AJ431010	AJ430082

S1 Table – List of investigated taxa with accession numbers

Family/Group	Tribe	Genus	Species	Citation/Voucher	*rbc*L	*ndh*F	*mat*K	*trn*V	*rps*16	*trn*L
				Morton et al. (1997)	Z80190					
			Halesia tetraptera L.	Olmstead et al. (2000)		AF130214				
		Styrax	*Styrax americanum* Lam.	Kron & Chase (1983)	L12623					
				Olmstead et al. (2000)		AF130215				
			Styrax officinale L.	Bremer et al. (2002)			AJ429300	AJ429657	AJ431011	AJ430888
Symplocaceae		*Symplocos*	*Symplocos bogotensis* Brand	Bremer et al. (2002)			AJ429301	AJ429658	AJ431012	AJ430889
				Anderberg et al. (2002)		AF421074				
			Symplocos costata Choisy ex Zoll.	Morton et al. (1997)	Z80192					
Ternstroemiaceae		*Ternstroemia*	*Ternstroemia gymnanthera* Sprague	Bremer et al. (2002)			AJ429302	AJ429659	AJ431013	AJ430890
				Anderberg et al. (2002)		AF421076				
			Ternstroemia stahlii Krug and Urb.	Morton et al. (1997)	Z80211					
(Pentaphyllaceae)		*Pentaphylax*	*Pentaphylax euryoides* Gardn. and Champ.	Bremer et al. (2002)	AJ428891	AJ429106	AJ429291	AJ429648	AJ431003	AJ430881
(Sladeniaceae)		*Sladenia*	*Sladenia celastrifolia* Kurz				AJ429297	AJ429654	AJ431009	AJ430081
				Savolainen et al. (2000a,b)	AJ403004					
				Anderberg et al. (2002)		AF421081				
Tetrachondraceae		*Polypremum*	*Polypremum procumbens* L.	Bremer et al. (2002)			AJ429351	AJ429700	AJ431063	AJ430938
				Oxelman et al. (1999)	AJ011989	AJ011986				
		Tetrachondra	*Tetrachondra hamiltonii* Petrie	Wagstaff & Olmstead (1997)	U28885					
			Tetrachondra patagonica Skottsb.	Oxelman et al. (1999)		AF027272				
				Bremer et al. (2002)			AJ429352	AJ430389	AJ431064	AJ430939
Tetrameristaceae		*Pelliciera*	*Pelliciera rhizophoreae* Planch. and Triana	Bremer et al. (2002)	AJ428893		AJ429303	AJ429660	AJ431014	AJ430891
				Anderberg et al. (2002)		AF421069				
		Tetramerista	*Tetramerista sp.*	Bremer et al. (2002)		AJ429108	AJ429304	AJ429528	AJ431015	AJ430892
				Morton et al. (1997)	Z80199					
Theaceae		*Camellia*	*Camellia japonica* L.	Kron & Chase (1983)	L12602					
				Olmstead et al. (2000)		AF130216				
			Camellia sinensis Kuntze	Bremer et al. (2002)			AJ429305	AJ429661	AJ431016	AJ430893
		Schima	*Schima superba* Gardn. and Champ.	Morton et al. (1997)	Z80208					

S1 Table – List of investigated taxa with accession numbers

Family/Group	Tribe	Genus	Species	Citation/Voucher	*rbc*L	*ndh*F	*mat*K	*trn*V	*rps*16	*trn*L
				Anderberg et al. (2002)		AF421073				
				Bremer et al. (2002)			AJ429306	AJ429662	AJ431017	AJ430894
Theophrastaceae		*Theophrasta*	*Theophrasta americana* L.	Anderberg et al. (1998)	U96649					
				Källersjö et al. (2000)		AF213762				
				Bremer et al. (2002)			AJ429307	AJ429663	AJ431018	AJ430895
Torricellia group										
(Aralidiaceae)		*Aralidium*	*Aralidium pinnatifidum* Miq.	Bremer et al. (2002)		AJ429126		AJ429718	AJ431083	AJ430083
				Xiang & Soltis (unpublished)	AF299087					
				Plunkett et al. (1997)			U58627			
(Melanophyllaceae)		*Melanophylla*	*Melanophylla alnifolia* Baker	Plunkett et al. (1996)	U50254					
				Albach et al. (2001a,b)		AJ236244				
			Melanophylla sp.	Bremer et al. (2002)			AJ429373	AJ430390	AJ431085	AJ430959
(Torricelliaceae)		*Torricellia*	*Torricellia tiliaefolia* DC.	Xiang & Soltis (unpublished)	AF299089					
				Bremer et al. (2002)		AJ429127	AJ429375	AJ429721	AJ431087	AJ430961
Tribelaceae		*Tribeles*	*Tribeles australis* Phil.	Savolainen et al. (2000a,b)	AJ403010					
				Bremer et al. (2002)		AJ429123	AJ429369	AJ429715	AJ431080	AJ430955
Vahliaceae		*Vahlia*	*Vahlia capensis* Thunb.	Morgan & Soltis (1993)	L11208					
				Bremer et al. (2002)		AJ429112	AJ429316	AJ429671	AJ431027	AJ430904
Valerianaceae		*Valeriana*	*Valeriana fauriei* Briquet	Olmstead et al. (2000)		AF130192				
			Valeriana hirtella Kunth	Backlund & Bremer (1997)	Y10699					
				Bremer et al. (2002)			AJ429396	AJ429742	AJ431109	
			Valeriana officinalis L.	Bremer et al. (2002)						AJ431680
Verbenaceae		*Verbena*	*Verbena bracteata* Cav. ex Lag. and Rodr.	Olmstead & Reeves (1995)		L36418				
			Verbena officinalis L.	Kaufmann & Wink (1996)	Z37473					
			Verbena rigida Spreng.	Bremer et al. (2002)			AJ429353	AJ429701	AJ431065	AJ430940
Vitaceae		*Vitis*	*Vitis aestivalis* Michx.	Albert et al. (1992)	L01960					
			Vitis vinifera L.	Bremer et al. (2002)		AJ429103	AJ429274	AJ429635	AJ430987	AJ430864

S1 Table – List of investigated taxa with accession numbers

References

Albach DC, Soltis DE, Chase MW, Soltis PS (2001a) Phylogenetic placement of the enigmatic angiosperm *Hydrostachys*. Taxon 50: 763–805.

Albach DC, Soltis PS, Soltis DE (2001b) Phylogenetic analysis of asterids based on sequences of four genes. Ann Mo Bot Gard 88: 163–212.

Albert VA, Williams SE, Chase MW (1992) Carnivorous plantsphylogeny and structural evolution Science. 257: 1491–1495.

Alejandro GD, Razafimandimbison SG, Liede-Schumann S (2005) Polyphyly of *Mussaenda* inferred from ITS and *trnT-F* data and its implication for generic limits in Mussaendeae (Rubiaceae). Am J Bot 92: 544–557.

Anderberg AA, Rydin C, Källersjö M (2002) Phylogenetic relationships in the order Ericales s.l.: analyses of molecular data from five genes from the plastid and mitochondrial genomes. Am J Bot 89: 677–687.

Anderberg AA, Ståhl B, Källersjö M (1998) Phylogenetic relationships in the Primulales inferred from cpDNA *rbcL* sequence data. Plant Syst Evol 211: 93–102.

Andersson L, Antonelli A (2005) Phylogeny of the tribe Cinchoneae (Rubiaceae), its position in Cinchonoideae, and description of a new genus, *Ciliosemina*. *Taxon* 54: 17–28.

Andersson L, Rova JHE (1999) The *rps16* intron and the phylogeny of the Rubioideae (Rubiaceae). Plant Syst Evol 214: 161–186.

Andreasen K, Baldwin B, Bremer B (1999) Phylogenetic utility of the nuclear rDNA ITS region in subfamily Ixoroideae (Rubiaceae): comparisons with cpDNA *rbcL* sequence data. Plant Syst Evol 217: 119–135.

Andreasen K, Bremer B (1996) Phylogeny of the subfamily Ixoroideae (Rubiaceae). Opera Bot Belg 7: 119–138.

Andreasen K, Bremer B (2000) Combined phylogenetic analysis in the Rubiaceae-Ixoroideae: morphology, nuclear and chloroplast DNA data. Am J Bot 87: 1731–1748.

Backlund M, Bremer B (1997) Phylogeny of the Asteridae s. str. based on *rbcL* sequences, with particular reference to the Dipsacales. Plant Syst Evol 207: 225–254.

Backlund M, Bremer B, Thulin M (2007) Paraphyly of Paederieae, recognition of Putorieae and expansion of *Plocama* (Rubiaceae–Rubioideae). *Taxon* 56: 315–328.

Backlund, M., Oxelman, B., and Bremer, B. (2000). Phylogenetic relationships within the Gentianales based on *ndhF* and *rbcL* sequences, with particular reference to the Loganiaceae (Rubiaceae – Rubioideae). *Am. J. Bot.* 87: 1029–1043.

Bremer, B (1996) Phylogenetic studies within Rubiaceae and relationships to other families based on molecular data. Opera Bot Belg 7: 33–50.

Bremer B, Andreasen K, Olsson D (1995) Subfamilial and tribal relationships in the Rubiaceae based on *rbcL* sequence data. Ann Mo Bot Gard 82: 383–397.

Bremer B, Bremer K, Heidari N, Erixon P, Olmstead RG, Anderberg AA, Källersjö M, Barkhordarian E (2002) Phylogenetics of asterids based on 3 coding and 3 non-coding chloroplast DNA markers and the utility of non-coding DNA at higher taxonomic levels Mol. Phylogenet Evol *24*: 274–301.

Bremer B, Eriksson T (2009) Timetree of Rubiaceae – phylogeny and dating the family, subfamilies and tribes. Int J Plant Sci 170: 766–793.

Bremer B, Jansen RK, Oxelman B, Backlund M, Lantz H, Kim KJ (1999) More characters or more taxa for a robust phylogeny – case study from the coffee family (Rubiaceae). Syst Biol 48: 413–435.

Bremer B, Olmstead RG, Struwe L, Sweere JA (1994) *rbcL* sequences support exclusion of *Retzia*, *Desfontainia*, and *Nicodemia* from the Gentianales. Plant Syst Evol 190: 213–230.

Bremer B, Thulin M (1998). Collapse of Isertiaea, re-establishment of Mussaendeae, and a new genus of Sabiceeae (Rubiaceae); phylogenetic relationships based on *rbcL* data. Plant Syst Evol 211: 71–92.

S1 Table – List of investigated taxa with accession numbers

Chase MW, Soltis DE, Olmstead RG, Morgan D, Les DH, Mishler BD, Duvall MR, Price RA, Hills HG, Qiu YL, Kron KA, Rettig JH, Conti E, Palmer JD, Manhart JR, Sytsma KJ, Michaels HJ, Kress JW, Karol KG, Clark DW, Hedren M, Gaut BS, Jansen RK, Kim KJ, Wimpee CF, Smith JF, Furnier GR, Strauss SH, Xiang QY, Plunkett GM, Soltis PS, Swensen SM, Williams SE, Gadek PA, Quinn CJ, Equiarte LE, Golenberg E, Learn Jr GH, Graham SW, Barrett SCH, Dayanandan S, Albert VA (1993) Phylogenetics of seed plants: An analysis of nucleotide sequences from the plastid gene *rbcL*. Ann Mo Bot Gard 80: 528–580.

Cosner ME, Jansen RK, Lammars TG (1994) Phylogenetic relationships in the Campanulales based on *rbcL* sequences Plant Syst Evol 190: 79–95.

Dessein S, Andersson L, Robbrecht E, Smets E (2001) *Hekistocarpa* (Rubiaceae): a member of an emended tribe Virectarieae. Plant Syst Evol 229: 59–78.

Endress ME, Sennblad B, Nilsson S, Civeyrel L, Chase MW, Huysmans S, Grafström E, Bremer B (1996) A phylogenetic analysis of Apocynaceae *s.str.* and some related taxa in Gentianales: a multidisciplinary approach. Opera Bot Belg 7: 59–102.

Groeninckx I, Dessein S, Ochoterena H, Persson C, Motley TJ, Kårehed J, Bremer B, Huysmans S, Smets E (2009) Phylogeny of the herbaceous tribe Spermacoceae (Rubiaceae) based on plastid DNA data. Ann Mo Bot Gard 96: 109–132.

Gustafsson MHG, Backlund A, Bremer B (1996) Phylogeny of the Asterales sensu lato based on *rbcL* sequences with particular reference to the Goodeniaceae. Plant Syst Evol 199: 217–242.

Hedrén M, Chase MW, Olmstead RG (1995) Relationships in the Acanthaceae and related families as suggested by cladistic analysis of *rbcL* nucleotide sequences. Plant Syst Evol 194: 93–109.

Hempel AL, Reeves PA, Olmstead RG, Jansen RK (1995) Implications of *rbcL* sequence data for higher order relationships of the Loasaceae and the anomalous aquatic plant *Hydrostachys* (Hydrostachyaceae). Plant Syst Evol 194: 25–37.

Kainulainen K, Mouly A, Khodabandeh A, Bremer B (2009) Molecular phylogenetic analysis of the tribe Alberteae (Rubiaceae), with description of a new genus, *Razafimandimbisonia*. Taxon 58: 757–768.

Kainulainen K, Persson C, Eriksson T, Bremer B (2010) Molecular systematics and morphological character evolution of the Condamineeae (Rubiaceae). Am J Bot 97: 1961–1981.

Kainulainen K, Razafimandimbison SG, Bremer B (2013) Phylogenetic relationships and new tribal delimitations in subfamily Ixoroideae (Rubiaceae). Bot J Linn Soc 173: 387–406.

Kainulainen K, Bremer B (2014) Phylogeny of *Euclinia* and allied genera of Gardenieae (Rubiaceae), and description of *Melanoxerus*, en endemic genus of Madagascar. Taxon 63: 819–830.

Källersjö M, Bergqvist G, Anderberg AA (2000) Generic realignment in primuloid families of the Ericales s.l.: a phylogenetic analysis based on DNA sequences from three chloroplast genes and morphology. Am J Bot 87: 1325–1341.

Kanevski I, Maliga P, Rhoades DF, Gutteridge S (1999) Plastome engineering of ribulose-l,5 bisphosphate carboxylase/oxygenase in tobacco to form a sunflower large subunit and tobacco small subunit hybrid. Plant Physiol 119: 133–141.

Kårehed J (2001) Multiple origin of the tropical forest tree family Icacinaceae. Am J Bot 88: 2259– 2274.

Kårehed J, Bremer B (2007) The systematics of Knoxieae (Rubiaceae) – molecular data and their taxonomic consequences. Taxon 56: 1051–1076.

Kårehed J, Lundberg J, Bremer B, Bremer K (1999) Evolution of the Australasian families Alseuosmiaceae, Argophyllaceae, and Phellinaceae. Syst Bot 24: 660–682.

Kaufmann M, Wink M (1996) Phylogenetic relationships between some members of the subfamily Lamioideae family Labiatae inferred from nucleotide sequences of the *rbcL* gene. Bot Acta *109:* 139–148.

Kim KJ, Jansen RK (1995) *ndhF* sequence evolution and the major clades in the sunflower family. Proc Natl Acad Sci USA 9: 10379–10383.

S1 Table – List of investigated taxa with accession numbers

Koontz JA, Soltis DE (1999) DNA sequence data reveal polyphyly of Brexioideae Brexiaceae; Saxifragaceae sensu lato. Plant Syst Evol 219: 199–208.

Kron KA, Chase MW (1983) Systematics of the Ericaceae, Empetraceae, Epacridaceae and related taxa based upon *rbcL* sequence data. Ann Mo Bot Gard 80: 735–741.

Lantz H, Bremer B (2004) Phylogeny inferred from morphology and DNA data: characterizing well-supported groups in Vanguerieae (Rubiaceae). Bot J Linn Soc 146: 257–283.

Laurent N, Bremer B, Bremer K (1998) Phylogeny and generic interrelationships of the Stylidiaceae (Asterales), with a possible extreme case of floral paedomorphosis. Syst Bot 23: 289–304.

Lundberg J (2001) The Asteralean affinity of the Mauritian *Roussea* (Rousseaceae). Bot J Linn Soc 187: 267–276.

Manen JF, Cuenoud P, Partinez MDP (1998) Intralineage variation in the pattern of *rbcL* nucleotide substitution. Plant Syst Evol 211: 103–112.

Manns U, Bremer B (2010) Towards a better understanding of intertribal relationships and stable tribal delimitations within Cinchonoideae s.s. (Rubiaceae). Mol Phylogenet Evol 56: 21–39.

Michaels HJ, Scott KM, Olmsead RG, Szaro T, K JR, Palmer JD (1993) Interfamilial relationships of the Asteraceae: insights from *rbcL* sequence variation. Ann Mo Bot Gard 80: 742–751.

Morgan DR, Soltis DE (1993) Phylogenetic relationships among members of Saxifragaceae sensu lato based on *rbcL* sequence data. Ann Mo Bot Gard 80: 631–660.

Morton CM, Chase MW, Kron KA, Swensen SM (1996) A molecular evaluation of the monophyly of the order Ebenales based upon *rbcL* sequence data. Syst Bot 21: 567–586.

Morton CM, Mori SA, Prance GT, Karol KG, Chase MW (1997) Phylogenetic relationships of Lecythidaceae: A cladistic analysis using *rbcL* sequence and morphological data. Am J Bot 84: 530–540.

Mouly A, Razafimandimbison SG, Achille F, Haevermans T, Bremer B (2007) Phylogenetic placement of *Rhopalobrachium fragrans* (Rubiaceae): Evidence from molecular (*rps*16 and *trnTF*) and morphological data. Syst Bot 32: 872–882.

Mouly A, Razafimandimbison SG, Florence J, Jeremie J, Bremer B (2009) Paraphyly of *Ixora* and new tribal delimitation of Ixoreae (Rubiaceae): Inference from combined chloroplast (*rps16*, *rbcL*, and *trnT-F*) sequence data. Ann Mo Bot Gard 96: 146–160.

Nakamura K, Chung SW, Kokubugata G, Denda T, Yokota M (2006) Phylogenetic systematics of the monotypic genus *Hayataella* (Rubiaceae) endemic to Taiwan. J Plant Res 119: 657–661.

Novotny V, Basset Y, Miller SE, Weiblen GD, Bremer B (2002) Low host specificity of herbivorous insects in a tropical forest. Nature 416: 841–844.

Olmstead GR, Michaels JH, Scott MK, Palmer DJ (1992) Monophyly of the Asteridae and identification of their major lineages inferred from DNA sequenses of *rbcL*. Ann Mo Bot Gard 79: 249–265.

Olmstead RG, Bremer B, Scott KM, Palmer JD (1993) A parsimony analysis of the Asteridae sensu lato based on *rbcL* sequences. Ann Mo Bot Gard 80: 700–722.

Olmstead RG, Kim KJ, Jansen RK, Wagstaff SJ (2000) The phylogeny of the Asteridae sensu lato based on chloroplast *ndhF* gene sequences. Mol Phylogenet Evol 16: 96–112.

Olmstead RG, Reeves PA (1995) Evidence for the polyphyly of the Scrophulariaceae based on chloroplast *rbcL* and *ndhF* sequences. Ann Mo Bot Gard 82: 176–193.

Oxelman B, Backlund M, Bremer B (1999) Relationships of Buddlejaceae s.l. investigated using parsimony jackknife and branch support analysis of chloroplast *ndhF* and *rbcL* sequence data. Syst Bot 24: 164–182.

Piesschaert F, Andersson L, Jansen S, Dessein S, Robbrecht E, Smets E (2000) Searching for the taxonomic position of the African genus *Colletoecema* (Rubiaceae): morphology and anatomy compared to an *rps16*-intron analysis of the Rubioideae. Can J Bot 78: 288–304.

Plunkett GM, Soltis DE, Soltis PS (1996) Higher level relationships of Apiales (Apiaceae and Araliaceae) based on phylogenetic analysis of *rbcL* sequences. Am J Bot 83: 499–515.

Plunkett GM, Soltis DE, Soltis PS (1997) Clarification of the relationship between Apiaceae and Araliaceae based on *matK* and *rbcL* sequence data. Am J Bot 84: 565–580.

Razafimandimbison SG, Bremer B (2002) Phylogeny and classification of Naucleeae s.l. (Rubiaceae) inferred from molecular (ITS, *rbcL*, and *trnT-F*) and morphological data. Am J Bot 89: 1027– 1041.

Razafimandimbison SG, Kainulainen K, Wong KM, Beaver K, Bremer B (2011) Molecular support for a basal grade of morphologically distinct, monotypic genera in the species-rich Vanguerieae

alliance (Rubiaceae, Ixoroideae): its systematic and conservation implications. Taxon 60: 941–952.

Razafimandimbison SG, McDowell TD, Halford DA, Bremer B (2009) Molecular phylogenetics and generic assessment in the tribe Morindeae (Rubiaceae-Rubioideae): how to circumscribe *Morinda* L. to be monophyletic? Mol Phylogenet Evol 52: 879–886.

Razafimandimbison SG, Rydin C, Bremer B (2008) Evolution and trends in the Psychotrieae alliance (Rubiaceae) – a rarely reported evolutionary change of many-seeded carpels from one-seeded carpels. Mol Phylogenet Evol 48: 207–223.

Rova JHE, Delprete PG, Andersson L, Albert VA (2002) A *trnL-F* cpDNA sequence study of the Condamineeae-Rondeletieae-Sipaneeae complex with implications on the phylogeny of the Rubiaceae. Am J Bot 89: 145–159.

Rydin C, Kainulainen K, Razafimandimbison SG, Smedmark JEE, Bremer B (2008) Rare and enigmatic genera (*Dunnia*, *Schizocolea*, *Colletoecema*), sisters to species-rich clades: phylogeny and aspects of conservation biology in the coffee family. Mol Phylogenet Evol 48: 74–83.

Rydin C, Razafimandimbison SG, Khodabandeh A, Bremer B (2009) Evolutionary relationships in the Spermacoceae alliance (Rubiaceae) using information from six molecular loci: insights into systematic affinities of *Neohymenopogon* and *Mouretia*. Taxon 58: 793–810.

Savolainen V, Chase MW, Morton CM, Hoot SB, Soltis DE, Bayer C, Fay MF, de Bruijn A, Sullivan S, Qiu YL (2000a) Phylogenetics of flowering plants based upon a combined analysis of plastid *atpB* and *rbcL* gene sequences. Syst Biol 49: 306–362.

Savolainen V, Fay MF, Albach DC, Backlund A, van der Bank M, Cameron KM, Johnson SA, Lledó MD, Pintaud JC, Powell M, Sheahan MC, Soltis DE, Soltis PS, Weston P, Whitten WM, Wurdack KJ, Chase MW (2000b) Phylogeny of the eudicots: a nearly complete familial analysis based on *rbcL* gene sequences. Kew Bull 55: 257–309.

Sennblad B, Bremer B (1996) The familial and subfamilial relationships of Apocynaceae and Asclepiadaceae evaluated with *rbcL* data. Plant Syst Evol 129: 187–190.

Shinozaki K, Ohme M, Tanaka M, Wakasugi T, Hayashida N, Matsubayashi T, Zaita N, Chunwongse J, Obokata J, Yamaguchi-Shinozaki K, Ohto C, Torazawa K, Meng BY, Sugita M, Deno H, Kamogashira T, Yamada K, Kusuda J, Takaiwa F, Kato A, Tohdoh N, Shimada H, Sugiura M (1986). The complete nucleotide sequence of the tobacco chloroplast genome: its gene organization and expression. EMBO J 5: 2043–2049.

Spangler RE, Olmstead RG (1999). Phylogenetic analysis of Bignoniaceae based on the cpDNA gene sequences *rbcL* and *ndhF*. Ann Mo Bot Gard 86: 33–46.

Struwe L, Thiv M, Kadereit JW, Pepper ASR, Motley TJ, White PJ, Rova JHE, Potgeiter K, Albert VA (1998). Saccifolium (Saccifoliaceae), an endemic of Sierra de la Neblina on the Brazilian-Venezuelan border, is related to a temperate-alpine lineage of Gentianaceae Harv Pap Bot *3*: 199–214.

Thiers B (2008) [continously updated]. Index Herbariorum: A global directory of public herbaria and associated staff. New York Botanical Garden's Virtual Herbarium. http://sweetgum.nybg.org/ih/.

Wagstaff SJ, Olmstead RG (1997) Phylogeny of the Labiatae and Verbenaceae, inferred from *rbcL* sequences. Syst Bot 22: 165–179.

Wen H-Z, Wang R-J (2012) *Foonchewia guangdongensis* gen. et sp. nov. (Rubioideae: Rubiaceae) and its systematic position inferred from chloroplast sequences and morphology. J Syst Evol 50: 467–476.

Xiang Q, Soltis DE, Morgan DR, Soltis PS (1993) Phylogenetic relationships of *Cornus* L. sensu lato and putative relatives inferred from *rbcL* sequence data. Ann Mo Bot Gard 80: 723–734.
