# Supplementary material for: A Revised Time Tree of the Asterids: Establishing a Temporal Framework For Evolutionary Studies of the Coffee Family (Rubiaceae)
Source: PLoS One. 2015 May 21;10(5):e0126690. doi: 10.1371/journal.pone.0126690 (PMC4462594; doi:10.1371/journal.pone.0126690)
Supplement: S2 Table — (PDF) [file pone.0126690.s003.pdf]

S2 Table – Estimated ages for all nodes obtained in the separate and in the combined analyses

S2 Table. Estimated ages for all nodes obtained in the separate and in the combined analyses. Credibility intervals are indicated by 95% highest probability density (HPD) for each node. Node numbers sometimes differ between the two analyses but estimates given on the same row concern the same group of taxa in each of the analyses. Nodes present in the results of one of the analysis, but with no corresponence in the other, are indicated with nc. Node numbers in the separate analysis refer to numbered nodes in (Figs. 1 and 2) and node numbers in the combined analysis refer to numbered nodes in (Figs. 3 and 4). Node 42 (Gentianales crown group) and nodes 110, 111, and 112 all have two age estimates reported from the separate analysis, one from the asterid analysis and one from the analysis of the Gentianales.

	Ages from the separate analysis (Figs. 1 and 2)				Ages from the combined analysis (Figs. 3 and 4)		
Node number	Clade/taxon	Lineage age	95% HPD of lineage age	Node Number	Clade/taxon	Lineage age	95% HPD of lineage age
1		127	123-128	1		127	124-128
2	Asterids	125	121-128	2	Asterids	126	122-128
3		122	117-126	3		123	119-126
4	Core Asterids	116	109-122	4	Core Asterids	119	113-123
5	Lamiids	109	99-117	5	Lamiids	114	107-120
6		107	97-115	6		112	106-118
7	Core Lamiids	98	87-107	7	Core Lamiids	105	98-112
8		95	84-104	8		102	95-109
9		92	81-103	9		99	89-108
10	Solanales	82	64-96	10	Solanales	90	75-102
11		71	50-89	11		78	58-94
12		38	21-61	12		40	23-60
13		24	9-42	13		23	9-42
14		59	35-80	14		70	49-89
15		91	80-101	15		97	88-105
16	Lamiales	82	71-93	16	Lamiales	87	77-96
17		75	65-85	17		79	69-89
18		71	61-82	18		74	64-84
19		62	53-71	19		65	56-76
20		58	51-69	20		62	53-72
21		55	47-64	21		58	49-67
22		52	44-60	22		55	47-64
23		48	41-57	23		51	44-60
24		47	40-56	nc		nc	nc
25		40	30-51	24		44	33-54
26		38	27-48	25		40	28-51
27		25	13-37	26		26	13-38
nc		nc	nc	27		49	41-57
28		44	37-52	28		46	39-53
29		42	34-50	nc		nc	nc
nc		nc	nc	29		44	37-52
30		29	18-41	30		32	17-42
31		42	36-51	nc		nc	nc
nc		nc	nc	31		39	34-47
32		38	28-48	nc		nc	nc
nc		nc	nc	32		42	35-50

S2 Table – Estimated ages for all nodes obtained in the separate and in the combined analyses

Ages from the separate analysis (Figs. 1 and 2)				Ages from the combined analysis (Figs. 3 and 4)			
Node number	Clade/taxon	Lineage age	95% HPD of lineage age	Node Number	Clade/taxon	Lineage age	95% HPD of lineage age
33		39	28-48	33		39	34-46
34		46	34-57	34		50	38-61
38		48	35-61	38		50	37-62
40		49	30-64	40		52	32-68
41		36	17-57	41		39	18-61
42[1]	Gentianales	70	50-88	42	Gentianales	96	87-104
42[2]	Gentianales	96	82-110	-	–	–	–
43	Rubiaceae	92	78-106	43	Rubiaceae	87	79-96
44		80	63-98	44		78	67-89
45	Ixoroideae	64	45-82	45	Ixoroideae	59	48-73
46	Sabiceeae (stem)	60	43-78	46		57	46-70
47	Mussaendeae (stem)	56	40-74	nc		nc	nc
48	Stenisieae (stem)	50	35-67	47	Stenisieae (stem)	51	40-63
49	Retiniphylleae (stem)	41	29-57	48	Retiniphylleae (stem)	42	33-53
50	Vanguerieae + Coffeeae alliances	35	24-49	49	Vanguerieae + Coffeeae alliances	36	28-45
51	Airospermeae (stem)	30	19-44	50	Airospermeae (stem)	32	24-41
52	Augusteae (stem)	24	13-36	51	Augusteae (stem)	25	18-33
53	Alberteae (stem)	21	11-32	52	Alberteae (stem)	23	17-32
54		16	9-26	53		18	12-25
55	Octotropideae (stem)	14	7-23	54	Octotropideae (stem)	16	10-23
56		12	5-20	55		15	9-21
57	Gardenieae/Pavetteae (stem)	9	3-17	56	Gardenieae/Pavetteae (stem)	13	7-20
58	Sherbournieae/Cordiereae (stem)	9	4-17	57	Sherbournieae/Cordiereae (stem)	13	7-20
59	Coffeeae/Bertiereae (stem)	9	3-18	58	Coffeeae/Bertiereae (stem)	12	6-18
60	Crossopterygeae (stem)	31	21-43	59	Crossopterygeae (stem)	32	25-41
61	Jackieae (stem)	26	17-38	60	Jackieae (stem)	28	21-36
62	Scyphiphoreae (stem)	23	16-34	61	Scyphiphoreae (stem)	26	18-34
63	Traillaeodoxeae (stem)	21	13-32	62	Traillaeodoxeae (stem)	24	17-32
64		19	11-30	63	Vanguerieae (stem)	23	16-31
nc		nc	nc	64		22	15-30
65	Vanguerieae (stem)	17	10-27	nc		nc	nc
66	Greeneeae (stem)	13	6-21	65	Greeneeae (stem)	16	9-24
67	Ixoreae/Aleisanthieae (stem)	8	3-16	66	Ixoreae/Aleisanthieae (stem)	12	5-19
nc		nc	nc	67	Mussaendeae (stem)	54	41-67
68	Sabiceeae	13	3-34	68	Sabiceeae	16	5-33
69	Condamineae (stem)	48	21-75	69	Condamineae (stem)	56	43-70
70	Condamineae	18	4-43	70	Condamineae	19	7-35
71		11	3-29	71		16	5-32
72	Sipaneae (stem)	28	10-54	72	Sipaneae (stem)	41	20-59
73	Posoquerieae/Henriquezieae (stem)	13	3-33	73	Posoquerieae/Henriquezieae (stem)	21	7-39

S2 Table – Estimated ages for all nodes obtained in the separate and in the combined analyses

Ages from the separate analysis (Figs. 1 and 2)				Ages from the combined analysis (Figs. 3 and 4)			
Node number	Clade/taxon	Lineage age	95% HPD of lineage age	Node Number	Clade/taxon	Lineage age	95% HPD of lineage age
74	Cinchonoideae	58	42-76	74	Cinchonoideae	51	42-64
75		53	40-70	75		51	41-63
76		50	39-65	76		49	40-61
77	Chiococceae (stem)	38	15-56	77	Chiococceae (stem)	44	30-59
78	Hillieae/Hamelieae (stem)	17	5-38	78	Hillieae/Hamelieae (stem)	22	8-40
79	Hymenodictyeae/Nauc-leeae (stem)	38	34-48	79	Hymenodictyeae/Nauc-leeae (stem)	36	34-44
80	Rondeletieae/Guettar-deae (stem)	26	6-49	80	Rondeletieae/Guettar-deae (stem)	32	13-51
81	Isertieae/Cinchoneae (stem)	28	6-59	81	Isertieae/Cinchoneae (stem)	39	20-57
82	Luculieae (stem)	89	75-103	82	Luculieae (stem)	85	77-94
83	Coptosapelteae (stem)	86	73-101	83	Coptosapelteae (stem)	84	76-93
84	Rubioideae	76	63-90	84	Rubioideae	76	67-85
85		75	63-88	85		74	65-84
86	Ophiorhizeae (stem)	71	60-84	86	Ophiorhizeae (stem)	71	63-81
87	Coussareeae (stem)	63	53-74	87	Coussareeae (stem)	63	55-72
88	Psychotrieae + Spermacoceae alliances	60	50-70	88	Psychotrieae + Spermacoceae alliances	60	52-69
89	Spermacoceae alliance	48	36-59	89	Spermacoceae alliance	48	38-60
90	Anthospermeae (stem)	42	30-55	90	Anthospermeae (stem)	44	34-55
91		37	26-50	91		40	30-51
92	Argostemmateae (stem)	29	13-42	nc		nc	nc
nc		nc	nc	92		36	24-48
93	Dunnieae/Foonche-wieae (stem)	17	4-34	93	Dunnieae/Foonche-wieae (stem)	29	10-42
94	Paederieae (stem)	33	22-45	nc		nc	nc
nc		nc	nc	94	Paederieae (stem)	31	17-42
95	Putorieae (stem)	23	13-34	95	Paederieae (stem)	28	19-38
96	Rubieae/Theligoneae (stem)	18	9-29	96	Rubieae/Theligoneae (stem)	23	15-33
97	Danaideae (stem)	43	28-56	97	Danaideae (stem)	45	35-57
98	Knoxieae/Spermaco-ceae (stem)	35	22-50	98	Knoxieae/Spermaco-ceae (stem)	39	27-52
99	Spermacoceae	20	9-33	99	Spermacoceae	23	11-35
100	Psychotrieae alliance	54	46-63	100	Psychotrieae alliance	53	46-62
100	Schizocoleeae (stem)	54	46-63	100	Schizocoleeae (stem)	53	46-62
101	Craterispermeae (stem)	51	44-60	101	Craterispermeae (stem)	50	44-58
102	Prismatomerideae (stem)	49	43-57	102	Prismatomerideae (stem)	48	42-55
103		46	41-53	103		44	39-50
104	Schradereae (stem)	43	39-49	104	Schradereae (stem)	42	38-47
105	Gaertnereae (stem)	41	39-47	105	Gaertnereae (stem)	42	39-47
106	Mitchelleae/Morindeae (stem)	39	38-43	106	Mitchelleae/Morindeae (stem)	39	38-43
107	Palicourieae/Psychot-rieae (stem)	33	19-44	107	Palicourieae/Psychot-rieae (stem)	32	21-42
108	Psychotrieae	13	4-25	108	Psychotrieae	15	6-26

S2 Table – Estimated ages for all nodes obtained in the separate and in the combined analyses

Ages from the separate analysis (Figs. 1 and 2)				Ages from the combined analysis (Figs. 3 and 4)			
Node number	Clade/taxon	Lineage age	95% HPD of lineage age	Node Number	Clade/taxon	Lineage age	95% HPD of lineage age
109	Colletoecemateae/Lasiantheae (stem)	51	20-77	109	Colletoecemateae/Lasiantheae (stem)	67	52-81
110[1]		56	39-74	110		73	49-93
110[2]		61	35-89	–		–	–
111[1]		46	34-65	111		62	39-86
111[2]		45	25-73	–		–	–
112[1]		49	31-70	112		64	39-87
112[2]		43	19-73	–		–	–
113		104	93-114	113		110	100-117
114		96	76-110	114		102	74-115
116		104	86-117	116		110	95-119
117	Campanulids	110	100-118	117	Campanulids	112	104-119
118	Core Campanulids	99	89-109	118	Core Campanulids	101	90-110
119		96	87-107	119		99	89-110
120		91	77-105	nc		nc	nc
121		84	63-100	120		88	67-104
nc		nc	nc	121		81	53-100
122		80	60-97	nc		nc	nc
nc		nc	nc	122		81	56-100
123		70	47-91	nc		nc	nc
nc		nc	nc	123		97	86-107
124		92	82-104	124		94	83-104
126	Asterales	81	71-93	126	Asterales	82	71-94
127		78	68-89	127		78	68-91
128		72	62-83	128		73	62-84
129		70	61-80	129		71	61-82
130		64	56-74	130		65	56-76
131		54	49-62	131		55	49-63
132		49	47-55	132		49	47-56
133		22	8-39	133		22	7-40
134		62	39-78	134		63	43-80
135		56	30-73	nc		nc	nc
nc		nc	nc	135		57	33-75
136		94	82-107	136		96	84-107
137	Apiales	67	56-82	137	Apiales	67	56-83
138		61	49-79	138		61	49-79
139		49	38-64	139		49	41-64
140		44	30-61	140		43	30-61
141		23	10-45	141		22	8-41
143		90	77-103	143		92	79-104
144	Dipsacales	79	63-94	144	Dipsacales	80	65-94
145		59	47-74	145		62	49-76
146		50	40-63	146		52	41-64
147		38	34-46	147		39	34-48

S2 Table – Estimated ages for all nodes obtained in the separate and in the combined analyses

Ages from the separate analysis (Figs. 1 and 2)				Ages from the combined analysis (Figs. 3 and 4)			
Node number	Clade/taxon	Lineage age	95% HPD of lineage age	Node Number	Clade/taxon	Lineage age	95% HPD of lineage age
148		35	25-46	148		34	22-47
149		29	18-39	149		31	22-41
150		80	41-99	150		78	32-98
151	Aquifoliales	97	80-111	151	Aquifoliales	100	84-112
152		71	62-89	152		72	62-90
153		58	33-82	153		59	32-82
154	Ericales	111	104-119	154	Ericales	109	100-117
155		104	97-111	155		99	94-106
156		102	96-110	156		97	92-103
157		100	92-109	157		95	87-103
158		85	58-102	158		81	50-98
159		97	84-108	159		92	79-101
160		93	77-105	160		88	74-99
161		62	40-83	161		55	35-73
162		38	20-58	162		32	16-50
163		101	95-108	163		95	91-101
164		98	93-104	164		92	90-97
166		100	91-109	166		96	88-103
167		98	89-107	nc		nc	nc
168		89	78-99	167		86	77-95
169		77	72-88	168		76	72-85
170		73	72-88	169		71	44-89
nc		nc	nc	170		92	80-102
171		39	13-75	173		45	14-77
172		93	72-107	171		88	67-101
173		48	15-83	172		44	14-77
174		62	37-87	174		59	38-85
175		53	28-75	175		51	30-77
176		32	13-54	176		31	13-53
177	Cornales	103	90-118	177	Cornales	102	90-117
178		96	73-118	178		93	57-117

[1] Estimate obtained in the asterid analysis
[2] Estimate obtained in the Gentianales analysis
