# Supplementary material for: Correction: A Revised Time Tree of the Asterids: Establishing a Temporal Framework For Evolutionary Studies of the Coffee Family (Rubiaceae)
Source: PLoS One. 2016 Jun 3;11(6):e0157206. doi: 10.1371/journal.pone.0157206 (PMC4892603; doi:10.1371/journal.pone.0157206)
Supplement: S2 Table — This file includes the correct data. (PDF) [file pone.0157206.s001.pdf]

S2 Table – Estimated ages for all nodes obtained in the separate and in the combined analyses

S2 Table. Estimated ages for all nodes obtained in the separate and in the combined analyses. Credibility intervals are indicated by 95% highest probability density (HPD) for each node. Node numbers sometimes differ between the two analyses but estimates given on the same row concern the same group of taxa in each of the analyses. Nodes present in the results of one of the analysis, but with no corresponence in the other, are indicated with nc. Node numbers in the separate analysis refer to numbered nodes in (Figs. 1 and 2) and node numbers in the combined analysis refer to numbered nodes in (Figs. 3 and 4). Node 42 (Gentianales crown group) and nodes 110, 111, and 112 all have two age estimates reported from the separate analysis, one from the asterid analysis and one from the analysis of the Gentianales.

	Ages from the separate analysis (Figs. 1 and 2)				Ages from the combined analysis (Figs. 3 and 4)		
Node number	Clade/taxon	Lineage age	95% HPD of lineage age	Node Number	Clade/taxon	Lineage age	95% HPD of lineage age
1		126	123-128	1		127	124-128
2	Asterids	125	121-128	2	Asterids	125	122-128
3		122	117-126	3		123	119-126
4	Core Asterids	116	110-122	4	Core Asterids	118	113-123
5	Lamiids	110	101-118	5	Lamiids	114	107-119
6		108	99-116	6		112	106-118
7	Core Lamiids	99	90-108	7	Core Lamiids	105	98-112
8		97	88-105	8		102	95-110
9		94	85-103	9		99	89-107
10	Solanales	84	69-97	10	Solanales	89	75-102
11		72	51-90	11		77	59-93
12		38	20-57	12		42	23-61
13		23	9-41	13		25	9-43
14		63	40-84	14		69	48-87
15		92	83-101	15		98	89-106
16	Lamiales	83	74-94	16	Lamiales	88	77-98
17		76	66-86	17		80	68-91
18		72	61-81	18		75	63-86
19		63	53-71	19		66	56-76
20		59	50-67	20		63	53-72
21		55	46-63	21		59	50-69
22		52	44-60	22		56	47-65
23		48	41-56	23		52	44-61
24		47	39-54	nc		nc	nc
25		40	29-50	24		44	35-55
26		37	26-47	25		40	30-51
27		25	12-37	26		27	17-37
nc		nc	nc	27		50	42-59
28		43	36-51	28		47	40-56
29		41	34-48	nc		nc	nc
nc		nc	nc	29		46	38-54
30		28	17-40	30		32	17-46
31		42	35-50	nc		nc	nc
nc		nc	nc	31		41	34-48
32		38	28-47	nc		nc	nc
nc		nc	nc	32		44	36-52

S2 Table – Estimated ages for all nodes obtained in the separate and in the combined analyses

Ages from the separate analysis (Figs. 1 and 2)				Ages from the combined analysis (Figs. 3 and 4)			
Node number	Clade/taxon	Lineage age	95% HPD of lineage age	Node Number	Clade/taxon	Lineage age	95% HPD of lineage age
33		39	34-46	33		40	34-49
34		45	32-57	34		50	37-62
38		47	34-58	38		49	36-64
40		50	31-66	40		49	24-66
41		36	16-57	41		40	18-62
42[1]	Gentianales	75	60-91	42	Gentianales	96	86-104
42[2]	Gentianales	95	84-106	-	–	–	–
43	Rubiaceae	92	80-104	43	Rubiaceae	87	78-96
44		80	65-95	44		78	65-90
45	Ixoroideae	59	45-73	45	Ixoroideae	59	47-72
46	Sabiceeae (stem)	57	44-72	46		57	46-70
47	Mussaendeae (stem)	55	42-69	nc		nc	nc
48	Stenisieae (stem)	49	37-62	47	Stenisieae (stem)	51	40-63
49	Retiniphylleae (stem)	40	29-50	48	Retiniphylleae (stem)	41	32-52
50	Vanguerieae + Coffeeae alliances	33	25-42	49	Vanguerieae + Coffeeae alliances	35	27-43
51	Airospermeae (stem)	30	22-38	50	Airospermeae (stem)	31	23-39
52	Augusteae (stem)	24	17-31	51	Augusteae (stem)	25	17-32
53	Alberteae (stem)	22	15-30	52	Alberteae (stem)	23	16-31
54		17	11-23	53		17	12-24
55	Octotropideae (stem)	15	10-21	nc		nc	nc
56		14	9-20	54		14	9-20
57	Gardenieae/Pavetteae (stem)	9	3-17	55		13	6-19
58	Sherbournieae/Cordiereae (stem)	12	7-19	56	Sherbournieae/Cordiereae (stem)	13	7-19
nc		nc	nc	57	Octotropideae (stem)	16	10-22
59	Coffeeae/Bertiereae (stem)	11	6-18	58	Coffeeae/Bertiereae (stem)	11	5-18
60	Crossopterygeae (stem)	30	23-38	59	Crossopterygeae (stem)	31	24-39
61	Jackieae (stem)	26	19-34	60	Jackieae (stem)	27	20-34
62	Scyphiphoreae (stem)	25	18-32	61	Scyphiphoreae (stem)	25	18-32
63	Traillaeodoxeae (stem)	23	16-30	62	Traillaeodoxeae (stem)	24	17-31
64		22	15-29	63		22	15-29
65	Vanguerieae (stem)	22	15-29	nc		nc	nc
66	Greeneeae (stem)	16	9-22	64	Greeneeae (stem)	16	9-23
67	Ixoreae/Aleisanthieae (stem)	11	6-18	65	Ixoreae/Aleisanthieae (stem)	12	5-19
nc		nc	nc	66	Vanguerieae/*Glionnetia* (stem)	21	14-29
nc		nc	nc	67	Mussaendeae (stem)	54	41-67
68	Sabiceeae	16	5-31	68	Sabiceeae	18	6-32
69	Condamineae (stem)	55	41-71	69	Condamineae (stem)	56	44-70
70	Condamineae	19	7-34	70	Condamineae	21	9-38
71		17	6-31	71		19	6-34
72	Sipaneae (stem)	40	21-58	72	Sipaneae (stem)	42	24-59
73	Posoquerieae/Henriquezieae (stem)	22	8-38	73	Posoquerieae/Henriquezieae (stem)	23	8-39

S2 Table – Estimated ages for all nodes obtained in the separate and in the combined analyses

	Ages from the separate analysis (Figs. 1 and 2)				Ages from the combined analysis (Figs. 3 and 4)		
Node number	Clade/taxon	Lineage age	95% HPD of lineage age	Node Number	Clade/taxon	Lineage age	95% HPD of lineage age
74	Cinchonoideae	51	41-62	74	Cinchonoideae	51	40-62
75		50	40-61	75		50	40-61
76		49	39-59	76		49	39-60
77	Chiococceae (stem)	43	31-57	77	Chiococceae (stem)	44	31-57
78	Hillieae/Hamelieae (stem)	24	9-40	78	Hillieae/Hamelieae (stem)	23	8-40
79	Hymenodictyeae/Nauc-leeae (stem)	37	34-42	79	Hymenodictyeae/Nauc-leeae (stem)	37	34-43
80	Rondeletieae/Guettar-deae (stem)	31	12-49	80	Rondeletieae/Guettar-deae (stem)	33	15-51
81	Isertieae/Cinchoneae (stem)	40	21-57	81	Isertieae/Cinchoneae (stem)	38	20-55
82	Luculieae (stem)	90	78-103	82	Luculieae (stem)	85	76-95
83	Coptosapelteae (stem)	88	75-99	83	Coptosapelteae (stem)	84	76-94
84	Rubioideae	78	67-89	84	Rubioideae	75	66-84
85		76	65-86	85		74	65-83
86	Ophiorhizeae (stem)	73	63-83	86	Ophiorhizeae (stem)	71	62-80
87	Coussareeae (stem)	64	56-73	87	Coussareeae (stem)	62	54-70
88	Psychotrieae + Spermacoceae alliances	61	53-70	88	Psychotrieae + Spermacoceae alliances	59	52-67
89	Spermacoceae alliance	48	38-58	89	Spermacoceae alliance	47	39-57
90	Anthospermeae (stem)	43	33-54	90	Anthospermeae (stem)	43	34-53
91		40	29-50	91		39	30-48
92	Argostemmateae (stem)	34	21-46	nc		nc	nc
nc		nc	nc	92		36	27-46
93	Dunnieae/Foonche-wieae (stem)	28	13-42	93	Dunnieae/Foonche-wieae (stem)	28	12-42
94	Paederieae (stem)	37	26-47	nc		nc	nc
nc		nc	nc	94	Paederieae/Argostemmat eae (stem)	31	20-42
95	Putorieae (stem)	26	17-37	95	Putorieae (stem)	27	17-38
96	Rubieae/Theligoneae (stem)	22	13-32	96	Rubieae/Theligoneae (stem)	23	13-35
97	Danaideae (stem)	45	34-55	97	Danaideae (stem)	44	35-54
98	Knoxieae/Spermaco-ceae (stem)	39	29-51	98	Knoxieae/Spermaco-ceae (stem)	39	29-49
99	Spermacoceae	22	11-34	99	Spermacoceae	23	12-36
100	Psychotrieae alliance	55	48-63	100	Psychotrieae alliance	53	47-61
100	Schizocoleeae (stem)	55	48-63	100	Schizocoleeae (stem)	53	47-61
101	Craterispermeae (stem)	52	46-59	101	Craterispermeae (stem)	50	43-56
102	Prismatomerideae (stem)	50	44-56	102	Prismatomerideae (stem)	48	42-54
103		46	41-51	103		44	39-50
104	Schradereae (stem)	44	40-48	104	Schradereae (stem)	42	38-47
105	Gaertnereae (stem)	42	39-46	105	Gaertnereae (stem)	42	39-46
106	Mitchelleae/Morindeae (stem)	40	38-43	106	Mitchelleae/Morindeae (stem)	40	38-43
107	Palicourieae/Psychot-rieae (stem)	33	22-44	107	Palicourieae/Psychot-rieae (stem)	32	21-42
108	Psychotrieae	15	6-25	108	Psychotrieae	16	7-26

S2 Table – Estimated ages for all nodes obtained in the separate and in the combined analyses

Ages from the separate analysis (Figs. 1 and 2)				Ages from the combined analysis (Figs. 3 and 4)			
Node number	Clade/taxon	Lineage age	95% HPD of lineage age	Node Number	Clade/taxon	Lineage age	95% HPD of lineage age
109	Colletoecemateae/Lasiantheae (stem)	68	45-84	109	Colletoecemateae/Lasiantheae (stem)	65	49-80
110[1]		63	51-77	110		76	58-93
110[2]		68	52-84	–		–	–
111[1]		56	47-69	111		66	47-83
111[2]		57	47-72	–		–	–
112[1]		56	38-74	112		68	58-89
112[2]		56	29-80	–		–	–
113		105	95-115	113		109	98-117
114		95	76-112	114		101	83-114
116		103	87-117	116		109	97-118
117	Campanulids	110	101-118	117	Campanulids	111	102-119
118	Core Campanulids	100	89-110	118	Core Campanulids	100	90-111
119		97	86-107	119		98	89-108
120		91	75-106	nc		nc	nc
121		83	63-104	120		84	60-104
nc		nc	nc	121		77	53-98
122		79	58-100	nc		nc	nc
nc		nc	nc	122		77	50-99
123		69	40-91	nc		nc	nc
nc		nc	nc	123		95	85-106
124		93	81-104	124		93	82-103
125		50	17-79	125		51	27-78
126	Asterales	82	71-93	126	Asterales	83	71-94
127		79	68-90	127		79	67-89
128		74	64-85	128		73	62-83
129		71	62-82	129		71	60-81
130		66	56-75	130		65	57-75
131		55	49-62	131		55	49-63
132		50	47-55	132		50	47-55
133		24	9-40	133		23	9-38
134		60	33-78	134		64	47-80
135		54	27-74	nc		nc	nc
nc		nc	nc	135		58	37-75
136		95	83-106	136		95	83-107
137	Apiales	67	56-82	137	Apiales	68	56-81
138		62	48-78	138		62	50-76
139		50	38-65	139		50	41-61
140		45	29-61	140		43	31-57
141		24	9-42	141		27	9-51
143		91	79-103	143		91	77-104
144	Dipsacales	80	65-94	144	Dipsacales	79	63-93
145		61	47-75	145		60	46-75
146		52	40-64	146		51	39-63
147		40	36-49	147		40	36-48

S2 Table – Estimated ages for all nodes obtained in the separate and in the combined analyses

Ages from the separate analysis (Figs. 1 and 2)				Ages from the combined analysis (Figs. 3 and 4)			
Node number	Clade/taxon	Lineage age	95% HPD of lineage age	Node Number	Clade/taxon	Lineage age	95% HPD of lineage age
148		36	34-47	148		36	34-45
149		29	19-39	149		29	16-39
150		74	35-99	150		77	50-99
151	Aquifoliales	97	81-111	151	Aquifoliales	98	81-114
152		73	62-88	152		73	62-89
153		58	38-79	153		58	20-79
154	Ericales	108	100-116	154	Ericales	109	101-118
155		100	94-106	155		100	93-107
156		98	93-105	156		98	92-105
157		96	89-103	157		96	89-104
158		78	52-97	158		78	44-99
159		92	80-102	159		92	81-101
160		88	73-99	160		88	75-99
161		56	31-77	161		57	35-80
162		33	15-51	162		33	15-54
163		96	91-102	163		96	91-102
164		93	90-98	164		93	90-98
166		96	88-104	166		96	88-105
167		95	86-103	nc		nc	nc
168		86	77-96	167		87	77-96
169		77	72-85	168		77	72-86
170		68	35-92	169		68	38-90
nc		nc	nc	170		93	82-103
171		43	13-75	173		48	19-81
172		88	70-102	171		88	71-102
173		45	15-81	172		46	18-77
174		61	36-86	174		64	41-85
175		52	30-77	175		55	33-77
176		32	13-54	176		35	12-55
177	Cornales	103	90-117	177	Cornales	103	90-117
178		95	74-116	178		94	72-116

[1] Estimate obtained in the asterid analysis
[2] Estimate obtained in the Gentianales analysis
